# Supplementary material for: Combining cognitive bias modification training (CBM) and transcranial direct current stimulation (tDCS) to treat binge eating disorder: study protocol of a randomised controlled feasibility trial
Source: BMJ Open. 2019 Oct 22;9(10):e030023. doi: 10.1136/bmjopen-2019-030023 (PMC6830595; doi:10.1136/bmjopen-2019-030023)
Supplement: Supplementary data [file bmjopen-2019-030023supp001.pdf]

Supplementary File

Appendix A: ICARUS study task, questionnaire, measure and scale information

(1) *Approach bias assessment tasks*

(i) Food Approach-Avoidance Task (F-AAT) (Rinck & Becker, 2007): The F-AAT is a computerised task that measures approach and avoidance behaviour by means of joystick movements in response to food and neutral stimuli presented on a computer screen. This task will be used to assess approach bias towards visual cues of high-calorie food (expected target cognitive mechanism of intervention). Images of palatable edible foods (i.e. chocolate, pizza) and non-edible objects (i.e. sponges, stapler) are used as in previously (Brockmeyer et al., 2019). The assessment version of the AAT is identical to the treatment version except that the required response is unrelated to the picture content (i.e. food and neutral stimuli are presented equally often in round and rectangular, i.e. push and pull, format). Format movement assignments are counterbalanced among participants (i.e., half push round pictures and half push rectangular pictures). When the joystick is pulled, the picture grows bigger, and diminishes in size when the joystick is pushed. Zooming-in and zooming-out via joystick movements enacts motions of approaching and avoiding respectively and thus combines the proprioceptive (arm movement) and exteroceptive (zooming feature) cues of approach and avoidance behaviour (Neumann & Strack, 2000; Brockmeyer et al., 2019). The assessment version of the AAT consists of 80 trials (40 food item pictures and 40 non-food item pictures). To evaluate approach bias towards food, a compatibility score is calculated by subtracting the median reaction times (RTs) of compatible trials (i.e., RT pull food + RT push nonfood) from median RTs of incompatible trials (i.e., RT push food + RT pull nonfood) (Brockmeyer et al., 2019; Becker et al., 2016; Vrijsen et al., 2018). A positive value indicates a food-specific approach bias (i.e. the participant is faster at pulling than pushing food pictures, relative to the approach bias towards non-food), whereas a negative value indicates an avoidance bias. This task will be performed using Inquisit 5 (Millisecond Software).

(ii) The Stimulus Response Compatibility Task (SRC) (De Houwer, Crombez, Baeyens, & Hermans, 2001): In this task, participants respond to images on a computer screen by pressing keys on the keyboard. Pictures are presented in the centre of the screen with a manikin (12 mm high) positioned 33 mm above or below the picture. Participants are required to categorise the presented pictures by making an approach response (pressing the *up* or *down* key to move the manikin toward the picture) or an avoidance response (pressing the *up* or *down* key to move the manikin away from the picture). After making a correct response, an animation is shown of the manikin walking toward the picture (approach) or away from the picture (avoidance) for 1,000 ms. After making an incorrect response, a red cross appears on the screen for 500 ms, after which the next trial starts. Fourteen food and fourteen non-food pictures will be used from the food-pics database (Blechert, Meule, Busch & Ohla, 2014). The experiment comprises 8 practice trials and 56 experimental trials. Bias scores will be calculated by subtracting the mean of approach food/avoid non-food trials from the mean of avoid food/approach non-food trials. A positive score indicates a food-related approach bias, with higher scores indicative of stronger biases. This task will be performed using Inquisit 5 (Millisecond Software).

Gordon G, *et al. BMJ Open* 2019; 9:e030023. doi: 10.1136/bmjopen-2019-030023

(2) Food Choice Task

The Food Choice Task (Hare, Camerer & Rangel., 2009) adapted for eating disorders (Steinglass et al., 2015) is a computer-based paradigm that measures responses to images of foods to assess food attitudes and characteristics of eating behaviour. Food stimuli is used to investigate how individuals make decisions about what to eat, and measures decision-making around food by directly probing personal preferences. There are no learning requirements and individualised assessments of food along two dimensions (healthiness and tastiness) allow the tasks to be used in diverse populations with differing valuations of food. The task consists of three phases. In each phase participants are presented with 43 images of food items. The food items represent an array of dietary options (Steinglass et al., 2015). Twenty-five food items are low fat (<30% calories from fat) and 18 are high fat (>30% calories from fat), as determined by Foerde et al. (2018). In the Health phase, participants rate the healthiness of each food item on a 5-point scale (*1 = "Unhealthy", 5 = "Healthy"*). In the Taste phase, participants rate the tastiness of each food item in a similar fashion, (*1 = "Bad", 5 = "Good"*). In the Choice phase, in each trial participants choose between the presented food item and a "Neutral" reference food item (rated as 3 in both Health and Taste phases). If no item is rated 3 on both scales, an item rated 3 on Health and greater than 3 on the Taste scale is selected as a reference food. Good test-retest reliability of the FCT suggests that it is suitable for measuring food-based decision-making in studies with multiple assessment points (Foerde et al., 2018).

(3) *State food craving - Food craving after cue exposure task*

The Food Challenge Task (FCT) (Kekic et al., 2017; Meule et al., 2018) will be used to examine cue-induced food craving. In this task, participants rate their state food craving using the Food Cravings Questionnaire State Version (Cepeda-Benito et al., 2000; Meule et al., 2012) before and after being presented with a video on a computer screen of foods shown to be highly appetitising and to elevate hunger levels (Kekic et al., 2017). The questionnaire consists of 15 items that measure the strength of food cravings (i.e. *I would feel more alert if I could satisfy my craving*). Participants are asked to indicate how much they agree with each statement 'at this very moment' using a five-point scale (from 1 'strongly disagree' to 5 'strongly agree'). There are five craving subscales; intense desire to eat, anticipation of relief from negative states, physiological craving, preoccupation with food or lack of control over eating and anticipation of positive reinforcement. Scores can be calculated for specific subscales or a total score can be calculated (ranging from 15 to 75). This questionnaires will be completed using Inquisit 5 (Millisecond Software), and the video shown using QuickTime player.

(4) *Trait food craving*

The Yale Food Addiction Scale Version 2.0 (YFAS 2.0; Gearhardt, Corbin, & Brownell, 2016) is the most commonly used instrument to assess food-related addictive behaviours (Steward et al., 2018). This self-report questionnaire consists of 35 items scored on an 8-point Likert scale (from 0 = never to 7 = every day) and is adapted to assess addictive eating behaviours based on DSM-5 substance-related and addictive disorders criteria (APA, 2013). It refers specifically to consumption of foods high in fat, sugar, salt or refined carbohydrates. It

Gordon G, *et al. BMJ Open* 2019; 9:e030023. doi: 10.1136/bmjopen-2019-030023

includes items that assess specific criteria, such as diminished control over consumption, a persistent desire or repeated unsuccessful attempts to quit, withdrawal, and clinically significant impairment (i.e. *'I kept eating in the same way even though my eating caused emotional problems')*. The YFAS includes two scoring options: 1) a "symptom count" ranging from 0 to 7 that reflects the number of addiction-like criteria endorsed and 2) a categorical scoring option that classifies respondents as having either no, mild, moderate or severe 'food addiction'. The YFAS has received psychometric support in binge eating populations (Gearhardt, White et al., 2013; Carter, Van Wijk & Rowsell, 2019), and obese bariatric surgery patients (Clark & Saules, 2013; Meule, Heckel, & Kübler, 2012). The YFAS 2.0 was developed to maintain consistency with the current diagnostic understanding of addiction and to improve the psychometric properties of the original YFAS. Exceeding the food addiction threshold was more strongly associated with obesity for the YFAS 2.0 than the original YFAS. The YFAS 2.0 has demonstrated good internal consistency (Carter, Van Wijk & Rowsell, 2019), as well as convergent, discriminant and incremental validity (Gearhardt, Corbin & Brownell, 2016).

(5) *Food intake in a Bogus Taste Test*
Actual food consumption will be measured by means of a Bogus Taste Test (Robinson et al., 2017); a food consumption test presented under the guise of a taste test. Participants will be instructed to rate 3 bowls of highly palatable high-calorie food items (chocolate, sweets, crisps) in terms of their visual attractiveness, smell, and taste on a paper form. The researcher will inform the participant that she/he will leave the room for 10 minutes and during this time they can complete their ratings and are free to eat as much of the offered items as they like. A small bin with lid will be provided and participants will be instructed to consume as much food as they need or want, and to discard the remainder of the food items in the bin before 10 minutes are over. After the participant has left, the discarded food items will be recovered from the bin, sorted, and placed in their original bowls. Consumption will be determined by weighing the bowls both before and after the "taste test" and the difference in weight from pre-to post-assessment will be converted into calories and used as a measure of food intake.

(6) *Delay discounting*
Delay Discounting Task with Money and Food (Odum, Baumann, & Rimington, 2006): Participants indicate their preferences in a series of choices for two hypothetical outcome types: immediate versus delayed food and immediate versus delayed money. Participants make choices involving either relatively small maximum amounts of food (10 dollars worth) and money (10 dollars) or for relatively large maximum amounts of food (100 dollars worth) and money (100 dollars). Performance on this task can be used to study self-regulation, delayed gratification and valuation of reward. This task will be performed using Inquisit 5 (Millisecond Software).

(7) *Inhibitory control*
(i) Go/No-Go task: The cued go/no go task is a useful measure of impulse control in clinical populations. This task is a classic test of executive function, requiring effortful response inhibition. The food specific version of the cued go no-go task (Teslovich et al., 2014) measures impulsivity and response inhibition with respect to appetising food and nonfood items (i.e. toys), via assessing the ability to inhibit instigated, prepotent responses. The task

Gordon G, *et al. BMJ Open* 2019; 9:e030023. doi: 10.1136/bmjopen-2019-030023

manipulates response prepotency by presenting a preliminary go or no-go cue before the actual go or no-go target is displayed. The cues provide information concerning the probability that a go or no-go target will be presented. The cue-target relationship is manipulated so that the cues have a high probability of correctly signaling a go or no-go target (valid cues), and a low probability of incorrectly signaling a target (invalid cues). Valid cues tend to facilitate response inhibition and speed response execution, whereas invalid cue cues tend to impair response inhibition and slow response execution. The set of stimuli consists of 30 colour images of common high- (8) and low-calorie (7) foods and common toys (15). The outcome variables include: (1) overall reaction time (RT) in milliseconds during correct “go” trials, (2) rate of omission errors (missed “go” trials), and (3) false alarm rate (rate at which participants erroneously press to a no-go stimulus). This task will be performed using Inquisit 5 (Millisecond Software).

(i) Stop Signal Task (SST): This is a task measuring inhibitory control. Participants are required to engage in a computer task but withhold their response in the presence of a stop signal. This SST includes food-specific and neutral non-food stimuli in the same task (adaptation of Manasse et al., 2016). This allows for isolation of any unique food specific inhibitory control deficits from general difficulties inhibiting responses. The outcome measure is the stop signal reaction time (SSRT). The SSRT is calculated for each set of stimuli (i.e., SSRT stimulus type) for each subject by subtracting the average stop signal delay from the average reaction time on “go” trials (Verbruggen & Logan, 2008). The recording accuracy of reaction time and stop signal delay measurement is in milliseconds. A smaller SSRT is indicative of greater inhibitory control and a larger SSRT reflects weaker/impaired inhibitory control. This task will be performed using Inquisit 5 (Millisecond Software).

(8) *Mood and emotion regulation*
The Emotion Regulation Questionnaire (ERQ) (Gross & John, 2003): A 10-item scale designed to measure respondents’ tendency to regulate their emotions in two ways: (1) cognitive reappraisal and (2) expressive suppression. Respondents answer each item on a 7-point Likert-type scale ranging from 1 (strongly disagree) to 7 (strongly agree). This will be administered at pre-assessment, post-assessment and follow-up.

Positive and Negative Affect Schedule (PANAS) (Watson, Clarke & Tellegen, 1988): The PANAS consists of two 10-item self-report scales which measure positive and negative affect. On a Likert scale ranging from 0 (very slightly or not at all) to 4 (extremely), participants rate the extent to which they have experienced each of the 20 descriptors within a particular time frame (“right now” in the current study). Two scores are generated: positive (PANAS-positive) and negative (PANAS- negative) affect. This will be administered at pre-assessment, post-assessment and follow-up.

Depression, Anxiety and Stress Scale (21-item version; DASS-21) (Lovibond & Lovibond, 1995): This is a 21 item self-report questionnaire which aims to evaluate mood, anxiety and stress levels over the previous week. The DASS-21 will be administered at pre-assessment, post-assessment and follow-up.

(9) *Within session measures (immediately after each [ABM+real/sham tDCS] treatment session)*

(i) Paper-based Visual Analogue Scales (VAS) assessing current hunger, feeling of fullness, urge to eat, urge to binge eat, feeling low, level of tension, level of stress, level of anxiety. These scales consist of a 10cm line. Participants are requested to indicate on this line a degree or level of a specific emotion or behavioural urge. There are indications of what range (e.g. from 'not at all' to 'extremely').

(ii) Tolerance, discomfort and side effects: An evaluation of discomfort with the training session (tDCS and ABM aspects separately) will be completed with another paper-based 10cm VAS (rated from none to extreme discomfort). Participants will be asked to report any side effects in an open ended question.

Appendix B: Information sheet for all participants

PARTICIPANT INFORMATION SHEET

ICARUS: An Investigation of Approach Bias Modification Training (ABM) and Transcranial Direct Current Stimulation (tDCS) in Binge Eating Disorder

IRAS Project ID: 244170
Research Ethics Committee reference number: 18/NW/0648

We would like to invite you to participate in this postdoctoral research project which is being conducted by a PhD student for research and educational purposes. You should only participate if you want to; choosing not to take part will not disadvantage you in any way. Before you decide whether you want to take part, it is important for you to understand why the research is being done and what your participation will involve. Please take time to read the following information carefully and discuss it with others if you wish. Part 1 tells you the purpose of this study and what will happen to you if you take part. Part 2 gives you more detailed information about the conduct of the study. Please ask us if there is anything that is not clear or if you would like more information.

PART ONE

What is the purpose of the study?
Psychological therapy as a main treatment for Binge Eating Disorder (BED) may not be effective for many people and may not be readily accessible in some areas. Medical treatments for BED can have side effects and often do not remain effective in the long-term. Therefore, there is an ongoing need for the development of new treatments.

Computerised approach bias modification training (ABM) is a specific form of cognitive bias modification (CBM) that has been used to successfully treat mental disorders such as anxiety, depression, and addictive disorders. This technique involves several sessions of computerised training, a procedure which has shown to be effective in reducing the severity of some eating disorder symptoms in people with BED and Bulimia Nervosa (BN). ABM works as such; automatic approach and avoidance tendencies towards food-related cues are modified by repeated training of arm movements in front of a computer

screen. ABM has shown to reduce approach tendencies and attention towards food cues in a subclinical sample of eating disorders involving binge eating, but its efficacy on these features in people with full-syndrome eating disorders remains unclear. Further research is needed to examine if ABM is effective in reducing the frequency of binge eating episodes in people with BED.

Transcranial direct current stimulation (tDCS) is a non-invasive technique that is capable of stimulating specific brain areas. Research shows that the frontal areas of the brain play a role in the development and maintenance of eating disorders, including BED. Stimulating these brain areas to alter their functioning is therefore believed to have the potential to reduce eating disorder symptoms. This involves the delivery of a low electrical current via small electrodes placed on the scalp. This procedure is widely used in research and is being applied in clinical settings. Recent research using tDCS on people with BED has suggested that it may be helpful in reducing immediate food intake and cravings, and may decrease the frequency of a desire to binge eat at home after the treatment.

Combining ABM and tDCS

Previous studies suggest that these two techniques potentially help people better regulate their behaviours through similar mechanisms in the brain. Delivering both treatments together at the same time may have a stronger effect on reducing eating disorder symptoms in people with BED than either of the treatments alone. This will be the first time that this specific combination of interventions is conducted on people with an eating disorder.

In the present study, we aim to investigate combined ABM and tDCS as a treatment for BED by comparing the effect of 6 sessions of (ABM + real tDCS) vs. (ABM + placebo tDCS) across a 3-week period in adult men and women with BED. We will also compare these two groups against a control group. Participants will be allocated by chance to either one of two intervention groups, or to the control group. Participants in the intervention groups will receive 6 sessions of ABM delivered simultaneously with either real tDCS or a placebo version of tDCS. Participants assigned to the control group will not receive any intervention. We will measure eating disorder symptoms and other outcomes in all participants at baseline, post-treatment, and at the 4-week follow-up to assess outcomes of each study group. In particular, we are interested in changes in the frequency of binge eating and craving, and thought processes and emotions related to food and eating. We will also ask participants about their experience of this treatment. Participants assigned to the control condition will be offered 6 sessions of (ABM + real tDCS) after they have completed their involvement in the study.

Why have I been invited?

You are invited to participate if you are a male or female aged between 18 and 70 who has a current diagnosis of binge eating disorder (BED). We will be recruiting 66 participants in total.

Do I have to take part?

You do not have to take part in this experiment; it is your choice. If you decide to take part, you will be asked to sign three identical consent forms. You will be free to

Gordon G, *et al. BMJ Open* 2019; 9:e030023. doi: 10.1136/bmjopen-2019-030023

withdraw from the study at any time without giving a reason. Whether you decide to take part or not will in no way influence your care or the timing of your treatment.

What will happen to me if I take part and what will I have to do?
If you decide you want to participate you will firstly be asked to engage in a telephone conversation with the researcher (lasting approximately 20 minutes) to confirm that you are eligible to take part. If you are, you will be invited to the Institute of Psychiatry, Psychology and Neuroscience (Kings College London, Denmark Hill Campus) for a baseline assessment session on a day that is convenient for both you and the researcher, in either the morning or the afternoon. On this day, the researcher will discuss the study with you in person, answer your questions, and if you are happy to take part, we will ask you to sign three copies of a consent form: one for you to keep, one for us to keep, and one that will be sent to your general practitioner (GP).

This baseline assessment visit is longer that the treatment visits and is comprised of an in-person visit and online questionnaires to complete at home. During the visit, you will give informed study consent, complete two questionnaires, neuropsychological tasks (brain puzzles), and a food task for which you will be asked to rate different foods. This visit also involves an assessment version of the approach bias modification (ABM) training programme, which is a computer based-task involving pushing and pulling a joystick in response to shapes appearing on the computer screen. Weight and height will be measured at each assessment, and participants may choose not to see the figures recorded. Assessment visits will last 65-75 minutes. Within 36 hours after the in-person visit, you will be emailed a link to a series of questionnaires (assessing mood and eating disorder-related thoughts and habits) which will take 30-45 minutes to complete. There is also the option to complete these assessment questionnaires in hardcopy paper format.

You will then be then randomised and informed of your randomly assigned study condition within a week. If you are assigned to one of the two intervention conditions, you will be asked to attend 6 sessions where you will perform the ABM task while receiving either real or placebo tDCS, in addition to completing the post-treatment and follow-up assessments. If you are assigned to the control group, you will not receive any intervention, and will be asked to attend the post-treatment and follow-up assessments.

All 6 intervention sessions will be identical and last approximately 40–50 minutes. There is no need for any special preparation before the visits. Before and after each intervention session, you will complete some scales related to aspects of your mood and level of hunger. Your blood pressure and pulse will also be measured before and after each intervention session to monitor your wellbeing.
During the (ABM + real/placebo tDCS) session you will sit on a comfortable chair facing a computer screen, with a joystick on a table in front of you. You will wear a plastic headband to keep the two tDCS electrodes in place (as shown in the diagram below). The electrodes will be placed in small sponges soaked in a salt water solution, so they might feel a bit wet against your head. The researcher will turn the machine on which will deliver the currents. Depending on your assigned study condition, you will receive real or placebo brain stimulation. The placebo session will be the same as the real session, but the tDCS machine won't deliver any electrical current. Most people can't tell the difference between real and placebo tDCS sessions.

Gordon G, *et al*. *BMJ Open* 2019; 9:e030023. doi: 10.1136/bmjopen-2019-030023

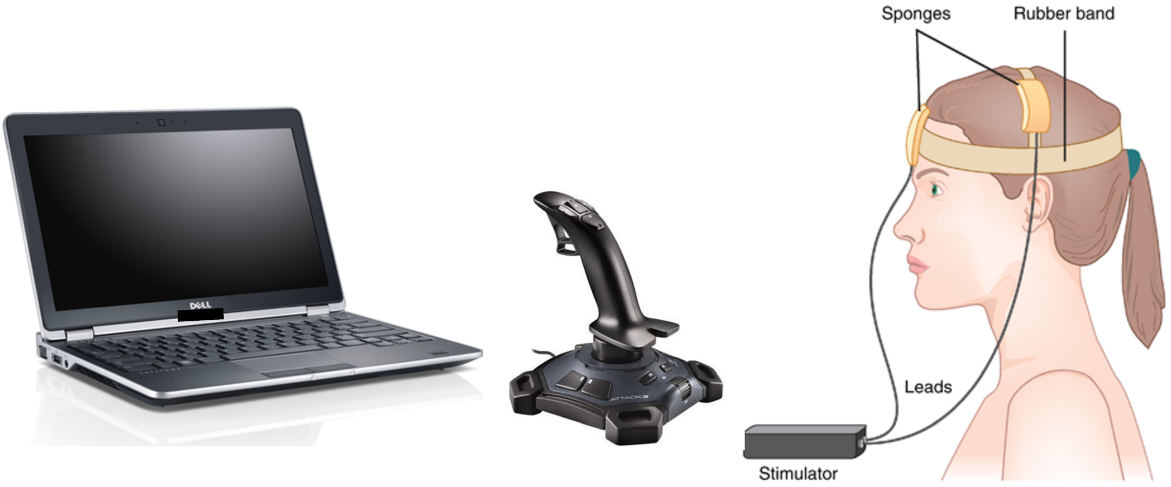

The tDCS will begin a few minutes prior to the start of the computer training programme, to allow participants to become used to the sensation before starting the ABM task on the computer. The training version of the ABM programme is a computer based-task involving pushing and pulling a joystick in response to shapes appearing on the computer screen. The tDCS will also continue for a few minutes after the ABM task has ended. You will then be asked to rate any discomfort experienced during the session due the tDCS.

You will be asked to return to the Institute for 5 more identical intervention sessions within 3 weeks, leaving a gap of at least 24 hours between each session. The post-assessment will be conducted immediately after the 6th intervention session. This in-person visit comprising of the final intervention session plus the post-assessment will therefore last 90-120 minutes, with the online questionnaires to be completed within 36 hours after this visit (45 minutes). The follow-up assessment 4 weeks later will be similar to the baseline assessment. This final visit will therefore take 75 minutes, with the online questionnaires taking 45 minutes to complete at home afterwards.

20 participants (10 participants from each intervention condition) will be invited to provide feedback on their experiences of study participation, initial expectations of the intervention, perceived strengths and weaknesses, and suggestions for improvements in an interview with the researcher. Declining this invitation to interview does not affect study payment.

6 months after the baseline assessment, participants may be contacted by phone by a member of the King's College London Eating Disorders Unit for a brief phone call to evaluate the presence of eating disorder symptoms. This 6 month check-in will allow any long-term therapeutic effects of the study treatment intervention to be evaluated. Participation in this phone call will not affect participant payment, which will have been administered earlier after the 1-month follow up assessment.

Expenses and payments

Upon completion of the study, all participants will be paid a maximum of £60 for completing each of the three assessment sessions (comprised of an in-person visit and at-home questionnaires); the baseline assessment, post-treatment assessment and follow-up assessment (£20 each). This payment should be declared for tax and/or benefit purposes. If you are assigned to an intervention condition involving 6 sessions of brain stimulation and computer training, you may also be compensated up to £10 per day for your travel expenses on these intervention session days.

What is expected from you as a participant?
We would expect you to complete all assessment sessions (pre-, post-treatment, follow-up), and if you are randomised to an intervention group, to attend all 6 (ABM + real/placebo tDCS) sessions as scheduled. We ask you to inform us immediately if for any reason you suddenly find yourself unable to attend a scheduled session.

Please let us know of any health problem that has developed, or any new diagnosis made since you enrolled for the study. Further, we would ask you to let us know of any new medication or change in medication whilst you are taking part in the study.

What are the possible disadvantages and risks of taking part? What are the side effects?
Combined brain stimulation and cognitive training sessions are time-consuming and may cause fatigue from concentrating on the task.

TDCS has been shown to be safe when used correctly in a clinical setting. However, you may find the procedure slightly uncomfortable. This is because a number of sensations can occur beneath the electrodes during stimulation including tingling, pain, itching, and burning. Not everyone feels these sensations or finds them uncomfortable, but if you do, remember you are free to stop the study at any point without giving an explanation. In some rarer cases, tDCS has been known to cause a headache, but this can be treated with mild painkillers (e.g. paracetamol). No side effects of ABM are known. We will assess any discomfort you may experience during intervention sessions throughout your involvement in the trial.

What are the possible benefits of taking part?
Unfortunately, there are no direct benefits to taking part in this study, but the information we get may help us to improve the treatment of BED in the future.

What happens when the research study stops?
When the research study stops, no further ABM + tDCS sessions will be available to those who have received 6 sessions. Participants in the control group will have the option of receiving 6 sessions of [ABM + real tDCS] once they have completed the waiting period and follow-up assessment.
Participants assigned to one of the intervention groups can request to be informed if they had received ABM combined with real or placebo tDCS, once they have completed the follow-up assessment.

What if there is a problem?
Any complaint about the way you have been dealt with during the study or any possible harm you might suffer will be addressed. Detailed information on this is given in Part 2.

Gordon G, *et al. BMJ Open* 2019; 9:e030023. doi: 10.1136/bmjopen-2019-030023

Will my taking part in the study be kept confidential?
Yes. We will follow ethical and legal practice and all information about you will be handled in confidence. The details are included in Part 2.

If the information in Part 1 has interested you and you are considering participation, please read the additional information in Part 2 before making a decision.

PART TWO

What if relevant and new information becomes available?
Sometimes we get new information about the treatment being studied. This is not expected to occur given the short time frame of participation (6 sessions across 3 weeks); however, if any new and relevant information becomes available during this time we will inform you immediately. You can then decide whether you wish to continue in the study.

What will happen if I don't want to carry on with the study?
Your participation is voluntary and you do not have to take part in the study. You can change your mind at any point and terminate your participation without giving a reason to the researcher. You are free to withdraw from this study at any time without consequence.

Will participation in this study affect my routine healthcare, or the waiting period for treatment for my eating disorder if I am currently on a waiting list?
No. Participation in this study will have no impact on your treatment as usual, or waiting time if you are currently awaiting treatment for your eating disorder. We fully encourage you to begin treatment as provided by a health care professional as soon as it becomes available to you. We simply ask that you inform us of any changes to your treatment or medication while you are partaking in the study.

What if there is a problem?
If you have a concern about any aspect of the study, please ask the researcher (gemma.gordon@kcl.ac.uk, 0207 848 0183) who will do their best to answer your questions.

What if I wish to make a complaint?
If you remain unhappy and wish to formally complain, complaints to the IoPPN should be addressed to Dr Gill Dale. Director of Research Quality; Head, Joint R&D Office of South London and Maudsley NHS Foundation Trust and Institute of Psychiatry, Psychology & Neuroscience (IoPPN), P005, Institute of Psychiatry, Psychology & Neuroscience (IoPPN), King's College London, De Crespigny Park, London SE5 8AF. NHS complaints will follow NHS complaints procedures.

Other sources of support for your eating disorder
To access support and treatment, please see your Eating Disorders clinician or your GP who will be able to advise you and refer you to the right service for you. You can also

Gordon G, *et al. BMJ Open* 2019; 9:e030023. doi: 10.1136/bmjopen-2019-030023

obtain further information and support from www.beateatingdisorders.org.uk, the national Eating Disorders charity.

Should you wish to speak to someone outside of the university, please talk to your Eating Disorders clinician, GP, the Beat helpline, and/or one of the study researchers who is happy to liaise on your behalf if you so wish. The eating disorders charity Beat provides helplines for adults and young people which offer support and information to sufferers, carers and professionals. Further information can be found on their website www.beateatingdisorders.org.uk, or by ringing their helpline 0808 801 0677**.**

Will my taking part in the study be kept confidential?
Your personal information and the data we collect from you will remain confidential at all times. It will also remain anonymous to everyone apart from the primary researchers. Manual files will be locked securely in a filing cabinet, which will be kept in a locked office in the KCL Section of Eating Disorders, Department of Psychological Medicine, IoPPN, and all electronic files will be password protected. All information which is collected during the course of the research will be kept strictly confidential according to the General Data Protection Regulation (GDPR), brought into effect on 25th May, 2018. This new legislation creates some new rights for individuals to better reflect data protection challenges in the modern digital age, as well as strengthening some of the rights that currently exist under the Data Protection Act 1998.

How will my personal data be used and what are my rights?
King's College London is the sponsor for this study based in the United Kingdom. We will be using information from you in order to undertake this study and will act as the data controller for this study. This means that we are responsible for looking after your information and using it properly. King's College London will keep identifiable information about you for four years after the study has finished.
Your rights to access, change or move your information are limited, as we need to manage your information in specific ways in order for the research to be reliable and accurate. If you withdraw from the study, we will keep the information about you that we have already obtained. To safeguard your rights, we will use the minimum personally-identifiable information possible. You can find out more about how we use your information by contacting the Study Coordinator Gemma Gordon.

KCL will use your name and contact details to contact you about the research study, and make sure that relevant information about the study is recorded for your care, and to oversee the quality of the study. Individuals from KCL and regulatory organisations may look at your medical and research records to check the accuracy of the research study. SLaM will pass these details to KCL along with the information collected from you. The only people in KCL who will have access to information that identifies you will be people who need to contact you regarding your participation or audit the data collection process. The people who analyse the information will not be able to identify you and will not be able to find out your name or contact details. KCL will keep identifiable information about you from this study for four years after the study has finished.

Involvement of the General Practitioner (GP)

As a matter of courtesy and in the interest of your wellbeing, we may let your GP know about your participation in the study, and may request your permission to send them a letter when you enroll. If you agree to this, you will be asked to provide us with your GP's contact details so that we can send them a letter with details of the research.

Insurance/indemnity
Standard KCL insurance and NHS indemnity arrangements apply.

Involvement of the insurance company
If you have private medical insurance, you should inform your insurance company that you are taking part in this study.

Will any genetic tests be done?
No.

What will happen to the results of the research study?
You will be offered the opportunity to be informed about your individual results once the data for all participants has been collected. If you want written feedback of the study's findings you can contact the researcher (gemma.gordon@kcl.ac.uk) for a lay summary. The results will be included in an examined postgraduate report, presented as part of a postgraduate presentation, and sent to a medical journal for publication. Your participation in the study will not be disclosed.

Who is organising and funding the research?
This study is being funded by King's College London.

Who has reviewed the study?
All research in the NHS is looked at by an independent group of people, called a Research Ethics Committee, to protect your interests. This study has been reviewed and given favourable opinion by North West -Liverpool East Research Ethics Committee.

Further information and contact details
If you have any questions or require more information about this study, please contact the researcher using the following contact details:
Gemma Gordon (gemma.gordon@kcl.ac.uk) (0207 848 5608)
Section of Eating Disorders, Department of Psychological Medicine
KCL Institute of Psychiatry, Psychology and Neuroscience,
16 De Crespigny Park
London, SE5 8AF

Appendix C: Consent form for all participants

CONSENT FORM

IRAS Project ID: 244170
Research Ethics Committee reference number: 18/NW/0648

Please complete this form after you have read the information

KING'S College LONDON

12

sheet and listened to an explanation about the research.

Title of Study: ICARUS - An Investigation of Approach Bias Modification Training (ABM) and Transcranial Direct Current Stimulation (tDCS) in Binge Eating Disorder
Name of Researcher: Gemma Gordon

South London and Maudsley NHS Foundation Trust

Thank you for considering taking part in this research. The person organising the research must explain the project to you before you agree to take part. If you have any questions arising from the information sheet or explanation already given to you, please ask the researcher before you decide whether to join in. You will be given a copy of this consent form to keep and refer to at any time.

Please initial box

1. I confirm that I have read the information sheet dated 18.09.2018 (version 3) for the above study. I have had the opportunity to consider the information, ask questions and have had these answered satisfactorily. ☐
2. I understand that my participation is voluntary and that I am free to withdraw at any time without giving a reason, without my medical care or legal rights being affected. ☐
3. I consent to the processing of my personal information for the purposes explained to me. I understand that such information will be handled in accordance with the terms of the General Data Protection Regulation (GDPR) 2018. I understand that confidentiality and anonymity will be maintained and it will not be possible to identify me in any publications. ☐
4. I know that if I would like to, I can contact the research team and request a written summary of the study findings. ☐
5. I understand that I must not take part if I have any of the conditions listed in the exclusion criteria. ☐
6. I understand that during study participation, I must inform the researcher of any changes in my medication or of any new medical diagnoses made. ☐
7. I agree to my General Practitioner (GP) being informed of my participation in this study. ☐
8. **I consent/do not consent** to be contacted in the future by King's College London researchers who would like to invite me to participate in follow up studies to this project, or in future studies of a similar nature. ☐
9. I agree to take part in the above study. ☐

<u>Participant's Statement:</u>

I ____
agree that the research project named above has been explained to me to my satisfaction and I agree to take part in the study. I have read both the notes written above and the Information Sheet about the project, and understand what the research study involves.

Signed Date

<u>Investigator's Statement:</u>

I ____
Confirm that I have carefully explained the nature, demands and any foreseeable risks (where applicable) of the proposed research to the participant.

Signed Date

<u>For administration purposes, please indicate your preference below</u>

Q: In what format would you like to complete the at-home assessment questionnaires?

Tick '√' in one box to indicate your choice

1. I would like to complete the assessment questionnaires online ☐

2. I would like to receive a hardcopy paper version of the questionnaires in a stamped addressed envelope, to complete and post back to the researcher within 36 hours of the in-person assessment visit. ☐

Enquiries:
Gemma Gordon (gemma.gordon@kcl.ac.uk)
Department of Psychological Medicine
Section of Eating Disorders
KCL Institute of Psychology, Psychiatry and Neuroscience,
16 De Crespigny Park
London, SE5 8AF
Phone: 0207 848 5608
