# Supplementary material for: Combining cognitive bias modification training (CBM) and transcranial direct current stimulation (tDCS) to treat binge eating disorder: study protocol of a randomised controlled feasibility trial
Source: BMJ Open. 2019 Oct 22;9(10):e030023. doi: 10.1136/bmjopen-2019-030023 (PMC6830595; doi:10.1136/bmjopen-2019-030023)
Supplement: Author's manuscript [file bmjopen-2019-030023.draft_revisions.pdf]

BMJ Open

BMJ Open is committed to open peer review. As part of this commitment we make the peer review history of every article we publish publicly available.

When an article is published we post the peer reviewers' comments and the authors' responses online. We also post the versions of the paper that were used during peer review. These are the versions that the peer review comments apply to.

The versions of the paper that follow are the versions that were submitted during the peer review process. They are not the versions of record or the final published versions. They should not be cited or distributed as the published version of this manuscript.

BMJ Open is an open access journal and the full, final, typeset and author-corrected version of record of the manuscript is available on our site with no access controls, subscription charges or pay-per-view fees (http://bmjopen.bmj.com).

If you have any questions on BMJ Open's open peer review process please email info.bmjopen@bmj.com

BMJ Open

Combining cognitive bias modification training (CBM) and transcranial direct current stimulation (tDCS) to treat binge eating disorder: study protocol of a randomised controlled feasibility trial

Journal:	*BMJ Open*
Manuscript ID	bmjopen-2019-030023
Article Type:	Protocol
Date Submitted by the Author:	28-Feb-2019

Complete List of Authors:	Gordon, Gemma; King's College London, Section of Eating Disorders, Department of Psychological Medicine, Institute of Psychiatry, Psychology and Neuroscience,<br>Brockmeyer, Timo; University of Gottingen, Department of Clinical Psychology and Psychotherapy, Institute of Psychology<br>Schmidt, Ulrike; King's College London, Section of Eating Disorders, Department of Psychological Medicine, Institute of Psychiatry, Psychology and Neuroscience<br>Campbell, Iain; King's College London, Section of Eating Disorders, Department of Psychological Medicine, Institute of Psychiatry, Psychology and Neuroscience

Keywords:	Eating disorders < PSYCHIATRY, transcranial direct current stimulation, cognitive bias modification, binge eating, treatment

SCHOLARONE™
Manuscripts

10.02.2019 V1

Combining cognitive bias modification training (CBM) and transcranial direct current stimulation (tDCS) to treat binge eating disorder: study protocol of a randomised controlled feasibility trial

Gemma Gordon[1], Timo Brockmeyer[2], Ulrike Schmidt[1], Iain Campbell[1]

[1]Section of Eating Disorders, Department of Psychological Medicine, Institute of Psychiatry, Psychology and Neuroscience, King's College London, London, United Kingdom

[2]Department of Clinical Psychology and Psychotherapy, Institute of Psychology, University of Goettingen, Goettingen, Germany

Correspondence: Gemma Gordon, Section of Eating Disorders, Department of Psychological Medicine, Institute of Psychiatry, Psychology and Neuroscience, King's College London, London, United Kingdom; E-mail: gemma.gordon@kcl.ac.uk

Key words: cognitive bias modification, transcranial direct current stimulation, eating disorders, binge eating, treatment

Word count (abstract): 290 **Word count (manuscript): 5,908**

For peer review only

ABSTRACT

Introduction: Binge Eating Disorder (BED) is a common mental disorder, closely associated with obesity. Existing treatments are only moderately effective with high relapse rates, necessitating novel interventions. This paper describes the rationale for, and protocol of, a feasibility randomised controlled trial (RCT), evaluating the combination of transcranial Direct Current Stimulation (tDCS) and a computerised cognitive training, namely Approach Bias Modification training (ABM), in patients with Binge Eating Disorder who are overweight or obese. The aim of this trial is to obtain information that will guide decision making and protocol development in relation to a future large-scale RCT of combined tDCS + ABM treatment in this group of patients, and also to assess the preliminary efficacy of this intervention. **Methods and analysis:** 66 participants with DSM-5 diagnosis of BED and a body mass index (BMI) of >25 kg/m^2 will be randomly allocated to one of 3 groups: ABM + real tDCS; ABM + sham tDCS or a control group. Participants in both intervention groups will receive 6 sessions of ABM + real/sham tDCS over 3 weeks; engaging in the ABM task while simultaneously receiving bilateral tDCS to the dorsolateral prefrontal cortex. ABM is based on an implicit learning paradigm in which participants are trained to enact an avoidance behaviour in response to visual food cues. Assessments will be conducted at baseline, post-treatment (3 weeks), and at follow-up (7 weeks post-randomisation). Feasibility outcomes assess recruitment and retention rates, acceptability of random allocation, blinding success (allocation concealment), completion of treatment sessions and research assessments. Other outcomes include eating disorder psychopathology and related neurocognitive outcomes (i.e. delay of gratification and inhibitory control), BMI, other psychopathology (i.e. mood), approach bias towards food, and surrogate endpoints (i.e. food cue reactivity, trait food craving, and food intake).

Ethics and dissemination: This study has been approved by the North West - Liverpool East Research Ethics Committee. Results will be published in peer-reviewed journals.

Trial registration number: ISRCTN35717198

For peer review only

Strengths and limitations of this study

- The ICARUS study is the first randomised controlled feasibility trial of multi-session transcranial Direct Current Stimulation (tDCS) combined with Cognitive Bias Modification training (CBM) for adults with Binge Eating Disorder.
- ICARUS will compare [tDCS + CBM] vs. [sham tDCS + CBM] and a wait-list control group.
- ICARUS is designed to answer questions about the efficacy of the treatments tested.
- Results would need to be replicated in a larger trial before recommendations for tDCS + CBM as a treatment adjunct for patients receiving outpatient treatment for BED can be made.

For peer review only - http://bmjopen.bmj.com/site/about/guidelines.xhtml

INTRODUCTION

Binge Eating Disorder (BED) is the most prevalent eating disorder (ED) worldwide, with 1-3% of the general population meeting diagnostic criteria[1,2]. Binge eating is a core symptom, characterised by consumption of large amounts of food, a sense of loss of control, and significant distress. Nearly 80% of those with lifetime BED have a comorbid psychiatric disorder, such as mood, anxiety, substance use disorders or another ED[2]. Due to the lack of compensatory behaviours (e.g. vomiting, excessive exercising), BED is often accompanied by, or leads to, obesity and associated physical complications[3,4]. In the general population, approximately 30-42% of people with BED are obese[2,5,6]. Around 30% of treatment-seeking obese people[7–9] and up to 47% of bariatric surgery candidates have full or partial BED[1,10,11]. Whilst BED itself has considerable individual and societal costs[12], the combination of BED and obesity is associated with more severe obesity, greater medical and psychiatric comorbidity, greater functional impairment and perinatal complications[12–15]. Treatments for BED and obesity are sub-optimally effective, with cognitive behavioural therapy (CBT) and some medications reducing binge eating and related psychopathology[1], and approximately 50-60% of patients achieving abstinence from bingeing at the end of treatment[18] with some sustained cessation at follow-up[19]. However, drop-out rates in established BED treatments reach 12-34%, and 30%-50% of BED patients relapse in long-term follow-ups[20–22], indicating that a substantial proportion do not maintain binge eating remission. Lisdexamfetamine[23] and topiramate[24] also reduce weight in the short-term but have considerable side effects[25], and their longer term efficacy is uncertain. Thus, there is a need for novel treatment developments.

The aetiology of BED is widely seen as multi-factorial. Emerging neurobiological models emphasise both the role of stress in the onset and maintenance of the disorder[26,27], and the development of addiction-like features; craving, tolerance and binge escalation over time[28,29], impulsivity and compulsivity, alterations in executive function and attention[30] and reward-related decision making[31].

Upon encountering images of high-calorie food, BED patients report enhanced reward sensitivity and exhibit stronger medial orbitofrontal cortex responses compared to healthy controls and participants with bulimia nervosa[32]. In individuals with obesity, who may or may not have BED, activation in the ventral striatum (part of the reward system) has been found to be higher compared to normal-weight controls [33], in tandem with a more pronounced approach bias towards appetising food images [34,35], leading to greater likelihood of consumption. Furthermore, poor reward-related decision making behaviour may be a maintaining factor in obesity[36]. Converging data using different methodologies, such as brain imaging, eye tracking, and behavioural test paradigms [37] have found that patients with BED demonstrate a higher arousal rate in response to food stimuli, a concurrent motor plan to start eating, a higher reward sensitivity, and greater inhibitory deficits as compared to individuals without BED[32,38,39]. Those with obesity and BED (compared to obesity alone) have demonstrated that their attentional bias to food images held higher motivational value[40], and responded more to high calorie food images in sites of cognitive planning of motor movements, driven by emotions, which may reflect impulsive tendencies in the face of a binge-eating trigger. This tendency to approach and consume palatable food items may thus be compounded by a greater sensitivity to reward and a decreased capacity to inhibit action tendencies. This is corroborated by the recent finding that individuals with BED or Bulimia Nervosa

For peer review only - http://bmjopen.bmj.com/site/about/guidelines.xhtml

(BN) show higher food cue reactivity (increased cravings) when exposed to visual food cues compared to healthy controls [41]. Such accumulating evidence of BED as a unique diagnostic group situates it as a distinct phenotype within the obesity spectrum that is characterised by increased impulsivity [42].

Conventional treatments of BED, such as CBT may not be best suited to target highly automatic cognitive processes that occur at an early stage in information processing and that are considered to contribute to food craving and associated maladaptive cognitions/behaviours. Two "brain-directed" treatments may provide an avenue for modifying these processes: Approach Bias Modification training (ABM) and transcranial Direct Current Stimulation (tDCS).

Approach Bias Modification training (ABM) is a form of cognitive bias modification training (CBM) that aims to retrain approach bias tendencies (reach out towards) into avoidance ones (move away from)[43] regarding stimuli such as appetitive cues. Participants are systematically trained to show an avoidance movement in response to illness-related rewarding stimuli (e.g. food or alcohol) on a computer screen. ABM techniques have shown potential in several pilot and large-scale randomised controlled studies to treat alcohol[44] and tobacco[45] addictions, and to reduce consumption of cannabis[46] and unhealthy foods[47,48]. ABM has also yielded promising results in people with high levels of food craving and in bulimic eating disorders, including BED[49,50]. However, mixed results in empirical studies across these domains[51,52] raise methodological issues of ABM studies to date, such as low statistical power and suboptimal choice (or absence) of control groups[53] and administration of single versus multiple training sessions.

Transcranial Direct Current Stimulation (tDCS) is a form of non-invasive brain stimulation (NIBS) that has been used as a treatment adjunct for a range of psychiatric disorders, such as depression, schizophrenia and addictions[54–56]. Preliminary evidence suggests that tDCS and other forms of non-invasive brain stimulation are promising tools to reduce food craving, ED symptoms and body weight in bulimic EDs, including BED, and obesity[57]. Additionally, some studies indicate that NIBS may reduce depression/stress levels and improve reward-based decision making in ED patients[58]. A frequent stimulation target is the dorsolateral prefrontal cortex (dlPFC) which plays a major role in cognitive-inhibition, emotion regulation, and reward processing[58–61]. Although precise mechanisms of action of tDCS have yet to be understood, a key hypothesis in relation to BED is that enhancing dlPFC activity via tDCS alters the reward-cognition balance towards facilitation of cognitive control and suppression of reward-related mechanisms driving food craving/overeating[62].

If given concurrently (i.e. 'online training'), NIBS is reported to boost the effects of cognitive training on the reduction of cognitive biases and the improvement of response inhibition[63]. NIBS may enhance synaptic strength in neuronal pathways activated by cognitive training, amplifying effects of training, and thus cognitive bias modification efficacy [64]. As the effectiveness of tDCS may thus be improved by pairing administration with a cognitive task inducing activity in the target brain region[65–67], such combined treatment interventions have been investigated among alcohol dependent inpatients (ABM and tDCS)[68], and to enhance inhibitory control related to food consumption (Go/No-Go Task and tDCS)[65]. The insignificant findings from these studies warrant commentary that to date, studies that have found positive effects of tDCS have either included obese participants or have had multisession protocols[65,69–71]. As this study incorporates both

For peer review only - http://bmjopen.bmj.com/site/about/guidelines.xhtml

aspects, it is optimally designed to yield significant results.

In light of both the individual and societal burden incurred by the rising prevalence of BED and obesity, research interventions informing treatments that lead to stable and long-lasting remission are of critical importance. This research trial is the first to combine two promising novel intervention strategies in an integrated treatment and will yield important findings to shape future clinical trials. The intervention conditions of this feasibility study will involve 6 sessions of concurrent ABM and real or sham tDCS over 3 weeks, and will assess participant acceptability and dropout rates at this treatment frequency and duration. Additionally, the frequency of participants' ED symptoms and other outcomes related to general psychopathology and neurocognition will be measured before and after the study interventions to assess treatment success. In summary, this proof-of-concept and feasibility study will establish the utility of concurrent ABM + real tDCS in improving clinical outcomes in participants with BED, compared to ABM + sham tDCS, and a wait-list control group.

STUDY AIMS

In line with established recommendations for outcomes of feasibility trials[72], the primary aim is to assess the feasibility of using concurrent ABM + real tDCS compared to concurrent ABM + sham tDCS as an adjunct to treatment as usual (TAU) in this patient population, and acquire key information to inform the development of a large-scale randomised sham-controlled trial (RCT).

The specific objectives of the proposed feasibility study are to:

1. Establish the feasibility of conducting a large-scale RCT of ABM + tDCS in patients with BED by assessing recruitment, attendance, and retention rates;
2. Determine the practicality of administering both ABM and tDCS simultaneously;
3. Determine the best instruments for measuring outcomes in a full trial by examining the quality, completeness, and variability in the data;
4. Estimate the treatment effect sizes and standard deviations for outcome measures to inform the sample size calculation for a large-scale RCT;
5. Evaluate whether the treatment is operating as it is designed by analysing process measures, such as within-session visual analogue scales (VAS) of key ED symptoms;
6. Determine whether patients with BED evaluate concurrent ABM + tDCS as acceptable and credible;
7. Obtain information about patients' willingness to undergo random allocation to ABM paired with either real or sham tDCS administration, or the wait-list control condition.

A secondary aim is to investigate the potential efficacy of concurrent delivery of both forms of treatment on binge eating disorder.

This will involve evaluating if:

1. Concurrent sessions of ABM + real tDCS are superior to ABM + sham tDCS and to wait-list control in terms of frequency of objective binge eating episodes, food cue reactivity, food craving, food intake, eating disorder psychopathology and mood.
2. Concurrent ABM + tDCS is superior to the two other conditions in having an effect on the targeted neurocognitive mechanism (approach bias for high calorie food) and related neurocognitive parameters (i.e. impulsivity, delayed gratification, emotional regulation).

For peer review only - http://bmjopen.bmj.com/site/about/guidelines.xhtml

METHODS AND ANALYSIS

This study protocol has been written according to the SPIRIT statement (Standard Protocol Items for Randomised Trials)[73] and the CONSORT 2010 statement (Consolidated Standards of Reporting Trials)[72].

Study design

The ICARUS trial (Investigating Concurrent Approach Bias Modification Training and Transcranial Direct Current Stimulation in Binge Eating Disorder) is an exploratory randomised controlled feasibility trial with three parallel treatment conditions; ABM + real tDCS, ABM + sham tDCS and wait-list control. All participants across the two intervention groups will receive a treatment protocol of 6 sessions of ABM + real/sham tDCS conducted over 3 weeks. The comparator groups of a wait-list control and ABM + sham tDCS are necessary to evaluate the potential effect of real versus sham tDCS in participants with BED. The wait-list control group will be examined at the same time points to control for the possibility that improvements in the intervention groups are simply due to regression to the mean, spontaneous remission or other non-specific time effects. All participants will continue with TAU for their ED, and thus this selection of comparators is deemed acceptable. Within treatment session measures will involve visual analogues scales evaluating mood, stress and eating disorder symptoms. Assessments will be conducted 3 times during the study; at baseline, post-treatment (week 3), and at follow-up (week 7).

Participants

Inclusion criteria entail: (1) male and female community-dwelling adults (aged 18-70); (2) overweight or obese according to WHO criteria (BMI>25 kg/m^2)[74]; (3) a diagnosis of full-syndrome or sub-threshold Binge Eating Disorder according to the DSM-5[75]L; (4) fluency in English; (5) normal or corrected to normal vision .
Exclusion criteria entail: (1) all known contraindications to tDCS[76]; (2) pregnancy; (3) a current significant/unstable medical or psychiatric disorder needing acute treatment in its own right; (4) a lifetime diagnosis of substance dependence, psychosis, bipolar disorder or borderline personality disorder; (5) taking psychotropic medication other than a stable dosage of selective serotonin reuptake inhibitors (SSRI) for at least 14 days prior to study enrolment; (6) allergies to any of the foods presented in the study; (7) smoking >10 cigarettes per day; (8) drinking >3-4 units (men) or 2-3 units (women) of alcohol per day. In line with the CONSORT guidelines[77,78], we will record the number and reasons for any participants we must exclude, or any who decline consent or withdraw from the study.

Sample size

As ICARUS is a feasibility study, an a priori sample size calculation is not necessary. Rather, its aim is to provide effect sizes on which future large-scale studies can be powered. Total sample sizes of n=24 to n=50 have been recommended for feasibility trials with a primary outcome measured on a continuous scale, mainly because estimates of the standard deviation for normally distributed variables tend to stabilise around this size[79,80]. We have chosen a target end sample size of n=60, (i.e. exceeds the upper end recommended for feasibility trials). However, assuming the attrition to follow-up rate is a = 0.10 (as found in previous eating disorder trials [81,82]) and applying an attrition correction factor of 1/(1-a), we will recruit an actual sample size of 66, i.e., 22 participants per group.

For peer review only - http://bmjopen.bmj.com/site/about/guidelines.xhtml

Randomisation

After the baseline assessment, participants will be allocated to one of three conditions at random to receive 6 sessions of either concurrent ABM + real tDCS or ABM + sham tDCS, or no intervention in the wait-list control condition. Participants in the wait-list control group will be offered the opportunity to receive ABM + real tDCS after the end of the follow-up. Participants will be individually randomised on a 1:1:1 ratio to the intervention or control groups in equal numbers. The generation and implementation of the randomisation sequence will be conducted independently from the trial team through a randomisation administrator who is not involved in any recruitment or research activity related to the ICARUS study. Online randomisation software (Sealed Envelope, London, UK) will be used for this purpose. Upon participant enrolment, the researcher will contact the randomisation administrator, who will inform this researcher in charge of carrying out the intervention of the participant's allocation via phone or email.

Blinding

Double blinding is implemented only for the intervention group cohorts of the trial. The research assessor will remain blind to each participant’s tDCS assignment within the two intervention conditions until after the participant has completed the follow-up assessment. This double blinding protocol will be ensured via administration of the tDCS (NeuroConn DC-STIMULATOR PLUS) using “study mode”. This involves a five-digit numerical code unique to each patient will be inputted into the device prior to the participant’s testing session, that will initialise either sham or real (active) stimulation. The tDCS administrator and participants will remain blind to tDCS stimulation type throughout the study. Set-up of the randomisation codes and programming of the tDCS device will be performed by an investigator not involved in the trial. To assess blinding success, each participant and the researcher will be asked to guess the treatment allocation at the end of the 6 treatment sessions and to indicate how certain they are of this guess. The study group allocation will be revealed to the participant after their follow-up assessment. In the event of a reported change in a participant’s medication, or a new clinical diagnosis made during their study participation, the early unblinding of study condition for an intervention group participant will be permissible. The trial database will be maintained ‘blind’ until the point of study data analyses.

Recruitment

The study will take place at the Institute of Psychiatry, Psychology and Neuroscience (IoPPN), King’s College London (KCL), UK. Participants will be recruited from the Eating Disorders Service at the South London and Maudsley NHS Foundation Trust, from the KCL research recruitment webpage and social media account, and via posters placed on notice boards on KCL campuses. Participants who have previously taken part in research at the KCL Eating Disorders Unit and who have consented to be informed of future studies may also be contacted. The ICARUS study will also be advertised on the Beat (National Eating Disorders Association) website, callforparticipants.com and www.mqmentalhealth.org. Potential participants will receive written and verbal study information and will be screened for eligibility. Eligible participants will provide informed written consent for study participation as a prerequisite for enrolment (See Appendices B and C).

Procedure

For peer review only - http://bmjopen.bmj.com/site/about/guidelines.xhtml

Figure 1 illustrates the timeline of the study procedures. All participants will partake in assessments at each of the three measurement points; baseline, post-treatment, and follow-up. Each assessment will comprise of an in-person study visit with tasks and measures, and online/hardcopy questionnaires and scales to be completed at home by the participant within 36 hours following the study visit. Table 1 details the tasks and measures allocated to each assessment and training visit. After the baseline assessment, participants are randomised to one of 3 groups; (1) ABM + real tDCS, (2) ABM + sham tDCS, or a waiting list control group (CG). Participants allocated to an intervention group will be offered 6 sessions of ABM + real/sham tDCS across 3 weeks. They may also receive TAU e.g. if they are currently engaged in outpatient treatment for their ED. The control group will not receive any study intervention, and may continue TAU during this 3 week period. The post-treatment assessment will be conducted on all participants after the 6th (final) session of ABM + real/sham tDCS for the intervention groups, and 3 weeks after the baseline assessment for the control group. The follow-up assessment will be conducted 28 days after the end of treatment, i.e. 7 weeks post-randomisation. A follow-up period is included because, if the effects of the intervention result from increased neuroplasticity, behavioural changes may need time to emerge. Assessing the longevity of favourable clinical outcomes beyond the treatment period is also relevant to the objectives of this feasibility study. The researcher conducting the assessment and testing sessions will remain blind to the study condition of intervention group participants until the follow-up has been completed.

Outcome assessment

Measures of feasibility, safety and adherence will be collected throughout the study. Outcomes related to ED symptoms, general psychopathology and neurocognition will be measured before and after the study intervention to assess treatment success. Each assessment session will be split into an in-person visit and at-home component to accommodate time constraints and minimise disruption to task performance due to participant fatigue. The in-person assessment measures will take approximately 75 minutes to complete, and the online questionnaires will take approximately 45 minutes.

Outcome Measures

Feasibility outcomes

As this is a feasibility study, an extensive range of outcome measures are included to help determine which are most sensitive to detecting a treatment effect. This will enable us to determine primary outcome(s) for a future large-scale RCT. However, based on previous research[49], the Eating Disorder Examination Questionnaire (EDE-Q) is anticipated to be a key outcome measure.

Intervention/service related outcomes

Feasibility outcomes include recruitment, attendance and retention rates, and acceptability of treatment by participants. Patients' acceptance of study interventions will be assessed by measuring treatment dropout rates and via the treatment tolerance and acceptability questionnaires. An interview assessment of treatment experience will be conducted with 20 participants after the follow-up is completed. 10 participants from each intervention group will be invited to provide

For peer review only - http://bmjopen.bmj.com/site/about/guidelines.xhtml

feedback on their initial expectations and experiences of the ABM + real/sham tDCS treatments, perceived strengths and weaknesses of the treatment they received, and suggestions for improvements in procedures. Interviews will be recorded, transcribed and analysed using thematic analysis. This will allow future studies to consider patients' feedback in the development of research and clinical protocols of concurrent ABM and tDCS.

Clinical outcomes

Eating disorder and related psychopathology
(a) Eating Disorder Examination (EDE-Q)[83]: The EDE-Q is a widely used measure of eating-disordered behaviour and is widely regarded as the instrument of choice for the assessment of EDs. This will be administered at baseline, post-assessment and follow-up.

b) Body Mass Index (BMI (kg/m2)): This assessment of body composition provides accurate estimates of body fat percentages in adults, where sex and age are factored into the analysis measuring height and weight[84]. To calculate BMI, height and weight measurements will be obtained by the researcher at baseline, post-assessment and follow-up.

(c) *Approach bias assessment tasks*
To identify the most sensitive method of assessing change in approach bias towards high-calorie food items, two different computerised measures of approach bias will be used. In the Food Approach-Avoidance Task (F-AAT)[85] participants are shown colour photographs of high-calorie, palatable foods such as chocolate, cake, and pizza, and non-food household and office items such as sponges and stationary on a computer-screen[86]. They are instructed to approach and avoid these stimuli by moving a joystick toward themselves (approach) or away from themselves (avoidance). In the Stimulus Response Compatibility Task (SRC)[87], participants perform a symbolic movement by making a manikin image walk toward (approach) or away from stimuli (avoidance). See Appendix A for more detailed information. Both of these tasks will be administered at baseline, post-assessment and follow-up.

(d) Food choice attitudes/behaviour
The Food Choice Task[88–90] is a computer-based paradigm that measures responses to images of foods to assess food attitudes and characteristics of eating behaviour. Participants rate images of food on a computer screen according to healthiness as well as tastiness. Based on these ratings they are then offered a choice between a food that they consider "neutral" and a series of other foods. See Appendix A for more detailed information. This task will be performed at baseline and post-assessment.

(e) *Food craving after cue exposure task*
The Food Challenge Task (FCT)[41] will be used to examine cue-induced food craving. In this task, participants rate their state food craving using the Food Cravings Questionnaire State Version[91,92] before and after being presented with a video on a computer screen of foods shown to be highly appetising[93]. See Appendix A for more detailed information. This task will be performed at baseline and post-assessment.

(f) *Trait food craving*

For peer review only - http://bmjopen.bmj.com/site/about/guidelines.xhtml

Three questionnaires will be used to comprehensively assess mechanisms implicated in trait food craving. The Food Cravings Questionnaire Trait Version - reduced (FCQ-T-r)[94] is a 15 items only reduced version of a self-report questionnaire that measures trait levels of craving for food. The 21-item Power of Food Scale (PFS)[95] scale assesses the psychological influence of the mere presence or availability of food. It measures appetite for, rather than consumption of, palatable foods, at three levels of food proximity (food available, food present, and food tasted). The Yale Food Addiction Scale Version 2.0 (YFAS 2.0)[96] reflects the current diagnostic understanding of addiction to further investigate the potential role of an addictive process in problematic eating behaviour[75,97–101]. See Appendix A for more detailed information. Each of these scales will be administered at baseline, post-assessment and follow-up.

(g) *Food intake in a bogus taste test[102]*
Participants will be instructed to rate and optionally consume highly palatable high-calorie food items presented in 3 bowls. See Appendix A for more detailed information. This task will be performed at baseline and post-assessment.

(h) *Increased preference for delayed rewards*
The Delay Discounting Task with Money and Food[103] examines whether small amounts of food would be discounted more steeply than money, as occurs with larger amounts. See Appendix A for more detailed information. This will be administered at baseline, post-assessment and follow-up.

(i) *Inhibitory control*
The cued Go/No-Go computer task is a classic test of executive function, requiring effortful response inhibition, and measures impulse control by the ability to inhibit instigated, prepotent responses. A food specific go/no-go task[104] measures impulsivity and response inhibition with respect to food and nonfood items. The Stop Signal Task (SST)[105]measures inhibitory control. Participants are required to engage in a computer task but withhold their response in the presence of a stop signal. An adaptation of the food version of the SST[106] will facilitate a comparison of responses between food and non-food categories. See Appendix A for more detailed information. Both of these tasks will be performed at baseline, post-assessment and follow-up.

Mood and emotion regulation

The Emotion Regulation Questionnaire (ERQ)[107] is designed to measure respondents' tendency to regulate their emotions regarding cognitive reappraisal and expressive suppression. The Positive and Negative Affect Schedule (PANAS)[108] measures the degree of positive or negative affect experienced "right now" in the current study. The Depression, Anxiety and Stress Scale (DASS-21)[109] evaluates mood, anxiety and stress levels over the previous week. See Appendix A for more detailed information. All of these measures will be administered at baseline, post-assessment and follow-up.

Within session measures

Within each training session, i.e. immediately before and after the ABM + real/sham tDCS procedure the researcher will administer paper-based Visual Analogue Scales (VAS) assessing current hunger,

For peer review only

feeling of fullness, urge to eat, urge to binge eat, feeling low, level of tension, level of stress, level of anxiety, and any discomfort due to tDCS and ABM in the training session. See Appendix A for more detailed information.

Intervention

In both intervention groups participants will receive six sessions of concurrent ABM and real or sham tDCS which will be delivered twice a week for three weeks. A researcher trained in tDCS administration will deliver the training sessions.

Rationale for number of sessions:

Treatment parameters for interventions of ABM and tDCS separately in psychiatric disorder research have not yet been standardised and vary from 1-12 sessions across a timeframe of days to multiple weeks. Mixed results regarding optimal frequency of ABM sessions and related forms of cognitive bias modification has been reported[110]. A maximum accumulative effect of modification efficacy at 6 sessions has been found for approach bias modification for alcohol dependence[44]. While there is a similar paucity of specifications for treatment parameters within tDCS, multisession NIBS interventions are significantly more effective at reducing cravings and strengthening the ability to refrain from food consumption than single session protocols in eating disorders and obesity[111]. As a single session of tDCS on patients with BED was found to reduce craving and caloric intake[59], it was hypothesised that repeated administration of tDCS would enhance this effect and may decrease binge eating frequency.

Within session safety procedures

The participant's blood pressure and heartrate will be taken by the researcher immediately before and after the session. While the participant is comfortably seated, the tDCS and ABM will be administered at the same time, i.e. participants will engage in ABM training whilst receiving tDCS. Each session will last 20 minutes. The ABM training will start 5 minutes after the start of the brain stimulation. ABM training will take place over 10 minutes and tDCS will then continue for a further 5 minutes. Participants will be reminded that they have the option to withdraw immediately and terminate their participation in the study if they experience discomfort during tDCS administration, or if they wish to withdraw for any reason that they may or may not wish to disclose.

Approach bias modification training

The ABM programme will use an implicit learning paradigm, based on a modified version of the Food Approach/Avoidance Task (AAT)[85,112–114]. In this task, participants are shown pictures of food and control (i.e. neutral office) items. They are required to pull (pictures grow bigger) or push (pictures grow smaller) a joystick in response to the outer frame of the picture (round vs. rectangular), irrespective of the picture content. The training version of the Food-AAT utilises an implicit learning paradigm by presenting all food pictures in the "push" (i.e. avoid) format. The study procedure for ABM administration is aligned with previous research[50,115].

Transcranial direct current stimulation

TDCS (both real and sham) will be delivered using a NeuroConn® DC-STIMULATOR PLUS device at a constant current of 2 mA (with a 10-second fade in/out) using two 25cm² surface sponge electrodes

For peer review only - http://bmjopen.bmj.com/site/about/guidelines.xhtml

soaked in a sterile saline solution (0.9% sodium chloride). The anode will be placed over the right dorsolateral prefrontal cortex (dlPFC) and the cathode over the left dlPFC. This montage has been used in sham-controlled studies on food craving, bulimia nervosa and BED[59]. The stimulation site will be located using the Beam F3 calculation method, which is based on the International 10-20 system. TDCS can occasionally result in mild discomfort during administration (i.e., tingling or itching sensation, a slightly metallic taste, occasional redness at the site of the electrodes). Fatigue, headache, nausea and insomnia have been reported as potential adverse reactions[116]. Participants who are at-risk for adverse effects[76] will be excluded from the study at the screening stage.

Data analysis

Data will be analysed with the Statistical Package of Social Sciences (SPSS). Feasibility outcome data will be analysed with appropriate summary statistics. To determine quality, completeness, and variability of the clinical outcome data, descriptive statistical analyses and graphical methods will be used. Intent-to-treat analyses will be performed. The size of the treatment effect on each outcome measure will be the difference in outcome data between those in the two treatment conditions and control condition at post-assessment and follow-up. Group differences will be estimated using linear mixed effects regression models, controlling for the baseline level of the outcome. The goal here is not to determine significant group differences but to establish a suitably precise effect size for the primary outcome at the post treatment assessment. This estimate will be used to guide the sample size of a future efficacy trial. Correlational analyses may be computed to analyse relationships between outcome variables and influences of potential covariates such as gender, age, BMI or comorbidities. Outcome data already obtained for participants who discontinue or deviate from the intervention protocol will be kept and analysed.

PATIENT AND PUBLIC INVOLVEMENT

Patients and/or public were not involved in the study design process, however we will obtain 20 intervention participants' qualitative views on their treatment experience in this study to inform future clinical trials.

ETHICS AND DISSEMINATION

Data management and data monitoring

Participant data will be anonymised and all anonymised data will be stored electronically on a password protected computer at the IoPPN. All trial data will be stored in line with the General Data Protection Regulation (GDPR) 2018. Hard copies of participant-related data (i.e. GP letters) will be kept in locked cabinets at the IoPPN, King's College London. The final trial data set will not be accessed by anyone other than members of the research team.

Data will be stored on manual files, university and laptop computers. There will be no personal data stored on laptop computers. Confidentiality and anonymity of all personal data will be retained throughout the entire study. Manual files will be securely locked in a lockable filing cabinet, and all electronic files will be password protected. Identifying information will be removed from the data, stored separately and replaced with a numeric identification code. All participants will be allocated a numeric code, which will be used to identify their data. The master list of names which correspond

For peer review only - http://bmjopen.bmj.com/site/about/guidelines.xhtml

to each participant's numeric identification code will be stored electronically and will be password protected. This information will only be accessible to key researchers involved in the study.

The online component of the assessment will use Online Surveys software (formerly BOS). King's College London uses this software for large scale surveys, and it is fully compliant with UK data protection laws. Participants will be emailed the link after the in-person component of each assessment session, and instructed to complete the second online component of the assessment within 36 hours. Participants will also have the option to receive and complete a hard copy version of the questionnaires with a stamped addressed envelope to post back to the study researcher at the IoPPN.

It is intended that the results of this feasibility study will be reported and disseminated at national and international conferences. Research findings may also be disseminated through internal newsletters and publications in collaboration with Beat, the UK's largest eating disorder charity.

Owing to the size and nature of this small-scale feasibility study, a data monitoring committee was not deemed to be required. There are no scheduled interim analyses and this trial may be prematurely discontinued by the Chief Investigator on the basis of new safety information.

Ethics and safety aspects

This study has been approved by the North West - Liverpool East Research Ethics Committee. This trial will be conducted in compliance with the study protocol, the Declaration of Helsinki, the principles of good clinical practice (ICH-E6 guideline), the ICH-E8 guideline and the principles of GCP and in accordance with all applicable regulatory requirements including but not limited to the UK policy framework for health and social care research. The ICARUS trial is registered with the International Standard Randomised Controlled Trial Number registry (number ISRCTN35717198). All participants will be asked by the study researcher to provide written informed consent prior to enrolment. Participants who are at-risk for adverse side effects[76]will be excluded from the study at the screening stage (i.e. such as those with pregnancy or epilepsy). Current safety parameters of tDCS administration regarding voltage amplitude and duration of brain stimulation sessions will be adhered to. Participants have the option to withdraw immediately and terminate their participation in the study if they experience discomfort during tDCS administration, or if they wish to terminate their participation for any other reason that they may or may not wish to disclose. After each training session, participants will complete the tolerance, discomfort and side effects questionnaire to report any adverse effects of the intervention training session. The researcher will record this description of any reported adverse effects, and record the severity and duration of symptoms and how the adverse effect was managed at the following training session. If a participant reports a new clinical diagnosis or change in medication during their involvement in the study, a decision regarding their continued participation in the study will be made by the research team and withdrawal of the participant may be deemed necessary. Standard King's College London insurance and NHS indemnity arrangements apply to this study. To promote study adherence, upon completion of the follow-up assessment, each participant will be reimbursed for their time, efforts and travel (£60 for assessments and up to £60 for travel expenses). Additionally, participants in the wait-list control group will be offered the opportunity to receive 6 sessions of ABM + tDCS after the follow-up.

For peer review only - http://bmjopen.bmj.com/site/about/guidelines.xhtml

DISCUSSION

The ICARUS study represents the first feasibility study that aims to exploit a synergistic therapeutic effect by combining two brain-directed interventions in a single treatment intervention for BED. The rising clinical need of individuals with BED is currently met with few available psychological and neuropharmacological treatment options[117]. Therefore, such research advancing the identification and validation of novel therapies is greatly warranted.

This paper delineates the protocol for a feasibility trial which will inform future studies (i.e., provide effect sizes for a large RCT) and contribute to the extant research advocating brain-directed interventions for BED. The protocol aligns with current parameters of tDCS administration used to treat BED and BN[58,59] and utilises a multitudes of measures to identify the most appropriate and sensitive tools to detect treatment induced changes across pathological and neurocognitive domains.

Pragmatic concerns related to the recruitment process entail ensuring a sufficient and consistent rate of participant enrollment to meet the target sample number within the allocated timeframe. Additionally, drop-out rates for CBT treatment among a BED cohort are moderately high (17–30%)[118], thus study adherence will need to be monitored, with a revision of incentives/ reimbursement if necessary. Participants who were randomly allocated to the wait list control may avail themselves of 6 sessions of ABM + real tDCS after they have completed the study, which may promote recruitment and participant retention. Documenting the management of these issues will help to inform the development of a future large-scale RCT of this combined treatment adjunct for BED.

To conclude, investigating novel treatments for BED is an imperative issue. Combining ABM with tDCS is the strategic amalgamation of two techniques that have already demonstrated therapeutic efficacy in their own right. This feasibility RCT will be the first to systematically assess the acceptability and efficacy of a noninvasive, safe and potentially effective treatment adjunct to other therapies, which will enhance the ability of healthcare services to provide optimal care to patients with BED.

Trial progress

Recruitment will commence in February 2018 and data collection is expected to be complete (including follow-up assessments) by April 2020. Any substantial protocol amendments will be communicated to investigators via email and to other parties as required. Amendments to the study protocol will be reported in publications reporting the study outcomes.

Author affiliations

[1] Section of Eating Disorders, Department of Psychological Medicine, Institute of Psychiatry, Psychology & Neuroscience, King's College London, London, UK

[2] Department of Clinical Psychology and Psychotherapy, Institute of Psychology, University of Goettingen, Goettingen, Germany

Acknowledgements Not applicable

For peer review only - http://bmjopen.bmj.com/site/about/guidelines.xhtml

Contributors GG, IC, US and TB were each involved in the study conception and design. GG drafted the study protocol and IC, US and TB reviewed the paper and informed subsequent drafts. GG, IC, US and TB approved the final protocol paper.

Authorship policy No professional writers were involved in this study protocol, nor will be involved in the study report write-up.

Funding The project is supported by the NIHR Biomedical Research Centre (BRC) at South London and Maudsley NHS Foundation Trust (SLaM) and King's College London (KCL). Gemma Gordon is supported by a BRC PhD studentship and Ulrike Schmidt is supported by an NIHR Senior Investigator Award and receives salary support from the NIHR Mental Health BRC at SLaM NHS Foundation Trust and KCL. The funder was not involved in the study design and writing of this trial protocol paper, and will not be involved in the collection and analysis of data, nor the writing of the study report. The funders will not have ultimate authority over these activities.

Disclaimer The views expressed are those of the author(s) and not necessarily those of the NHS, the NIHR or the Department of Health.

Competing interests None declared

Patient consent Informed consent will be obtained by the researcher.

Ethics approval Ethical approval was given by the North West - Liverpool East Research Ethics Committee (ref: 18/NW/0648).

Provenance and peer review Not commissioned; externally peer reviewed.

Data sharing statement Not applicable

Open access This is an open access article distributed in accordance with the terms of the Creative Commons Attribution (CC BY 4.0) license, which permits others to distribute, remix, adapt and build upon this work, for commercial use, provided the original work is properly cited. See: http://creativecommons.org/ licenses/by/4.0/

© Article author(s) (or their employer(s) unless otherwise stated in the text of the article) 2018. All rights reserved. No commercial use is permitted unless otherwise expressly granted.

For peer review only

REFERENCES

1 McCuen-Wurst C, Ruggieri M, Allison KC. Disordered eating and obesity: associations between binge-eating disorder, night-eating syndrome, and weight-related comorbidities: Disordered eating and obesity. *Annals of the New York Academy of Sciences* 2018;**1411**:96–105. doi:10.1111/nyas.13467

2 Kessler RC, Berglund PA, Chiu WT, *et al.* The Prevalence and Correlates of Binge Eating Disorder in the World Health Organization World Mental Health Surveys. *Biological Psychiatry* 2013;**73**:904–14. doi:10.1016/j.biopsych.2012.11.020

3 de Zwaan M. Binge eating disorder and obesity. *Int J Obes Relat Metab Disord* 2001;**25 Suppl 1**:S51-55. doi:10.1038/sj.ijo.0801699

4 Hutson PH, Balodis IM, Potenza MN. Binge-eating disorder: Clinical and therapeutic advances. *Pharmacol Ther* 2018;**182**:15–27. doi:10.1016/j.pharmthera.2017.08.002

5 Cossrow N, Pawaskar M, Witt EA, *et al.* Estimating the Prevalence of Binge Eating Disorder in a Community Sample From the United States: Comparing DSM-IV-TR and DSM-5 Criteria. *J Clin Psychiatry* 2016;**77**:e968-974. doi:10.4088/JCP.15m10059

6 Kornstein SG, Kunovac JL, Herman BK, *et al.* Recognizing Binge-Eating Disorder in the Clinical Setting: A Review of the Literature. *Prim Care Companion CNS Disord* 2016;**18**. doi:10.4088/PCC.15r01905

7 Spitzer RL, Devlin M, Walsh BT, *et al.* Binge eating disorder: A multisite field trial of the diagnostic criteria. *International Journal of Eating Disorders* 1992;**11**:191–203. doi:10.1002/1098-108X(199204)11:3<191::AID-EAT2260110302>3.0.CO;2-S

8 Spitzer RL, Yanovski S, Wadden T, *et al.* Binge eating disorder: its further validation in a multisite study. *Int J Eat Disord* 1993;**13**:137–53.

9 Grucza RA, Przybeck TR, Cloninger CR. Prevalence and correlates of binge eating disorder in a community sample. *Comprehensive Psychiatry* 2007;**48**:124–31. doi:10.1016/j.comppsych.2006.08.002

10 Allison KC, Wadden TA, Sarwer DB, *et al.* Night eating syndrome and binge eating disorder among persons seeking bariatric surgery: prevalence and related features. *Obesity (Silver Spring)* 2006;**14 Suppl 2**:77S-82S. doi:10.1038/oby.2006.286

11 Adami GF, Gandolfo P, Bauer B, *et al.* Binge eating in massively obese patients undergoing bariatric surgery. *Int J Eat Disord* 1995;**17**:45–50.

12 Pawaskar M, Witt EA, Supina D, *et al.* Impact of binge eating disorder on functional impairment and work productivity in an adult community sample in the United States. *International Journal of Clinical Practice* 2017;**71**:e12970. doi:10.1111/ijcp.12970

13 Brown KL, LaRose JG, Mezuk B. The relationship between body mass index, binge eating disorder and suicidality. *BMC Psychiatry* 2018;**18**. doi:10.1186/s12888-018-1766-z

14 Watson HJ, Zerwas S, Torgersen L, *et al.* Maternal Eating Disorders and Perinatal Outcomes: A Three-Generation Study in the Norwegian Mother and Child Cohort Study. *J Abnorm Psychol* 2017;**126**:552–64. doi:10.1037/abn0000241

For peer review only - http://bmjopen.bmj.com/site/about/guidelines.xhtml

15 Raevuori A, Suokas J, Haukka J, *et al.* Highly increased risk of type 2 diabetes in patients with binge eating disorder and bulimia nervosa. *Int J Eat Disord* 2015;**48**:555–62. doi:10.1002/eat.22334

16 Palavras MA, Hay P, Filho CADS, *et al.* The Efficacy of Psychological Therapies in Reducing Weight and Binge Eating in People with Bulimia Nervosa and Binge Eating Disorder Who Are Overweight or Obese-A Critical Synthesis and Meta-Analyses. *Nutrients* 2017;**9**. doi:10.3390/nu9030299

17 Brownley KA, Berkman ND, Peat CM, *et al.* Binge-Eating Disorder in Adults: A Systematic Review and Meta-analysis. *Ann Intern Med* 2016;**165**:409–20. doi:10.7326/M15-2455

18 de Zwaan M, Herpertz S, Zipfel S, *et al.* Effect of Internet-Based Guided Self-help vs Individual Face-to-Face Treatment on Full or Subsyndromal Binge Eating Disorder in Overweight or Obese Patients: The INTERBED Randomized Clinical Trial. *JAMA Psychiatry* 2017;**74**:987–95. doi:10.1001/jamapsychiatry.2017.2150

19 Hilbert A, Bishop ME, Stein RI, *et al.* Long-term efficacy of psychological treatments for binge eating disorder. *Br J Psychiatry* 2012;**200**:232–7. doi:10.1192/bjp.bp.110.089664

20 Brownley KA, Berkman ND, Sedway JA, *et al.* Binge eating disorder treatment: a systematic review of randomized controlled trials. *Int J Eat Disord* 2007;**40**:337–48. doi:10.1002/eat.20370

21 Vocks S, Tuschen-Caffier B, Pietrowsky R, *et al.* Meta-analysis of the effectiveness of psychological and pharmacological treatments for binge eating disorder. *Int J Eat Disord* 2010;**43**:205–17. doi:10.1002/eat.20696

22 Wilson GT, Wilfley DE, Agras WS, *et al.* Psychological treatments of binge eating disorder. *Arch Gen Psychiatry* 2010;**67**:94–101. doi:10.1001/archgenpsychiatry.2009.170

23 McElroy SL, Hudson J, Ferreira-Cornwell MC, *et al.* Lisdexamfetamine Dimesylate for Adults with Moderate to Severe Binge Eating Disorder: Results of Two Pivotal Phase 3 Randomized Controlled Trials. *Neuropsychopharmacology* 2016;**41**:1251–60. doi:10.1038/npp.2015.275

24 Guerdjikova AI, Fitch A, McElroy SL. Successful Treatment of Binge Eating Disorder With Combination Phentermine/Topiramate Extended Release. *Prim Care Companion CNS Disord* 2015;**17**. doi:10.4088/PCC.14l01708

25 Arif H, Buchsbaum R, Weintraub D, *et al.* Patient-reported cognitive side effects of antiepileptic drugs: Predictors and comparison of all commonly used antiepileptic drugs. *Epilepsy & Behavior* 2009;**14**:202–9. doi:10.1016/j.yebeh.2008.10.017

26 Razzoli M, Pearson C, Crow S, *et al.* Stress, overeating, and obesity: Insights from human studies and preclinical models. *Neurosci Biobehav Rev* 2017;**76**:154–62. doi:10.1016/j.neubiorev.2017.01.026

27 Vannucci A, Nelson EE, Bongiorno DM, *et al.* Behavioral and neurodevelopmental precursors to binge-type eating disorders: support for the role of negative valence systems. *Psychological Medicine* 2015;**45**:2921–36. doi:10.1017/S003329171500104X

28 Smith DG, Robbins TW. The neurobiological underpinnings of obesity and binge eating: a rationale for adopting the food addiction model. *Biol Psychiatry* 2013;**73**:804–10. doi:10.1016/j.biopsych.2012.08.026

For peer review only - http://bmjopen.bmj.com/site/about/guidelines.xhtml

29 Devlin M. Binge Eating Disorder. In: *In K.D Brownell & B.T. Walsh (Eds.), Eating Disorders and Obesity, A Comprehemsive Handbook*. New York: : The Guildford Press 2017. 192–7.

30 Kessler RM, Hutson PH, Herman BK, *et al.* The neurobiological basis of binge-eating disorder. *Neuroscience & Biobehavioral Reviews* 2016;**63**:223–38. doi:10.1016/j.neubiorev.2016.01.013

31 Wu M, Brockmeyer T, Hartmann M, *et al.* Reward-related decision making in eating and weight disorders: A systematic review and meta-analysis of the evidence from neuropsychological studies. *Neurosci Biobehav Rev* 2016;**61**:177–96. doi:10.1016/j.neubiorev.2015.11.017

32 Schienle A, Schäfer A, Hermann A, *et al.* Binge-eating disorder: reward sensitivity and brain activation to images of food. *Biol Psychiatry* 2009;**65**:654–61. doi:10.1016/j.biopsych.2008.09.028

33 Stice E, Spoor S, Ng J, *et al.* Relation of obesity to consummatory and anticipatory food reward. *Physiol Behav* 2009;**97**:551–60. doi:10.1016/j.physbeh.2009.03.020

34 Brignell C, Griffiths T, Bradley BP, *et al.* Attentional and approach biases for pictorial food cues. Influence of external eating. *Appetite* 2009;**52**:299–306. doi:10.1016/j.appet.2008.10.007

35 Mehl N, Mueller-Wieland L, Mathar D, *et al.* Retraining automatic action tendencies in obesity. *Physiol Behav* 2018;**192**:50–8. doi:10.1016/j.physbeh.2018.03.031

36 Brockmeyer T, Simon JJ, Becker A, *et al.* Reward-related decision making and long-term weight loss maintenance. *Physiol Behav* 2017;**181**:69–74. doi:10.1016/j.physbeh.2017.09.008

37 Paslakis G, Kühn S, Grunert S, *et al.* Explicit and Implicit Approach vs. Avoidance Tendencies towards High vs. Low Calorie Food Cues in Patients with Obesity and Active Binge Eating Disorder. *Nutrients* 2017;**9**. doi:10.3390/nu9101068

38 Schag K, Teufel M, Junne F, *et al.* Impulsivity in binge eating disorder: food cues elicit increased reward responses and disinhibition. *PLoS ONE* 2013;**8**:e76542. doi:10.1371/journal.pone.0076542

39 Svaldi J, Naumann E, Trentowska M, *et al.* General and food-specific inhibitory deficits in binge eating disorder: Behavioral Inhibition in Bed. *International Journal of Eating Disorders* 2014;**47**:534–42. doi:10.1002/eat.22260

40 Aviram-Friedman R, Astbury N, Ochner CN, *et al.* Neurobiological evidence for attention bias to food, emotional dysregulation, disinhibition and deficient somatosensory awareness in obesity with binge eating disorder. *Physiol Behav* 2018;**184**:122–8. doi:10.1016/j.physbeh.2017.11.003

41 Meule A, Küppers C, Harms L, *et al.* Food cue-induced craving in individuals with bulimia nervosa and binge-eating disorder. *PLoS ONE* 2018;**13**:e0204151. doi:10.1371/journal.pone.0204151

42 Giel KE, Teufel M, Junne F, *et al.* Food-Related Impulsivity in Obesity and Binge Eating Disorder-A Systematic Update of the Evidence. *Nutrients* 2017;**9**. doi:10.3390/nu9111170

43 Kakoschke N, Kemps E, Tiggemann M. Approach bias modification training and consumption: A review of the literature. *Addict Behav* 2017;**64**:21–8. doi:10.1016/j.addbeh.2016.08.007

For peer review only - http://bmjopen.bmj.com/site/about/guidelines.xhtml

44 Eberl C, Wiers RW, Pawelczack S, *et al.* Implementation of approach bias re-training in alcoholism-how many sessions are needed? *Alcohol Clin Exp Res* 2014;**38**:587–94. doi:10.1111/acer.12281

45 Wittekind CE, Feist A, Schneider BC, *et al.* The approach-avoidance task as an online intervention in cigarette smoking: a pilot study. *J Behav Ther Exp Psychiatry* 2015;**46**:115–20. doi:10.1016/j.jbtep.2014.08.006

46 Sherman BJ, Baker NL, Squeglia LM, *et al.* Approach bias modification for cannabis use disorder: A proof-of-principle study. *Journal of Substance Abuse Treatment* 2018;**87**:16–22. doi:10.1016/j.jsat.2018.01.012

47 Schumacher SE, Kemps E, Tiggemann M. Bias modification training can alter approach bias and chocolate consumption. *Appetite* 2016;**96**:219–24. doi:10.1016/j.appet.2015.09.014

48 Warschburger P, Gmeiner M, Morawietz M, *et al.* Evaluation of an approach-avoidance training intervention for children and adolescents with obesity: A randomized placebo-controlled prospective trial. *Eur Eat Disord Rev* 2018;**26**:472–82. doi:10.1002/erv.2607

49 Brockmeyer T, Hahn C, Reetz C, *et al.* Approach Bias Modification in Food Craving-A Proof-of-Concept Study. *Eur Eat Disord Rev* 2015;**23**:352–60. doi:10.1002/erv.2382

50 Brockmeyer T, Friederich H-C, Küppers C, *et al.* Approach bias modification training in bulimia nervosa and binge-eating disorder: A pilot randomized controlled trial. *Int J Eat Disord* Published Online First: 28 January 2019. doi:10.1002/eat.23024

51 Becker D, Jostmann NB, Wiers RW, *et al.* Approach avoidance training in the eating domain: testing the effectiveness across three single session studies. *Appetite* 2015;**85**:58–65. doi:10.1016/j.appet.2014.11.017

52 Lindgren KP, Wiers RW, Teachman BA, *et al.* Attempted Training of Alcohol Approach and Drinking Identity Associations in US Undergraduate Drinkers: Null Results from Two Studies. *PLoS ONE* 2015;**10**:e0134642. doi:10.1371/journal.pone.0134642

53 Becker D, Jostmann NB, Holland RW. Does approach bias modification really work in the eating domain? A commentary on Kakoschke et al. (2017). *Addict Behav* 2018;**77**:293–4. doi:10.1016/j.addbeh.2017.02.025

54 Brunoni AR, Moffa AH, Sampaio-Junior B, *et al.* Trial of Electrical Direct-Current Therapy versus Escitalopram for Depression. http://dx.doi.org/10.1056/NEJMoa1612999. 2017. doi:10.1056/NEJMoa1612999

55 Bose A, Shivakumar V, Agarwal SM, *et al.* Efficacy of fronto-temporal transcranial direct current stimulation for refractory auditory verbal hallucinations in schizophrenia: A randomized, double-blind, sham-controlled study. *Schizophrenia Research* 2018;**195**:475–80. doi:10.1016/j.schres.2017.08.047

56 Lupi M, Martinotti G, Santacroce R, *et al.* Transcranial Direct Current Stimulation in Substance Use Disorders: A Systematic Review of Scientific Literature. *J ECT* 2017;**33**:203–9. doi:10.1097/YCT.0000000000000401

For peer review only

57 Dalton B, Campbell IC, Schmidt U. Neuromodulation and neurofeedback treatments in eating disorders and obesity. *Curr Opin Psychiatry* 2017;**30**:458–73. doi:10.1097/YCO.0000000000000361

58 Kekic M, McClelland J, Campbell I, *et al.* The effects of prefrontal cortex transcranial direct current stimulation (tDCS) on food craving and temporal discounting in women with frequent food cravings. *Appetite* 2014;**78**:55–62. doi:10.1016/j.appet.2014.03.010

59 Burgess EE, Sylvester MD, Morse KE, *et al.* Effects of transcranial direct current stimulation (tDCS) on binge eating disorder. *Int J Eat Disord* 2016;**49**:930–6. doi:10.1002/eat.22554

60 Fregni F, Orsati F, Pedrosa W, *et al.* Transcranial direct current stimulation of the prefrontal cortex modulates the desire for specific foods. *Appetite* 2008;**51**:34–41. doi:10.1016/j.appet.2007.09.016

61 Goldman RL, Borckardt JJ, Frohman HA, *et al.* Prefrontal cortex transcranial direct current stimulation (tDCS) temporarily reduces food cravings and increases the self-reported ability to resist food in adults with frequent food craving. *Appetite* 2011;**56**:741–6. doi:10.1016/j.appet.2011.02.013

62 Val-Laillet D, Aarts E, Weber B, *et al.* Neuroimaging and neuromodulation approaches to study eating behavior and prevent and treat eating disorders and obesity. *Neuroimage Clin* 2015;**8**:1–31. doi:10.1016/j.nicl.2015.03.016

63 Heeren A, Baeken C, Vanderhasselt M-A, *et al.* Impact of Anodal and Cathodal Transcranial Direct Current Stimulation over the Left Dorsolateral Prefrontal Cortex during Attention Bias Modification: An Eye-Tracking Study. *PLoS ONE* 2015;**10**:e0124182. doi:10.1371/journal.pone.0124182

64 Fritsch B, Reis J, Martinowich K, *et al.* Direct current stimulation promotes BDNF-dependent synaptic plasticity: potential implications for motor learning. *Neuron* 2010;**66**:198–204. doi:10.1016/j.neuron.2010.03.035

65 Sedgmond Jemma, Lawrence Natalia S., Verbruggen Frederick, *et al.* Prefrontal brain stimulation during food-related inhibition training: effects on food craving, food consumption and inhibitory control. *Royal Society Open Science*;**6**:181186. doi:10.1098/rsos.181186

66 Antal A, Terney D, Poreisz C, *et al.* Towards unravelling task-related modulations of neuroplastic changes induced in the human motor cortex. *Eur J Neurosci* 2007;**26**:2687–91. doi:10.1111/j.1460-9568.2007.05896.x

67 Wiers RW, Gladwin TE, Hofmann W, *et al.* Cognitive Bias Modification and Cognitive Control Training in Addiction and Related Psychopathology: Mechanisms, Clinical Perspectives, and Ways Forward. *Clinical Psychological Science* 2013;**1**:192–212. doi:10.1177/2167702612466547

68 den Uyl TE, Gladwin TE, Rinck M, *et al.* A clinical trial with combined transcranial direct current stimulation and alcohol approach bias retraining. *Addict Biol* 2017;**22**:1632–40. doi:10.1111/adb.12463

69 Ray MK, Sylvester MD, Osborn L, *et al.* The critical role of cognitive-based trait differences in transcranial direct current stimulation (tDCS) suppression of food craving and eating in frank obesity. *Appetite* 2017;**116**:568–74. doi:10.1016/j.appet.2017.05.046

For peer review only

For peer review only - http://bmjopen.bmj.com/site/about/guidelines.xhtml

70 Gluck ME, Alonso-Alonso M, Piaggi P, *et al.* Noninvasive neuromodulation targeted to the lateral prefrontal cortex induces changes in energy intake and weight loss in obesity. *Obesity (Silver Spring)* 2015;**23**:2149–56. doi:10.1002/oby.21313

71 Jauch-Chara K, Kistenmacher A, Herzog N, *et al.* Repetitive electric brain stimulation reduces food intake in humans. *Am J Clin Nutr* 2014;**100**:1003–9. doi:10.3945/ajcn.113.075481

72 Eldridge SM, Chan CL, Campbell MJ, *et al.* CONSORT 2010 statement: extension to randomised pilot and feasibility trials. *BMJ* 2016;**355**:i5239. doi:10.1136/bmj.i5239

73 Chan A-W, Tetzlaff JM, Altman DG, *et al.* SPIRIT 2013 Statement: defining standard protocol items for clinical trials. *Rev Panam Salud Publica* 2015;**38**:506–14.

74 Physical status: the use and interpretation of anthropometry. Report of a WHO Expert Committee. *World Health Organ Tech Rep Ser* 1995;**854**:1–452.

75 Association AP. *Diagnostic and Statistical Manual of Mental Disorders (DSM-5®)*. American Psychiatric Pub 2013.

76 Brunoni AR, Nitsche MA, Bolognini N, *et al.* Clinical research with transcranial direct current stimulation (tDCS): challenges and future directions. *Brain Stimul* 2012;**5**:175–95. doi:10.1016/j.brs.2011.03.002

77 Moher D, Schulz KF, Altman DG. The CONSORT statement: revised recommendations for improving the quality of reports of parallel-group randomised trials. *Lancet* 2001;**357**:1191–4.

78 Schulz KF, Altman DG, Moher D, *et al.* CONSORT 2010 statement: updated guidelines for reporting parallel group randomized trials. *Ann Intern Med* 2010;**152**:726–32. doi:10.7326/0003-4819-152-11-201006010-00232

79 Lancaster GA, Dodd S, Williamson PR. Design and analysis of pilot studies: recommendations for good practice. *J Eval Clin Pract* 2004;**10**:307–12. doi:10.1111/j.2002.384.doc.x

80 Julious SA. Sample size of 12 per group rule of thumb for a pilot study. *Pharmaceutical Statistics* 2005;**4**:287–91. doi:10.1002/pst.185

81 Schmidt U, Oldershaw A, Jichi F, *et al.* Out-patient psychological therapies for adults with anorexia nervosa: randomised controlled trial. *Br J Psychiatry* 2012;**201**:392–9. doi:10.1192/bjp.bp.112.112078

82 Schmidt U, Renwick B, Lose A, *et al.* The MOSAIC study - comparison of the Maudsley Model of Treatment for Adults with Anorexia Nervosa (MANTRA) with Specialist Supportive Clinical Management (SSCM) in outpatients with anorexia nervosa or eating disorder not otherwise specified, anorexia nervosa type: study protocol for a randomized controlled trial. *Trials* 2013;**14**:160. doi:10.1186/1745-6215-14-160

83 Fairburn CG, Beglin S, editors. *Cognitive Behavior Therapy and Eating Disorders.* New York: Guildford Press 2008.

84 Deurenberg P, Weststrate JA, Seidell JC. Body mass index as a measure of body fatness: age- and sex-specific prediction formulas. *British Journal of Nutrition* 1991;**65**:105–14. doi:10.1079/BJN19910073

For peer review only - http://bmjopen.bmj.com/site/about/guidelines.xhtml

85 Rinck M, Becker ES. Approach and avoidance in fear of spiders. *J Behav Ther Exp Psychiatry* 2007;**38**:105–20. doi:10.1016/j.jbtep.2006.10.001

86 Blechert J, Meule A, Busch NA, *et al.* Food-pics: an image database for experimental research on eating and appetite. *Front Psychol* 2014;**5**:617. doi:10.3389/fpsyg.2014.00617

87 De Houwer J, Crombez G, Baeyens F, *et al.* On the generality of the affective Simon effect. *Cognition & Emotion* 2001;**15**:189–206. doi:10.1080/02699930125883

88 Steinglass J, Foerde K, Kostro K, *et al.* Restrictive food intake as a choice--a paradigm for study. *Int J Eat Disord* 2015;**48**:59–66. doi:10.1002/eat.22345

89 Foerde K, Gianini L, Wang Y, *et al.* Assessment of test-retest reliability of a food choice task among healthy individuals. *Appetite* 2018;**123**:352–6. doi:10.1016/j.appet.2018.01.010

90 Hare TA, Camerer CF, Rangel A. Self-control in decision-making involves modulation of the vmPFC valuation system. *Science* 2009;**324**:646–8. doi:10.1126/science.1168450

91 Cepeda-Benito A, Gleaves DH, Williams TL, *et al.* The development and validation of the state and trait food-cravings questionnaires. *Behavior Therapy* 2000;**31**:151–73. doi:10.1016/S0005-7894(00)80009-X

92 Meule A, Lutz A, Vögele C, *et al.* Food cravings discriminate differentially between successful and unsuccessful dieters and non-dieters. Validation of the Food Cravings Questionnaires in German. *Appetite* 2012;**58**:88–97. doi:10.1016/j.appet.2011.09.010

93 Kekic M, McClelland J, Bartholdy S, *et al.* Single-Session Transcranial Direct Current Stimulation Temporarily Improves Symptoms, Mood, and Self-Regulatory Control in Bulimia Nervosa: A Randomised Controlled Trial. *PLoS ONE* 2017;**12**:e0167606. doi:10.1371/journal.pone.0167606

94 Meule A, Hermann T, Kübler A. A short version of the Food Cravings Questionnaire-Trait: the FCQ-T-reduced. *Front Psychol* 2014;**5**:190. doi:10.3389/fpsyg.2014.00190

95 Lowe MR, Butryn ML, Didie ER, *et al.* The Power of Food Scale. A new measure of the psychological influence of the food environment. *Appetite* 2009;**53**:114–8. doi:10.1016/j.appet.2009.05.016

96 Gearhardt AN, Corbin WR, Brownell KD. Development of the Yale Food Addiction Scale Version 2.0. *Psychol Addict Behav* 2016;**30**:113–21. doi:10.1037/adb0000136

97 Gearhardt AN, White MA, Masheb RM, *et al.* An examination of food addiction in a racially diverse sample of obese patients with binge eating disorder in primary care settings. *Compr Psychiatry* 2013;**54**:500–5. doi:10.1016/j.comppsych.2012.12.009

98 Steward T, Mestre-Bach G, Vintró-Alcaraz C, *et al.* Food addiction and impaired executive functions in women with obesity. *European Eating Disorders Review* 2018;**26**:574–84. doi:10.1002/erv.2636

99 Meule A, Heckel D, Kübler A. Factor Structure and Item Analysis of the Yale Food Addiction Scale in Obese Candidates for Bariatric Surgery. *European Eating Disorders Review* 2012;**20**:419–22. doi:10.1002/erv.2189

For peer review only - http://bmjopen.bmj.com/site/about/guidelines.xhtml

100 Clark SM, Saules KK. Validation of the Yale Food Addiction Scale among a weight-loss surgery population. *Eating Behaviors* 2013;**14**:216–9. doi:10.1016/j.eatbeh.2013.01.002

101 Carter JC, Van Wijk M, Rowsell M. Symptoms of 'food addiction' in binge eating disorder using the Yale Food Addiction Scale version 2.0. *Appetite* 2019;**133**:362–9. doi:10.1016/j.appet.2018.11.032

102 Robinson E, Haynes A, Hardman CA, *et al.* The bogus taste test: Validity as a measure of laboratory food intake. *Appetite* 2017;**116**:223–31. doi:10.1016/j.appet.2017.05.002

103 Odum AL, Baumann AAL, Rimington DD. Discounting of delayed hypothetical money and food: effects of amount. *Behav Processes* 2006;**73**:278–84. doi:10.1016/j.beproc.2006.06.008

104 Teslovich T, Freidl EK, Kostro K, *et al.* Probing behavioral responses to food: development of a food-specific go/no-go task. *Psychiatry Res* 2014;**219**:166–70. doi:10.1016/j.psychres.2014.04.053

105 Verbruggen F, Logan GD. Response inhibition in the stop-signal paradigm. *Trends Cogn Sci (Regul Ed)* 2008;**12**:418–24. doi:10.1016/j.tics.2008.07.005

106 Manasse SM, Goldstein SP, Wyckoff E, *et al.* Slowing down and taking a second look: Inhibitory deficits associated with binge eating are not food-specific. *Appetite* 2016;**96**:555–9. doi:10.1016/j.appet.2015.10.025

107 Gross JJ, John OP. Individual differences in two emotion regulation processes: implications for affect, relationships, and well-being. *J Pers Soc Psychol* 2003;**85**:348–62.

108 Watson D, Clark LA, Tellegen A. Development and validation of brief measures of positive and negative affect: the PANAS scales. *J Pers Soc Psychol* 1988;**54**:1063–70.

109 Lovibond SH, Lovibond PF. *Manual for the Depression Anxiety Stress Scales (2nd. ed.)*. Sydney Psychology Foundation 1995.

110 Jones EB, Sharpe L. Cognitive bias modification: A review of meta-analyses. *J Affect Disord* 2017;**223**:175–83. doi:10.1016/j.jad.2017.07.034

111 Song S, Zilverstand A, Gui W, *et al.* Effects of single-session versus multi-session non-invasive brain stimulation on craving and consumption in individuals with drug addiction, eating disorders or obesity: A meta-analysis. *Brain Stimul* Published Online First: 27 December 2018. doi:10.1016/j.brs.2018.12.975

112 Neumann R, Strack F. Approach and avoidance: the influence of proprioceptive and exteroceptive cues on encoding of affective information. *J Pers Soc Psychol* 2000;**79**:39–48.

113 Vrijsen JN, Fischer VS, Müller BW, *et al.* Cognitive bias modification as an add-on treatment in clinical depression: Results from a placebo-controlled, single-blinded randomized control trial. *J Affect Disord* 2018;**238**:342–50. doi:10.1016/j.jad.2018.06.025

114 Becker ES, Ferentzi H, Ferrari G, *et al.* Always Approach the Bright Side of Life: A General Positivity Training Reduces Stress Reactions in Vulnerable Individuals. *Cognit Ther Res* 2016;**40**:57–71. doi:10.1007/s10608-015-9716-2

For peer review only - http://bmjopen.bmj.com/site/about/guidelines.xhtml

115 Brockmeyer T, Schmidt U, Friederich H-C. The ABBA study - approach bias modification in bulimia nervosa and binge eating disorder: study protocol for a randomised controlled trial. *Trials* 2016;**17**:466. doi:10.1186/s13063-016-1596-6

116 Poreisz C, Boros K, Antal A, *et al.* Safety aspects of transcranial direct current stimulation concerning healthy subjects and patients. *Brain Research Bulletin* 2007;**72**:208–14. doi:10.1016/j.brainresbull.2007.01.004

117 Hutson PH, Balodis IM, Potenza MN. Binge-eating disorder: Clinical and therapeutic advances. *Pharmacol Ther* 2018;**182**:15–27. doi:10.1016/j.pharmthera.2017.08.002

118 Vroling MS, Wiersma FE, Lammers MW, *et al.* Predicting Dropout from Intensive Outpatient Cognitive Behavioural Therapy for Binge Eating Disorder Using Pre-treatment Characteristics: A Naturalistic Study. *Eur Eat Disord Rev* 2016;**24**:494–502. doi:10.1002/erv.2474

119 Marinilli Pinto A, Guarda AS, Heinberg LJ, *et al.* Development of the eating disorder recovery self-efficacy questionnaire. *Int J Eat Disord* 2006;**39**:376–84. doi:10.1002/eat.20256

120 Patton JH, Stanford MS, Barratt ES. Factor structure of the Barratt impulsiveness scale. *J Clin Psychol* 1995;**51**:768–74.

121 Bohn K, Fairburn CG. *The Clinical Impairment Assessment Questionnaire (CIA 3.0). In Cognitive Behavior Therapy and Eating Disorders.* New York: Guildford Press 2008.

122 Hoerger M, Quirk SW, Weed NC. Development and validation of the Delaying Gratification Inventory. *Psychol Assess* 2011;**23**:725–38. doi:10.1037/a0023286

For peer review only

For peer review only - http://bmjopen.bmj.com/site/about/guidelines.xhtml

Figure 1 Study procedure. The 3 assessment time points are baseline, post-assessment and follow-up. IG1 is intervention group 1; IG2 is intervention group 2, CG is the wait list control group.

Table 1 Study schedule of measurement and testing time points

	**Screening of potential participants**	**Baseline assessment (all participants)**	**Training: 6 sessions of ABM + real tDCS**	**Training: 6 sessions of ABM + sham tDCS**	**Post-assessment (all participants**	**Follow-up (all participants)**
**Approximate time since baseline**	-------	-------	**0-3 weeks**	**0-3 weeks**	**3 weeks**	**7 weeks**
Informed consent		X				
EDDS, SCID-I	X					
TDCS safety screening	X					
Demographic information		X				
EDE-Q[83]		X			X	X
Inhibitory control tasks; Go/No-Go task[104], SST[106]		X			X	X
Delay Discounting Task with Money and Food[103]		X			X	X
Food related tasks; Food Choice Task[88], FCT, Bogus Taste Test[102]		X			X	
Approach bias assessment tasks; F-AAT[85], SRC[87]		X			X	X
Questionnaires & scales (incl. at home); EDRSQ[119], FCQ-T-r[94], PFS[95], YFAS 2.0[96], ERQ[107], PANAS[108], BIS-11[120], CIA[121], DGI[122], DASS-21[109]		X			X	X
Pre-[tDCS + ABM] measures: -Multiple VAS, blood pressure, pulse			X	X		
Real or sham tDCS to dLPFC			X	X		
Approach Bias Modification training			X	X		
Post-[tDCS + ABM] measures: -Multiple VAS, blood pressure, pulse			X	X		
Tolerance, discomfort and side effects			X	X		
Acceptability questionnaire						X
Blinding assessment questionnaire						X

EDDS; Eating Disorder Diagnostic Screen, SCID-I; Structured Clinical Interview for Diagnostic and Statistical Manual (DSM) Axis I Disorders, EDE-Q; Eating Disorder Examination, SST; Stop Signal Task, FCT; Food Challenge Task, F-AAT; Food Approach-Avoidance Task, SRC; Stimulus Response Compatibility Task, EDRSQ; Eating Disorder Recovery Self Efficacy Questionnaire, FCQ-T-r; Food Cravings Questionnaire Trait Version – reduced, PFS; The 21-item Power of Food Scale, YFAS 2.0; The Yale Food Addiction Scale Version 2.0, ERQ; Emotion Regulation Questionnaire, PANAS; Positive and Negative Affect Schedule, BIS-11; Barrett Impulsiveness Scale, CIA; Clinical Impairment Assessment, DGI; Delayed Gratification Inventory, DASS-21; Depression, Anxiety and Stress Scale.

For peer review only

For peer review only - http://bmjopen.bmj.com/site/about/guidelines.xhtml

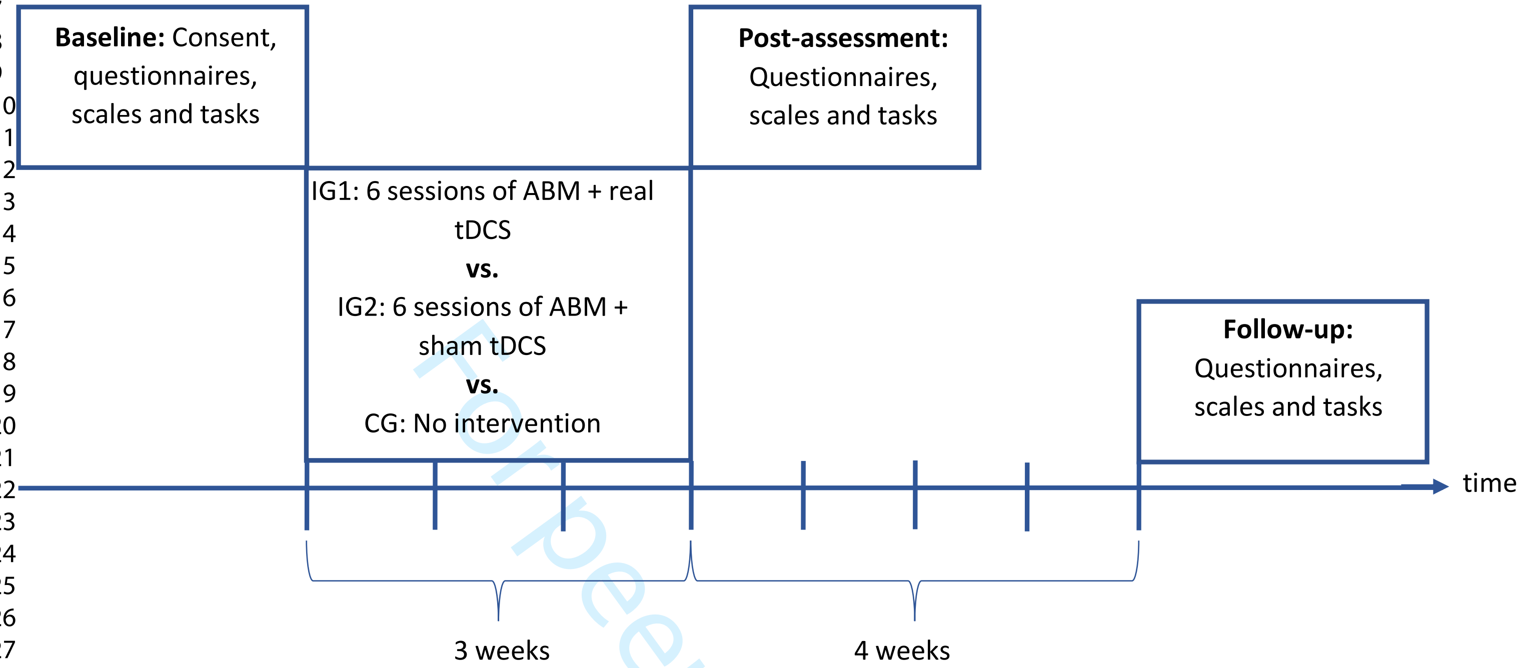
Baseline: Consent, questionnaires, scales and tasks
Post-assessment: Questionnaires, scales and tasks
IG1: 6 sessions of ABM + real tDCS
vs.
IG2: 6 sessions of ABM + sham tDCS
vs.
CG: No intervention
Follow-up: Questionnaires, scales and tasks
time
3 weeks
4 weeks

Supplementary File

Appendix A: ICARUS study task, questionnaire, measure and scale information

(1) *Approach bias assessment tasks*

(i) Food Approach-Avoidance Task (F-AAT) (Rinck & Becker, 2007): The F-AAT is a computerised task that measures approach and avoidance behaviour by means of joystick movements in response to food and neutral stimuli presented on a computer screen. This task will be used to assess approach bias towards visual cues of high-calorie food (expected target cognitive mechanism of intervention). Images of palatable edible foods (i.e. chocolate, pizza) and non-edible objects (i.e. sponges, stapler) are used as in previously (Brockmeyer et al., 2019). The assessment version of the AAT is identical to the treatment version except that the required response is unrelated to the picture content (i.e. food and neutral stimuli are presented equally often in round and rectangular, i.e. push and pull, format). Format movement assignments are counterbalanced among participants (i.e., half push round pictures and half push rectangular pictures). When the joystick is pulled, the picture grows bigger, and diminishes in size when the joystick is pushed. Zooming-in and zooming-out via joystick movements enacts motions of approaching and avoiding respectively and thus combines the proprioceptive (arm movement) and exteroceptive (zooming feature) cues of approach and avoidance behaviour (Neumann & Strack, 2000; Brockmeyer et al., 2019). The assessment version of the AAT consists of 80 trials (40 food item pictures and 40 non-food item pictures). To evaluate approach bias towards food, a compatibility score is calculated by subtracting the median reaction times (RTs) of compatible trials (i.e., RT pull food + RT push nonfood) from median RTs of incompatible trials (i.e., RT push food + RT pull nonfood) (Brockmeyer et al., 2019; Becker et al., 2016; Vrijsen et al., 2018). A positive value indicates a food-specific approach bias (i.e. the participant is faster at pulling than pushing food pictures, relative to the approach bias towards non-food), whereas a negative value indicates an avoidance bias. This task will be performed using Inquisit 5 (Millisecond Software).

(ii) The Stimulus Response Compatibility Task (SRC) (De Houwer, Crombez, Baeyens, & Hermans, 2001): In this task, participants respond to images on a computer screen by pressing keys on the keyboard. Pictures are presented in the centre of the screen with a manikin (12 mm high) positioned 33 mm above or below the picture. Participants are required to categorise the presented pictures by making an approach response (pressing the *up* or *down* key to move the manikin toward the picture) or an avoidance response (pressing the *up* or *down* key to move the manikin away from the picture). After making a correct response, an animation is shown of the manikin walking toward the picture (approach) or away from the picture (avoidance) for 1,000 ms. After making an incorrect response, a red cross appears on the screen for 500 ms, after which the next trial starts. Fourteen food and fourteen non-food pictures will be used from the food-pics database (Blechert, Meule, Busch & Ohla, 2014). The experiment comprises 8 practice trials and 56 experimental trials. Bias scores will be calculated by subtracting the mean of approach food/avoid non-food trials from the mean of avoid food/approach non-food trials. A positive score indicates a food-related approach bias, with higher scores indicative of stronger biases. This task will be performed using Inquisit 5 (Millisecond Software).

For peer review only - http://bmjopen.bmj.com/site/about/guidelines.xhtml

(2) Food Choice Task

The Food Choice Task (Hare, Camerer & Rangel., 2009) adapted for eating disorders (Steinglass et al., 2015) is a computer-based paradigm that measures responses to images of foods to assess food attitudes and characteristics of eating behaviour. Food stimuli is used to investigate how individuals make decisions about what to eat, and measures decision-making around food by directly probing personal preferences. There are no learning requirements and individualised assessments of food along two dimensions (healthiness and tastiness) allow the tasks to be used in diverse populations with differing valuations of food. The task consists of three phases. In each phase participants are presented with 43 images of food items. The food items represent an array of dietary options (Steinglass et al., 2015). Twenty-five food items are low fat (<30% calories from fat) and 18 are high fat (>30% calories from fat), as determined by Foerde et al. (2018). In the Health phase, participants rate the healthiness of each food item on a 5-point scale (*1 = "Unhealthy", 5 = "Healthy"*). In the Taste phase, participants rate the tastiness of each food item in a similar fashion, (*1 = "Bad", 5 = "Good"*). In the Choice phase, in each trial participants choose between the presented food item and a "Neutral" reference food item (rated as 3 in both Health and Taste phases). If no item is rated 3 on both scales, an item rated 3 on Health and greater than 3 on the Taste scale is selected as a reference food. Good test-retest reliability of the FCT suggests that it is suitable for measuring food-based decision-making in studies with multiple assessment points (Foerde et al., 2018).

(3) *State food craving - Food craving after cue exposure task*

The Food Challenge Task (FCT) (Kekic et al., 2017; Meule et al., 2018) will be used to examine cue-induced food craving. In this task, participants rate their state food craving using the Food Cravings Questionnaire State Version (Cepeda-Benito et al., 2000; Meule et al., 2012) before and after being presented with a video on a computer screen of foods shown to be highly appetitising and to elevate hunger levels (Kekic et al., 2017). The questionnaire consists of 15 items that measure the strength of food cravings (i.e. *I would feel more alert if I could satisfy my craving*). Participants are asked to indicate how much they agree with each statement 'at this very moment' using a five-point scale (from 1 'strongly disagree' to 5 'strongly agree'). There are five craving subscales; intense desire to eat, anticipation of relief from negative states, physiological craving, preoccupation with food or lack of control over eating and anticipation of positive reinforcement. Scores can be calculated for specific subscales or a total score can be calculated (ranging from 15 to 75). This questionnaires will be completed using Inquisit 5 (Millisecond Software), and the video shown using QuickTime player.

(4) *Trait food craving*

The Yale Food Addiction Scale Version 2.0 (YFAS 2.0; Gearhardt, Corbin, & Brownell, 2016) is the most commonly used instrument to assess food-related addictive behaviours (Steward et al., 2018). This self-report questionnaire consists of 35 items scored on an 8-point Likert scale (from 0 = never to 7 = every day) and is adapted to assess addictive eating behaviours based on DSM-5 substance-related and addictive disorders criteria (APA, 2013). It refers specifically to consumption of foods high in fat, sugar, salt or refined carbohydrates. It

For peer review only - http://bmjopen.bmj.com/site/about/guidelines.xhtml

includes items that assess specific criteria, such as diminished control over consumption, a persistent desire or repeated unsuccessful attempts to quit, withdrawal, and clinically significant impairment (i.e. *'I kept eating in the same way even though my eating caused emotional problems')*. The YFAS includes two scoring options: 1) a "symptom count" ranging from 0 to 7 that reflects the number of addiction-like criteria endorsed and 2) a categorical scoring option that classifies respondents as having either no, mild, moderate or severe 'food addiction'. The YFAS has received psychometric support in binge eating populations (Gearhardt, White et al., 2013; Carter, Van Wijk & Rowsell, 2019), and obese bariatric surgery patients (Clark & Saules, 2013; Meule, Heckel, & Kübler, 2012). The YFAS 2.0 was developed to maintain consistency with the current diagnostic understanding of addiction and to improve the psychometric properties of the original YFAS. Exceeding the food addiction threshold was more strongly associated with obesity for the YFAS 2.0 than the original YFAS. The YFAS 2.0 has demonstrated good internal consistency (Carter, Van Wijk & Rowsell, 2019), as well as convergent, discriminant and incremental validity (Gearhardt, Corbin & Brownell, 2016).

(5) *Food intake in a Bogus Taste Test*

Actual food consumption will be measured by means of a Bogus Taste Test (Robinson et al., 2017); a food consumption test presented under the guise of a taste test. Participants will be instructed to rate 3 bowls of highly palatable high-calorie food items (chocolate, sweets, crisps) in terms of their visual attractiveness, smell, and taste on a paper form. The researcher will inform the participant that she/he will leave the room for 10 minutes and during this time they can complete their ratings and are free to eat as much of the offered items as they like. A small bin with lid will be provided and participants will be instructed to consume as much food as they need or want, and to discard the remainder of the food items in the bin before 10 minutes are over. After the participant has left, the discarded food items will be recovered from the bin, sorted, and placed in their original bowls. Consumption will be determined by weighing the bowls both before and after the "taste test" and the difference in weight from pre-to post-assessment will be converted into calories and used as a measure of food intake.

(6) *Delay discounting*

Delay Discounting Task with Money and Food (Odum, Baumann, & Rimington, 2006): Participants indicate their preferences in a series of choices for two hypothetical outcome types: immediate versus delayed food and immediate versus delayed money. Participants make choices involving either relatively small maximum amounts of food (10 dollars worth) and money (10 dollars) or for relatively large maximum amounts of food (100 dollars worth) and money (100 dollars). Performance on this task can be used to study self-regulation, delayed gratification and valuation of reward. This task will be performed using Inquisit 5 (Millisecond Software).

(7) *Inhibitory control*

(i) Go/No-Go task: The cued go/no go task is a useful measure of impulse control in clinical populations. This task is a classic test of executive function, requiring effortful response inhibition. The food specific version of the cued go no-go task (Teslovich et al., 2014) measures impulsivity and response inhibition with respect to appetising food and nonfood items (i.e. toys), via assessing the ability to inhibit instigated, prepotent responses. The task

For peer review only - http://bmjopen.bmj.com/site/about/guidelines.xhtml

manipulates response prepotency by presenting a preliminary go or no-go cue before the actual go or no-go target is displayed. The cues provide information concerning the probability that a go or no-go target will be presented. The cue-target relationship is manipulated so that the cues have a high probability of correctly signaling a go or no-go target (valid cues), and a low probability of incorrectly signaling a target (invalid cues). Valid cues tend to facilitate response inhibition and speed response execution, whereas invalid cue cues tend to impair response inhibition and slow response execution. The set of stimuli consists of 30 colour images of common high- (8) and low-calorie (7) foods and common toys (15). The outcome variables include: (1) overall reaction time (RT) in milliseconds during correct "go" trials, (2) rate of omission errors (missed "go" trials), and (3) false alarm rate (rate at which participants erroneously press to a no-go stimulus). This task will be performed using Inquisit 5 (Millisecond Software).

(i) Stop Signal Task (SST): This is a task measuring inhibitory control. Participants are required to engage in a computer task but withhold their response in the presence of a stop signal. This SST includes food-specific and neutral non-food stimuli in the same task (adaptation of Manasse et al., 2016). This allows for isolation of any unique food specific inhibitory control deficits from general difficulties inhibiting responses. The outcome measure is the stop signal reaction time (SSRT). The SSRT is calculated for each set of stimuli (i.e., SSRT stimulus type) for each subject by subtracting the average stop signal delay from the average reaction time on "go" trials (Verbruggen & Logan, 2008). The recording accuracy of reaction time and stop signal delay measurement is in milliseconds. A smaller SSRT is indicative of greater inhibitory control and a larger SSRT reflects weaker/impaired inhibitory control. This task will be performed using Inquisit 5 (Millisecond Software).

(8) *Mood and emotion regulation*
The Emotion Regulation Questionnaire (ERQ) (Gross & John, 2003): A 10-item scale designed to measure respondents' tendency to regulate their emotions in two ways: (1) cognitive reappraisal and (2) expressive suppression. Respondents answer each item on a 7-point Likert-type scale ranging from 1 (strongly disagree) to 7 (strongly agree). This will be administered at pre-assessment, post-assessment and follow-up.

Positive and Negative Affect Schedule (PANAS) (Watson, Clarke & Tellegen, 1988): The PANAS consists of two 10-item self-report scales which measure positive and negative affect. On a Likert scale ranging from 0 (very slightly or not at all) to 4 (extremely), participants rate the extent to which they have experienced each of the 20 descriptors within a particular time frame ("right now" in the current study). Two scores are generated: positive (PANAS-positive) and negative (PANAS- negative) affect. This will be administered at pre-assessment, post-assessment and follow-up.

Depression, Anxiety and Stress Scale (21-item version; DASS-21) (Lovibond & Lovibond, 1995): This is a 21 item self-report questionnaire which aims to evaluate mood, anxiety and stress levels over the previous week. The DASS-21 will be administered at pre-assessment, post-assessment and follow-up.

(9) *Within session measures (immediately after each [ABM+real/sham tDCS] treatment session)*

For peer review only - http://bmjopen.bmj.com/site/about/guidelines.xhtml

(i) Paper-based Visual Analogue Scales (VAS) assessing current hunger, feeling of fullness, urge to eat, urge to binge eat, feeling low, level of tension, level of stress, level of anxiety. These scales consist of a 10cm line. Participants are requested to indicate on this line a degree or level of a specific emotion or behavioural urge. There are indications of what range (e.g. from 'not at all' to 'extremely').

(ii) Tolerance, discomfort and side effects: An evaluation of discomfort with the training session (tDCS and ABM aspects separately) will be completed with another paper-based 10cm VAS (rated from none to extreme discomfort). Participants will be asked to report any side effects in an open ended question.

Appendix B: Consent form for all participants

CONSENT FORM

IRAS Project ID: 244170
Research Ethics Committee reference number: 18/NW/0648

King's College London

NHS South London and Maudsley NHS Foundation Trust

Please complete this form after you have read the information sheet and listened to an explanation about the research.

Title of Study: ICARUS - An Investigation of Approach Bias Modification Training (ABM) and Transcranial Direct Current Stimulation (tDCS) in Binge Eating Disorder
Name of Researcher: Gemma Gordon

Thank you for considering taking part in this research. The person organising the research must explain the project to you before you agree to take part. If you have any questions arising from the information sheet or explanation already given to you, please ask the researcher before you decide whether to join in. You will be given a copy of this consent form to keep and refer to at any time.

Please initial box

1. I confirm that I have read the information sheet dated 18.09.2018 (version 3) for the above study. I have had the opportunity to consider the information, ask questions and have had these answered satisfactorily. ☐

2. I understand that my participation is voluntary and that I am free to withdraw at any time without giving a reason, without my medical care or legal rights being affected. ☐

3. I consent to the processing of my personal information for the purposes explained to me. I understand that such information will be handled in accordance with the terms of the General Data Protection Regulation (GDPR) 2018. I understand that confidentiality and anonymity will be maintained and it will not be possible to identify me in any publications. ☐

For peer review only - http://bmjopen.bmj.com/site/about/guidelines.xhtml

4. I know that if I would like to, I can contact the research team and request a written summary of the study findings. ☐

5. I understand that I must not take part if I have any of the conditions listed in the exclusion criteria. ☐

6. I understand that during study participation, I must inform the researcher of any changes in my medication or of any new medical diagnoses made. ☐

7. I agree to my General Practitioner (GP) being informed of my participation in this study. ☐

8. **I consent/do not consent** to be contacted in the future by King's College London researchers who would like to invite me to participate in follow up studies to this project, or in future studies of a similar nature. ☐

9. I agree to take part in the above study. ☐

Participant's Statement:

I ____

agree that the research project named above has been explained to me to my satisfaction and I agree to take part in the study. I have read both the notes written above and the Information Sheet about the project, and understand what the research study involves.

Signed **Date**

Investigator's Statement:

I ____

Confirm that I have carefully explained the nature, demands and any foreseeable risks (where applicable) of the proposed research to the participant.

Signed **Date**

For administration purposes, please indicate your preference below

Q: In what format would you like to complete the at-home assessment questionnaires?

For peer review only

Tick '√' in one box to indicate your choice

1. I would like to complete the assessment questionnaires online ☐

2. I would like to receive a hardcopy paper version of the questionnaires in a stamped addressed envelope, to complete and post back to the researcher within 36 hours of the in-person assessment visit. ☐

Enquiries:

Gemma Gordon (gemma.gordon@kcl.ac.uk)
Department of Psychological Medicine
Section of Eating Disorders
KCL Institute of Psychology, Psychiatry and Neuroscience,
16 De Crespigny Park
London, SE5 8AF
Phone: 0207 848 5608

Appendix C: Information sheet for all participants

PARTICIPANT INFORMATION SHEET

ICARUS: An Investigation of Approach Bias Modification Training (ABM) and Transcranial Direct Current Stimulation (tDCS) in Binge Eating Disorder

IRAS Project ID: 244170
Research Ethics Committee reference number: 18/NW/0648

We would like to invite you to participate in this postdoctoral research project which is being conducted by a PhD student for research and educational purposes. You should only participate if you want to; choosing not to take part will not disadvantage you in any way. Before you decide whether you want to take part, it is important for you to understand why the research is being done and what your participation will involve. Please take time to read the following information carefully and discuss it with others if you wish. Part 1 tells you the purpose of this study and what will happen to you if you take part. Part 2 gives you more detailed information about the conduct of the study. Please ask us if there is anything that is not clear or if you would like more information.

PART ONE

What is the purpose of the study?

Psychological therapy as a main treatment for Binge Eating Disorder (BED) may not be effective for many people and may not be readily accessible in some areas. Medical treatments for BED can have side effects and often do not remain effective in the long-term. Therefore, there is an ongoing need for the development of new treatments.

For peer review only - http://bmjopen.bmj.com/site/about/guidelines.xhtml

Computerised approach bias modification training (ABM) is a specific form of cognitive bias modification (CBM) that has been used to successfully treat mental disorders such as anxiety, depression, and addictive disorders. This technique involves several sessions of computerised training, a procedure which has shown to be effective in reducing the severity of some eating disorder symptoms in people with BED and Bulimia Nervosa (BN). ABM works as such; automatic approach and avoidance tendencies towards food-related cues are modified by repeated training of arm movements in front of a computer screen. ABM has shown to reduce approach tendencies and attention towards food cues in a subclinical sample of eating disorders involving binge eating, but its efficacy on these features in people with full-syndrome eating disorders remains unclear. Further research is needed to examine if ABM is effective in reducing the frequency of binge eating episodes in people with BED.

Transcranial direct current stimulation (tDCS) is a non-invasive technique that is capable of stimulating specific brain areas. Research shows that the frontal areas of the brain play a role in the development and maintenance of eating disorders, including BED. Stimulating these brain areas to alter their functioning is therefore believed to have the potential to reduce eating disorder symptoms. This involves the delivery of a low electrical current via small electrodes placed on the scalp. This procedure is widely used in research and is being applied in clinical settings. Recent research using tDCS on people with BED has suggested that it may be helpful in reducing immediate food intake and cravings, and may decrease the frequency of a desire to binge eat at home after the treatment.

Combining ABM and tDCS

Previous studies suggest that these two techniques potentially help people better regulate their behaviours through similar mechanisms in the brain. Delivering both treatments together at the same time may have a stronger effect on reducing eating disorder symptoms in people with BED than either of the treatments alone. This will be the first time that this specific combination of interventions is conducted on people with an eating disorder.

In the present study, we aim to investigate combined ABM and tDCS as a treatment for BED by comparing the effect of 6 sessions of (ABM + real tDCS) vs. (ABM + placebo tDCS) across a 3-week period in adult men and women with BED. We will also compare these two groups against a control group. Participants will be allocated by chance to either one of two intervention groups, or to the control group. Participants in the intervention groups will receive 6 sessions of ABM delivered simultaneously with either real tDCS or a placebo version of tDCS. Participants assigned to the control group will not receive any intervention. We will measure eating disorder symptoms and other outcomes in all participants at baseline, post-treatment, and at the 4-week follow-up to assess outcomes of each study group. In particular, we are interested in changes in the frequency of binge eating and craving, and thought processes and emotions related to food and eating. We will also ask participants about their experience of this treatment. Participants assigned to the control condition will be offered 6 sessions of (ABM + real tDCS) after they have completed their involvement in the study.

Why have I been invited?

For peer review only - http://bmjopen.bmj.com/site/about/guidelines.xhtml

You are invited to participate if you are a male or female aged between 18 and 70 who has a current diagnosis of binge eating disorder (BED). We will be recruiting 66 participants in total.

Do I have to take part?
You do not have to take part in this experiment; it is your choice. If you decide to take part, you will be asked to sign three identical consent forms. You will be free to withdraw from the study at any time without giving a reason. Whether you decide to take part or not will in no way influence your care or the timing of your treatment.

What will happen to me if I take part and what will I have to do?
If you decide you want to participate you will firstly be asked to engage in a telephone conversation with the researcher (lasting approximately 20 minutes) to confirm that you are eligible to take part. If you are, you will be invited to the Institute of Psychiatry, Psychology and Neuroscience (Kings College London, Denmark Hill Campus) for a baseline assessment session on a day that is convenient for both you and the researcher, in either the morning or the afternoon. On this day, the researcher will discuss the study with you in person, answer your questions, and if you are happy to take part, we will ask you to sign three copies of a consent form: one for you to keep, one for us to keep, and one that will be sent to your general practitioner (GP).

This baseline assessment visit is longer that the treatment visits and is comprised of an in-person visit and online questionnaires to complete at home. During the visit, you will give informed study consent, complete two questionnaires, neuropsychological tasks (brain puzzles), and a food task for which you will be asked to rate different foods. This visit also involves an assessment version of the approach bias modification (ABM) training programme, which is a computer based-task involving pushing and pulling a joystick in response to shapes appearing on the computer screen. Weight and height will be measured at each assessment, and participants may choose not to see the figures recorded. Assessment visits will last 65-75 minutes. Within 36 hours after the in-person visit, you will be emailed a link to a series of questionnaires (assessing mood and eating disorder-related thoughts and habits) which will take 30-45 minutes to complete. There is also the option to complete these assessment questionnaires in hardcopy paper format.

You will then be then randomised and informed of your randomly assigned study condition within a week. If you are assigned to one of the two intervention conditions, you will be asked to attend 6 sessions where you will perform the ABM task while receiving either real or placebo tDCS, in addition to completing the post-treatment and follow-up assessments. If you are assigned to the control group, you will not receive any intervention, and will be asked to attend the post-treatment and follow-up assessments.

All 6 intervention sessions will be identical and last approximately 40–50 minutes. There is no need for any special preparation before the visits. Before and after each intervention session, you will complete some scales related to aspects of your mood and level of hunger. Your blood pressure and pulse will also be measured before and after each intervention session to monitor your wellbeing.
During the (ABM + real/placebo tDCS) session you will sit on a comfortable chair facing a computer screen, with a joystick on a table in front of you. You will wear a plastic

For peer review only - http://bmjopen.bmj.com/site/about/guidelines.xhtml

headband to keep the two tDCS electrodes in place (as shown in the diagram below). The electrodes will be placed in small sponges soaked in a salt water solution, so they might feel a bit wet against your head. The researcher will turn the machine on which will deliver the currents. Depending on your assigned study condition, you will receive real or placebo brain stimulation. The placebo session will be the same as the real session, but the tDCS machine won't deliver any electrical current. Most people can't tell the difference between real and placebo tDCS sessions.

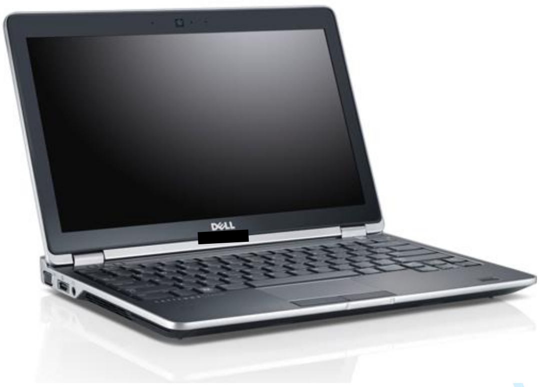

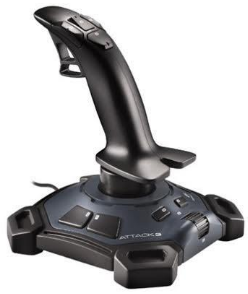

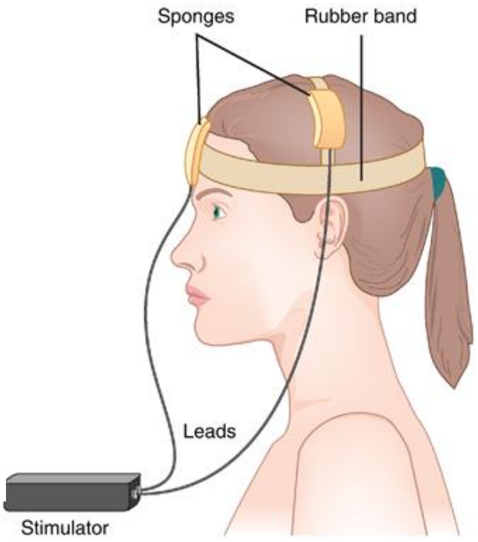

The tDCS will begin a few minutes prior to the start of the computer training programme, to allow participants to become used to the sensation before starting the ABM task on the computer. The training version of the ABM programme is a computer based-task involving pushing and pulling a joystick in response to shapes appearing on the computer screen. The tDCS will also continue for a few minutes after the ABM task has ended. You will then be asked to rate any discomfort experienced during the session due the tDCS.

You will be asked to return to the Institute for 5 more identical intervention sessions within 3 weeks, leaving a gap of at least 24 hours between each session. The post-assessment will be conducted immediately after the 6th intervention session. This in-person visit comprising of the final intervention session plus the post-assessment will therefore last 90-120 minutes, with the online questionnaires to be completed within 36 hours after this visit (45 minutes). The follow-up assessment 4 weeks later will be similar to the baseline assessment. This final visit will therefore take 75 minutes, with the online questionnaires taking 45 minutes to complete at home afterwards.

20 participants (10 participants from each intervention condition) will be invited to provide feedback on their experiences of study participation, initial expectations of the intervention, perceived strengths and weaknesses, and suggestions for improvements in an interview with the researcher. Declining this invitation to interview does not affect study payment.

6 months after the baseline assessment, participants may be contacted by phone by a member of the King's College London Eating Disorders Unit for a brief phone call to

For peer review only - http://bmjopen.bmj.com/site/about/guidelines.xhtml

evaluate the presence of eating disorder symptoms. This 6 month check-in will allow any long-term therapeutic effects of the study treatment intervention to be evaluated. Participation in this phone call will not affect participant payment, which will have been administered earlier after the 1-month follow up assessment.

Expenses and payments
Upon completion of the study, all participants will be paid a maximum of £60 for completing each of the three assessment sessions (comprised of an in-person visit and at-home questionnaires); the baseline assessment, post-treatment assessment and follow-up assessment (£20 each). This payment should be declared for tax and/or benefit purposes. If you are assigned to an intervention condition involving 6 sessions of brain stimulation and computer training, you may also be compensated up to £10 per day for your travel expenses on these intervention session days.

What is expected from you as a participant?
We would expect you to complete all assessment sessions (pre-, post-treatment, follow-up), and if you are randomised to an intervention group, to attend all 6 (ABM + real/placebo tDCS) sessions as scheduled. We ask you to inform us immediately if for any reason you suddenly find yourself unable to attend a scheduled session.

Please let us know of any health problem that has developed, or any new diagnosis made since you enrolled for the study. Further, we would ask you to let us know of any new medication or change in medication whilst you are taking part in the study.

What are the possible disadvantages and risks of taking part? What are the side effects?
Combined brain stimulation and cognitive training sessions are time-consuming and may cause fatigue from concentrating on the task.

TDCS has been shown to be safe when used correctly in a clinical setting. However, you may find the procedure slightly uncomfortable. This is because a number of sensations can occur beneath the electrodes during stimulation including tingling, pain, itching, and burning. Not everyone feels these sensations or finds them uncomfortable, but if you do, remember you are free to stop the study at any point without giving an explanation. In some rarer cases, tDCS has been known to cause a headache, but this can be treated with mild painkillers (e.g. paracetamol). No side effects of ABM are known. We will assess any discomfort you may experience during intervention sessions throughout your involvement in the trial.

What are the possible benefits of taking part?
Unfortunately, there are no direct benefits to taking part in this study, but the information we get may help us to improve the treatment of BED in the future.

What happens when the research study stops?
When the research study stops, no further ABM + tDCS sessions will be available to those who have received 6 sessions. Participants in the control group will have the option of receiving 6 sessions of [ABM + real tDCS] once they have completed the waiting period and follow-up assessment.

For peer review only

Participants assigned to one of the intervention groups can request to be informed if they had received ABM combined with real or placebo tDCS, once they have completed the follow-up assessment.

What if there is a problem?
Any complaint about the way you have been dealt with during the study or any possible harm you might suffer will be addressed. Detailed information on this is given in Part 2.

Will my taking part in the study be kept confidential?
Yes. We will follow ethical and legal practice and all information about you will be handled in confidence. The details are included in Part 2.

If the information in Part 1 has interested you and you are considering participation, please read the additional information in Part 2 before making a decision.

PART TWO

What if relevant and new information becomes available?
Sometimes we get new information about the treatment being studied. This is not expected to occur given the short time frame of participation (6 sessions across 3 weeks); however, if any new and relevant information becomes available during this time we will inform you immediately. You can then decide whether you wish to continue in the study.

What will happen if I don't want to carry on with the study?
Your participation is voluntary and you do not have to take part in the study. You can change your mind at any point and terminate your participation without giving a reason to the researcher. You are free to withdraw from this study at any time without consequence.

Will participation in this study affect my routine healthcare, or the waiting period for treatment for my eating disorder if I am currently on a waiting list?
No. Participation in this study will have no impact on your treatment as usual, or waiting time if you are currently awaiting treatment for your eating disorder. We fully encourage you to begin treatment as provided by a health care professional as soon as it becomes available to you. We simply ask that you inform us of any changes to your treatment or medication while you are partaking in the study.

What if there is a problem?
If you have a concern about any aspect of the study, please ask the researcher (gemma.gordon@kcl.ac.uk, 0207 848 0183) who will do their best to answer your questions.

What if I wish to make a complaint?
If you remain unhappy and wish to formally complain, complaints to the IoPPN should be addressed to Dr Gill Dale. Director of Research Quality; Head, Joint R&D Office of South London and Maudsley NHS Foundation Trust and Institute of Psychiatry, Psychology & Neuroscience (IoPPN), P005, Institute of Psychiatry, Psychology &

For peer review only - http://bmjopen.bmj.com/site/about/guidelines.xhtml

Neuroscience (IoPPN), King's College London, De Crespigny Park, London SE5 8AF. NHS complaints will follow NHS complaints procedures.

Other sources of support for your eating disorder
To access support and treatment, please see your Eating Disorders clinician or your GP who will be able to advise you and refer you to the right service for you. You can also obtain further information and support from www.beateatingdisorders.org.uk, the national Eating Disorders charity.

Should you wish to speak to someone outside of the university, please talk to your Eating Disorders clinician, GP, the Beat helpline, and/or one of the study researchers who is happy to liaise on your behalf if you so wish. The eating disorders charity Beat provides helplines for adults and young people which offer support and information to sufferers, carers and professionals. Further information can be found on their website www.beateatingdisorders.org.uk, or by ringing their helpline 0808 801 0677**.**

Will my taking part in the study be kept confidential?
Your personal information and the data we collect from you will remain confidential at all times. It will also remain anonymous to everyone apart from the primary researchers. Manual files will be locked securely in a filing cabinet, which will be kept in a locked office in the KCL Section of Eating Disorders, Department of Psychological Medicine, IoPPN, and all electronic files will be password protected. All information which is collected during the course of the research will be kept strictly confidential according to the General Data Protection Regulation (GDPR), brought into effect on 25th May, 2018. This new legislation creates some new rights for individuals to better reflect data protection challenges in the modern digital age, as well as strengthening some of the rights that currently exist under the Data Protection Act 1998.

How will my personal data be used and what are my rights?
King's College London is the sponsor for this study based in the United Kingdom. We will be using information from you in order to undertake this study and will act as the data controller for this study. This means that we are responsible for looking after your information and using it properly. King's College London will keep identifiable information about you for four years after the study has finished.
Your rights to access, change or move your information are limited, as we need to manage your information in specific ways in order for the research to be reliable and accurate. If you withdraw from the study, we will keep the information about you that we have already obtained. To safeguard your rights, we will use the minimum personally-identifiable information possible. You can find out more about how we use your information by contacting the Study Coordinator Gemma Gordon.

KCL will use your name and contact details to contact you about the research study, and make sure that relevant information about the study is recorded for your care, and to oversee the quality of the study. Individuals from KCL and regulatory organisations may look at your medical and research records to check the accuracy of the research study. SLaM will pass these details to KCL along with the information collected from you. The only people in KCL who will have access to information that identifies you will be people who need to contact you regarding your participation or audit the data collection process. The people who analyse the information will not be able to identify

For peer review only - http://bmjopen.bmj.com/site/about/guidelines.xhtml

you and will not be able to find out your name or contact details. KCL will keep identifiable information about you from this study for four years after the study has finished.

Involvement of the General Practitioner (GP)
As a matter of courtesy and in the interest of your wellbeing, we may let your GP know about your participation in the study, and may request your permission to send them a letter when you enroll. If you agree to this, you will be asked to provide us with your GP's contact details so that we can send them a letter with details of the research.

Insurance/indemnity
Standard KCL insurance and NHS indemnity arrangements apply.

Involvement of the insurance company
If you have private medical insurance, you should inform your insurance company that you are taking part in this study.

Will any genetic tests be done?
No.

What will happen to the results of the research study?
You will be offered the opportunity to be informed about your individual results once the data for all participants has been collected. If you want written feedback of the study's findings you can contact the researcher (gemma.gordon@kcl.ac.uk) for a lay summary. The results will be included in an examined postgraduate report, presented as part of a postgraduate presentation, and sent to a medical journal for publication. Your participation in the study will not be disclosed.

Who is organising and funding the research?
This study is being funded by King's College London.

Who has reviewed the study?
All research in the NHS is looked at by an independent group of people, called a Research Ethics Committee, to protect your interests. This study has been reviewed and given favourable opinion by North West -Liverpool East Research Ethics Committee.

Further information and contact details
If you have any questions or require more information about this study, please contact the researcher using the following contact details:
Gemma Gordon (gemma.gordon@kcl.ac.uk) (0207 848 5608)
Section of Eating Disorders, Department of Psychological Medicine
KCL Institute of Psychiatry, Psychology and Neuroscience,
16 De Crespigny Park
London, SE5 8AF

For peer review only

SPIRIT

STANDARD PROTOCOL ITEMS: RECOMMENDATIONS FOR INTERVENTIONAL TRIALS

SPIRIT 2013 Checklist: Recommended items to address in a clinical trial protocol and related documents*

Section/item	Item No	Description	Addressed on page number
**Administrative information**			
Title	1	Descriptive title identifying the study design, population, interventions, and, if applicable, trial acronym	________1______
Trial registration	2a	Trial identifier and registry name. If not yet registered, name of intended registry	________1______
	2b	All items from the World Health Organization Trial Registration Data Set	______________
Protocol version	3	Date and version identifier	______1_______
Funding	4	Sources and types of financial, material, and other support	______12_______
Roles and responsibilities	5a	Names, affiliations, and roles of protocol contributors	______12______
	5b	Name and contact information for the trial sponsor	______12_______
	5c	Role of study sponsor and funders, if any, in study design; collection, management, analysis, and interpretation of data; writing of the report; and the decision to submit the report for publication, including whether they will have ultimate authority over any of these activities	______12______
	5d	Composition, roles, and responsibilities of the coordinating centre, steering committee, endpoint adjudication committee, data management team, and other individuals or groups overseeing the trial, if applicable (see Item 21a for data monitoring committee)	_____11____

For peer review only

For peer review only - http://bmjopen.bmj.com/site/about/guidelines.xhtml

Introduction

Background and rationale	6a	Description of research question and justification for undertaking the trial, including summary of relevant studies (published and unpublished) examining benefits and harms for each intervention	_____2-4___
	6b	Explanation for choice of comparators	______4__
Objectives	7	Specific objectives or hypotheses	______4___
Trial design	8	Description of trial design including type of trial (eg, parallel group, crossover, factorial, single group), allocation ratio, and framework (eg, superiority, equivalence, noninferiority, exploratory)	______5______

Methods: Participants, interventions, and outcomes

Study setting	9	Description of study settings (eg, community clinic, academic hospital) and list of countries where data will be collected. Reference to where list of study sites can be obtained	_____6_____
Eligibility criteria	10	Inclusion and exclusion criteria for participants. If applicable, eligibility criteria for study centres and individuals who will perform the interventions (eg, surgeons, psychotherapists)	______5_____
Interventions	11a	Interventions for each group with sufficient detail to allow replication, including how and when they will be administered	____9-10___
	11b	Criteria for discontinuing or modifying allocated interventions for a given trial participant (eg, drug dose change in response to harms, participant request, or improving/worsening disease)	____11___
	11c	Strategies to improve adherence to intervention protocols, and any procedures for monitoring adherence (eg, drug tablet return, laboratory tests)	____11____
	11d	Relevant concomitant care and interventions that are permitted or prohibited during the trial	____4, 11___
Outcomes	12	Primary, secondary, and other outcomes, including the specific measurement variable (eg, systolic blood pressure), analysis metric (eg, change from baseline, final value, time to event), method of aggregation (eg, median, proportion), and time point for each outcome. Explanation of the clinical relevance of chosen efficacy and harm outcomes is strongly recommended	____6-9___

For peer review only - http://bmjopen.bmj.com/site/about/guidelines.xhtml

Participant timeline	13	Time schedule of enrolment, interventions (including any run-ins and washouts), assessments, and visits for participants. A schematic diagram is highly recommended (see Figure)	___6__
Sample size	14	Estimated number of participants needed to achieve study objectives and how it was determined, including clinical and statistical assumptions supporting any sample size calculations	____5___
Recruitment	15	Strategies for achieving adequate participant enrolment to reach target sample size	___11__

Methods: Assignment of interventions (for controlled trials)

Allocation:

Sequence generation	16a	Method of generating the allocation sequence (eg, computer-generated random numbers), and list of any factors for stratification. To reduce predictability of a random sequence, details of any planned restriction (eg, blocking) should be provided in a separate document that is unavailable to those who enrol participants or assign interventions	____5-6___
Allocation concealment mechanism	16b	Mechanism of implementing the allocation sequence (eg, central telephone; sequentially numbered, opaque, sealed envelopes), describing any steps to conceal the sequence until interventions are assigned	____6___
Implementation	16c	Who will generate the allocation sequence, who will enrol participants, and who will assign participants to interventions	____5____
Blinding (masking)	17a	Who will be blinded after assignment to interventions (eg, trial participants, care providers, outcome assessors, data analysts), and how	___5____
	17b	If blinded, circumstances under which unblinding is permissible, and procedure for revealing a participant's allocated intervention during the trial	___6____

Methods: Data collection, management, and analysis

Data collection methods	18a	Plans for assessment and collection of outcome, baseline, and other trial data, including any related processes to promote data quality (eg, duplicate measurements, training of assessors) and a description of study instruments (eg, questionnaires, laboratory tests) along with their reliability and validity, if known. Reference to where data collection forms can be found, if not in the protocol	____6-8____

	18b	Plans to promote participant retention and complete follow-up, including list of any outcome data to be collected for participants who discontinue or deviate from intervention protocols	_____10-11____
Data management	19	Plans for data entry, coding, security, and storage, including any related processes to promote data quality (eg, double data entry; range checks for data values). Reference to where details of data management procedures can be found, if not in the protocol	_____10-11_____
Statistical methods	20a	Statistical methods for analysing primary and secondary outcomes. Reference to where other details of the statistical analysis plan can be found, if not in the protocol	_____10_____
	20b	Methods for any additional analyses (eg, subgroup and adjusted analyses)	_____10______
	20c	Definition of analysis population relating to protocol non-adherence (eg, as randomised analysis), and any statistical methods to handle missing data (eg, multiple imputation)	______10______
**Methods: Monitoring**			
Data monitoring	21a	Composition of data monitoring committee (DMC); summary of its role and reporting structure; statement of whether it is independent from the sponsor and competing interests; and reference to where further details about its charter can be found, if not in the protocol. Alternatively, an explanation of why a DMC is not needed	_____10_____
	21b	Description of any interim analyses and stopping guidelines, including who will have access to these interim results and make the final decision to terminate the trial	_____11______
Harms	22	Plans for collecting, assessing, reporting, and managing solicited and spontaneously reported adverse events and other unintended effects of trial interventions or trial conduct	_____11_____
Auditing	23	Frequency and procedures for auditing trial conduct, if any, and whether the process will be independent from investigators and the sponsor	_______n/a____
**Ethics and dissemination**			
Research ethics approval	24	Plans for seeking research ethics committee/institutional review board (REC/IRB) approval	______1, 13____

Protocol amendments	25	Plans for communicating important protocol modifications (eg, changes to eligibility criteria, outcomes, analyses) to relevant parties (eg, investigators, REC/IRBs, trial participants, trial registries, journals, regulators)	_____1, 12_____
Consent or assent	26a	Who will obtain informed consent or assent from potential trial participants or authorised surrogates, and how (see Item 32)	_____12_____
	26b	Additional consent provisions for collection and use of participant data and biological specimens in ancillary studies, if applicable	_____n/a_____
Confidentiality	27	How personal information about potential and enrolled participants will be collected, shared, and maintained in order to protect confidentiality before, during, and after the trial	____10-11____
Declaration of interests	28	Financial and other competing interests for principal investigators for the overall trial and each study site	_____12______
Access to data	29	Statement of who will have access to the final trial dataset, and disclosure of contractual agreements that limit such access for investigators	_____ 10_____
Ancillary and post-trial care	30	Provisions, if any, for ancillary and post-trial care, and for compensation to those who suffer harm from trial participation	______11_____
Dissemination policy	31a	Plans for investigators and sponsor to communicate trial results to participants, healthcare professionals, the public, and other relevant groups (eg, via publication, reporting in results databases, or other data sharing arrangements), including any publication restrictions	______1, 11_____
	31b	Authorship eligibility guidelines and any intended use of professional writers	______12______
	31c	Plans, if any, for granting public access to the full protocol, participant-level dataset, and statistical code	______n/a______
**Appendices**			
Informed consent materials	32	Model consent form and other related documentation given to participants and authorised surrogates	______24______
Biological specimens	33	Plans for collection, laboratory evaluation, and storage of biological specimens for genetic or molecular analysis in the current trial and for future use in ancillary studies, if applicable	______n/a______

For peer review only - http://bmjopen.bmj.com/site/about/guidelines.xhtml

*It is strongly recommended that this checklist be read in conjunction with the SPIRIT 2013 Explanation & Elaboration for important clarification on the items. Amendments to the protocol should be tracked and dated. The SPIRIT checklist is copyrighted by the SPIRIT Group under the Creative Commons "Attribution-NonCommercial-NoDerivs 3.0 Unported" license.

For peer review only

CONSORT 2010 checklist of information to include when reporting a randomised trial*

Section/Topic	Item No	Checklist item	Reported on page No
**Title and abstract**			
	1a	Identification as a randomised trial in the title	1
	1b	Structured summary of trial design, methods, results, and conclusions (for specific guidance see CONSORT for abstracts)	1(no results)
**Introduction**			
Background and objectives	2a	Scientific background and explanation of rationale	2-4
	2b	Specific objectives or hypotheses	4-5
**Methods**			
Trial design	3a	Description of trial design (such as parallel, factorial) including allocation ratio	5
	3b	Important changes to methods after trial commencement (such as eligibility criteria), with reasons	n/a
Participants	4a	Eligibility criteria for participants	5
	4b	Settings and locations where the data were collected	6
Interventions	5	The interventions for each group with sufficient details to allow replication, including how and when they were actually administered	9-10
Outcomes	6a	Completely defined pre-specified primary and secondary outcome measures, including how and when they were assessed	6-9
	6b	Any changes to trial outcomes after the trial commenced, with reasons	n/a
Sample size	7a	How sample size was determined	5
	7b	When applicable, explanation of any interim analyses and stopping guidelines	11
Randomisation:			
Sequence generation	8a	Method used to generate the random allocation sequence	5-6
	8b	Type of randomisation; details of any restriction (such as blocking and block size)	5-6
Allocation concealment mechanism	9	Mechanism used to implement the random allocation sequence (such as sequentially numbered containers), describing any steps taken to conceal the sequence until interventions were assigned	6
Implementation	10	Who generated the random allocation sequence, who enrolled participants, and who assigned participants to interventions	5
Blinding	11a	If done, who was blinded after assignment to interventions (for example, participants, care providers, those	5

For peer review only

For peer review only - http://bmjopen.bmj.com/site/about/guidelines.xhtml

		assessing outcomes) and how	
	11b	If relevant, description of the similarity of interventions	4
Statistical methods	12a	Statistical methods used to compare groups for primary and secondary outcomes	10
	12b	Methods for additional analyses, such as subgroup analyses and adjusted analyses	10
**Results**			
Participant flow (a diagram is strongly recommended)	13a	For each group, the numbers of participants who were randomly assigned, received intended treatment, and were analysed for the primary outcome	n/a
	13b	For each group, losses and exclusions after randomisation, together with reasons	n/a
Recruitment	14a	Dates defining the periods of recruitment and follow-up	n/a
	14b	Why the trial ended or was stopped	n/a
Baseline data	15	A table showing baseline demographic and clinical characteristics for each group	n/a
Numbers analysed	16	For each group, number of participants (denominator) included in each analysis and whether the analysis was by original assigned groups	n/a
Outcomes and estimation	17a	For each primary and secondary outcome, results for each group, and the estimated effect size and its precision (such as 95% confidence interval)	n/a
	17b	For binary outcomes, presentation of both absolute and relative effect sizes is recommended	n/a
Ancillary analyses	18	Results of any other analyses performed, including subgroup analyses and adjusted analyses, distinguishing pre-specified from exploratory	n/a
Harms	19	All important harms or unintended effects in each group (for specific guidance see CONSORT for harms)	n/a
**Discussion**			
Limitations	20	Trial limitations, addressing sources of potential bias, imprecision, and, if relevant, multiplicity of analyses	n/a
Generalisability	21	Generalisability (external validity, applicability) of the trial findings	n/a
Interpretation	22	Interpretation consistent with results, balancing benefits and harms, and considering other relevant evidence	n/a
**Other information**			
Registration	23	Registration number and name of trial registry	1
Protocol	24	Where the full trial protocol can be accessed, if available	n/a
Funding	25	Sources of funding and other support (such as supply of drugs), role of funders	12

*We strongly recommend reading this statement in conjunction with the CONSORT 2010 Explanation and Elaboration for important clarifications on all the items. If relevant, we also recommend reading CONSORT extensions for cluster randomised trials, non-inferiority and equivalence trials, non-pharmacological treatments, herbal interventions, and pragmatic trials. Additional extensions are forthcoming: for those and for up to date references relevant to this checklist, see www.consort-statement.org.

For peer review only - http://bmjopen.bmj.com/site/about/guidelines.xhtml

BMJ Open

Combining cognitive bias modification training (CBM) and transcranial direct current stimulation (tDCS) to treat binge eating disorder: study protocol of a randomised controlled feasibility trial

Journal:	*BMJ Open*
Manuscript ID	bmjopen-2019-030023.R1
Article Type:	Protocol
Date Submitted by the Author:	18-Jul-2019
Complete List of Authors:	Gordon, Gemma; King's College London, Section of Eating Disorders, Department of Psychological Medicine, Institute of Psychiatry, Psychology and Neuroscience,<br>Brockmeyer, Timo; University of Gottingen, Department of Clinical Psychology and Psychotherapy, Institute of Psychology<br>Schmidt, Ulrike; King's College London, Section of Eating Disorders, Department of Psychological Medicine, Institute of Psychiatry, Psychology and Neuroscience<br>Campbell, Iain; King's College London, Section of Eating Disorders, Department of Psychological Medicine, Institute of Psychiatry, Psychology and Neuroscience
**Primary Subject Heading**:	Mental health
Secondary Subject Heading:	Diabetes and endocrinology, Public health
Keywords:	Eating disorders < PSYCHIATRY, transcranial direct current stimulation, cognitive bias modification, binge eating, treatment

SCHOLARONE™
Manuscripts

07.07.2019 V2

Combining cognitive bias modification training (CBM) and transcranial direct current stimulation (tDCS) to treat binge eating disorder: study protocol of a randomised controlled feasibility trial

Gemma Gordon[1], Timo Brockmeyer[2], Ulrike Schmidt[1], Iain Campbell[1]

[1]Section of Eating Disorders, Department of Psychological Medicine, Institute of Psychiatry, Psychology and Neuroscience, King's College London, London, United Kingdom

[2]Department of Clinical Psychology and Psychotherapy, Institute of Psychology, University of Goettingen, Goettingen, Germany

Correspondence: Gemma Gordon, Section of Eating Disorders, Department of Psychological Medicine, Institute of Psychiatry, Psychology and Neuroscience, King's College London, London, United Kingdom; E-mail: gemma.gordon@kcl.ac.uk

Key words: cognitive bias modification, transcranial direct current stimulation, eating disorders, binge eating, treatment

Word count (abstract): 290 **Word count (manuscript): 5,908**

For peer review only

ABSTRACT

Introduction: Binge Eating Disorder (BED) is a common mental disorder, closely associated with obesity. Existing treatments are only moderately effective with high relapse rates, necessitating novel interventions. This paper describes the rationale for, and protocol of, a feasibility randomised controlled trial (RCT), evaluating the combination of transcranial Direct Current Stimulation (tDCS) and a computerised cognitive training, namely Approach Bias Modification training (ABM), in patients with Binge Eating Disorder who are overweight or obese. The aim of this trial is to obtain information that will guide decision making and protocol development in relation to a future large-scale RCT of combined tDCS + ABM treatment in this group of patients, and also to assess the preliminary efficacy of this intervention. **Methods and analysis:** 66 participants with DSM-5 diagnosis of BED and a body mass index (BMI) of >25 kg/m^2 will be randomly allocated to one of 3 groups: ABM + real tDCS; ABM + sham tDCS or a waitlist control group. Participants in both intervention groups will receive 6 sessions of ABM + real/sham tDCS over 3 weeks; engaging in the ABM task while simultaneously receiving bilateral tDCS to the dorsolateral prefrontal cortex. ABM is based on an implicit learning paradigm in which participants are trained to enact an avoidance behaviour in response to visual food cues. Assessments will be conducted at baseline, post-treatment (3 weeks), and follow-up (7 weeks post-randomisation). Feasibility outcomes assess recruitment and retention rates, acceptability of random allocation, blinding success (allocation concealment), completion of treatment sessions and research assessments. Other outcomes include eating disorder psychopathology and related neurocognitive outcomes (i.e. delay of gratification and inhibitory control), BMI, other psychopathology (i.e. mood), approach bias towards food, and surrogate endpoints (i.e. food cue reactivity, trait food craving, and food intake).

Ethics and dissemination: This study has been approved by the North West - Liverpool East Research Ethics Committee. Results will be published in peer-reviewed journals.

Trial registration number: ISRCTN35717198

Strengths and limitations of this study

- The ICARUS study is the first randomised controlled feasibility trial of multi-session transcranial Direct Current Stimulation (tDCS) combined with Cognitive Bias Modification training (CBM) for adults with Binge Eating Disorder.
- ICARUS will compare [tDCS + CBM] vs. [sham tDCS + CBM] and a wait-list control group.
- ICARUS is designed to answer questions about the efficacy of the treatments tested.
- Results would need to be replicated in a larger trial before recommendations for tDCS + CBM as a treatment adjunct for patients receiving outpatient treatment for BED can be made.

For peer review only - http://bmjopen.bmj.com/site/about/guidelines.xhtml

INTRODUCTION

Binge Eating Disorder (BED) is the most prevalent eating disorder (ED) worldwide, with 1-3% of the general population meeting diagnostic criteria[1,2]. Binge eating is a core symptom, characterised by consumption of large amounts of food, a sense of loss of control, and significant distress. Nearly 80% of those with lifetime BED have a comorbid psychiatric disorder, such as mood, anxiety, substance use disorders or another ED[2]. Due to the lack of compensatory behaviours (e.g. vomiting, excessive exercising), BED is often accompanied by, or leads to, obesity and associated physical complications[3,4]. In the general population, approximately 30-42% of people with BED are obese[2,5,6]. Around 30% of treatment-seeking obese people[7–9] and up to 47% of bariatric surgery candidates have full or partial BED[1,10,11]. Whilst BED itself has considerable individual and societal costs[12], the combination of BED and obesity is associated with more severe obesity, greater medical and psychiatric comorbidity, greater functional impairment and perinatal complications[12–15]. Treatments for BED and obesity are sub-optimally effective, with cognitive behavioural therapy (CBT)[16] and some medications[17] reducing binge eating and related psychopathology[1], and approximately 50-60% of patients achieving abstinence from bingeing at the end of treatment[18] with some sustained cessation at follow-up[19]. However, drop-out rates in established BED treatments reach 12-34%, and 30%-50% of BED patients relapse in long-term follow-ups[20–22], indicating that a substantial proportion do not maintain binge eating remission. Lisdexamfetamine[23] and topiramate[24] also reduce weight in the short-term but have considerable side effects[25], and their longer term efficacy is uncertain. Thus, there is a need for novel treatment developments.

The aetiology of BED is widely seen as multi-factorial. Emerging neurobiological models emphasise both the role of stress in the onset and maintenance of the disorder[26,27], and the development of addiction-like features; craving, tolerance and binge escalation over time[28,29], impulsivity and compulsivity, alterations in executive function and attention[30] and reward-related decision making[31].

Upon encountering images of high-calorie food, BED patients report enhanced reward sensitivity and exhibit stronger medial orbitofrontal cortex responses compared to healthy controls and participants with bulimia nervosa[32]. In individuals with obesity, who may or may not have BED, activation in the ventral striatum (part of the reward system) has been found to be higher compared to normal-weight controls [33], in tandem with a more pronounced approach bias towards appetising food images [34,35], leading to greater likelihood of consumption. Furthermore, poor reward-related decision making behaviour may be a maintaining factor in obesity[36]. Converging data using different methodologies, such as brain imaging, eye tracking, and behavioural test paradigms [37] have found that patients with BED demonstrate a higher arousal rate in response to food stimuli, a concurrent motor plan to start eating, a higher reward sensitivity, and greater inhibitory deficits as compared to individuals without BED[32,38,39]. Those with obesity and BED (compared to obesity alone) have demonstrated that their attentional bias to food images held higher motivational value[40], and responded more to high calorie food images in sites of cognitive planning of motor movements, driven by emotions, which may reflect impulsive tendencies in the face of a binge-eating trigger. This tendency to approach and consume palatable food items may thus be compounded by a greater sensitivity to reward and a decreased capacity to inhibit action tendencies. This is corroborated by the recent finding that individuals with BED or Bulimia Nervosa

For peer review only - http://bmjopen.bmj.com/site/about/guidelines.xhtml

(BN) show higher food cue reactivity (increased cravings) when exposed to visual food cues compared to healthy controls [41]. Such accumulating evidence of BED as a unique diagnostic group situates it as a distinct phenotype within the obesity spectrum that is characterised by increased impulsivity [42].

Conventional treatments of BED, such as CBT may not be best suited to target highly automatic cognitive processes that occur at an early stage in information processing and that are considered to contribute to food craving and associated maladaptive cognitions/behaviours. Two “brain-directed” treatments may provide an avenue for modifying these processes: Approach Bias Modification training (ABM) and transcranial Direct Current Stimulation (tDCS).

Approach Bias Modification training (ABM) is a form of cognitive bias modification training (CBM) that aims to retrain approach bias tendencies (reach out towards) into avoidance ones (move away from)[43] regarding stimuli such as appetitive cues. Participants are systematically trained to show an avoidance movement in response to illness-related rewarding stimuli (e.g. food or alcohol) on a computer screen. ABM techniques have shown potential in several pilot and large-scale randomised controlled studies to treat alcohol[44] and tobacco[45] addictions, and to reduce consumption of cannabis[46] and unhealthy foods[47,48]. ABM has also yielded promising results in people with high levels of food craving and in bulimic eating disorders, including BED[49,50]. However, mixed results in empirical studies across these domains[51,52] raise methodological issues of ABM studies to date, such as low statistical power and suboptimal choice (or absence) of control groups[53] and administration of single versus multiple training sessions.

Transcranial Direct Current Stimulation (tDCS) is a form of non-invasive brain stimulation (NIBS) that has been used as a treatment adjunct for a range of psychiatric disorders, such as depression, schizophrenia and addictions[54–56]. Preliminary evidence suggests that tDCS and other forms of non-invasive brain stimulation are promising tools to reduce food craving, ED symptoms and body weight in bulimic EDs, including BED, and obesity[57]. Additionally, some studies indicate that NIBS may reduce depression/stress levels and improve reward-based decision making in ED patients[58]. A frequent stimulation target is the dorsolateral prefrontal cortex (dlPFC) which plays a major role in cognitive-inhibition, emotion regulation, and reward processing[58–61]. Although precise mechanisms of action of tDCS have yet to be understood, a key hypothesis in relation to BED is that enhancing dlPFC activity via tDCS alters the reward-cognition balance towards facilitation of cognitive control and suppression of reward-related mechanisms driving food craving/overeating[62].

If given concurrently (i.e. ‘online training’), NIBS is reported to boost the effects of cognitive training on the reduction of cognitive biases and the improvement of response inhibition[63]. NIBS may enhance synaptic strength in neuronal pathways activated by cognitive training, amplifying effects of training, and thus cognitive bias modification efficacy [64]. As the effectiveness of tDCS may thus be improved by pairing administration with a cognitive task inducing activity in the target brain region[65–67], such combined treatment interventions have been investigated among alcohol dependent inpatients (ABM and tDCS)[68], and to enhance inhibitory control related to food consumption (Go/No-Go Task and tDCS)[65]. The insignificant findings from these studies warrant commentary that to date, studies that have found positive effects of tDCS have either included obese participants or have had multisession protocols[65,69–71]. As this study incorporates both

For peer review only - http://bmjopen.bmj.com/site/about/guidelines.xhtml

aspects, it is optimally designed to yield significant results.

In light of both the individual and societal burden incurred by the rising prevalence of BED and obesity, research interventions informing treatments that lead to stable and long-lasting remission are of critical importance, and novel therapies may play a role in serving as adjuncts to treatment as usual, to enhance improvement in clinical outcomes obtained from engaging with eating disorder treatment services. This research trial is the first to combine two promising novel intervention strategies in an integrated treatment and will yield important findings to shape future clinical trials. The intervention conditions of this feasibility study will involve 6 sessions of concurrent ABM and real or sham tDCS over 3 weeks, and will assess participant acceptability and dropout rates at this treatment frequency and duration. Additionally, the frequency of participants' ED symptoms and other outcomes related to general psychopathology and neurocognition will be measured before and after the study interventions to assess treatment success. In summary, this proof-of-concept and feasibility study will establish the utility of concurrent ABM + real tDCS in improving clinical outcomes in participants with BED, compared to ABM + sham tDCS, and a wait-list control group.

STUDY AIMS

In line with established recommendations for outcomes of feasibility trials[72], which at present are supported by the National Institute for Health Research, the primary aim is to assess the feasibility of using concurrent ABM + real tDCS compared to concurrent ABM + sham tDCS as a potential adjunct to treatment as usual (TAU) in this patient population, and acquire key information to inform the development of a large-scale randomised sham-controlled trial (RCT).

The specific objectives of the proposed feasibility study are to:

1. Establish the feasibility of conducting a large-scale RCT of ABM + tDCS in patients with BED by assessing recruitment, attendance, and retention rates;
2. Determine the practicality of administering both ABM and tDCS simultaneously;
3. Determine the best instruments for measuring outcomes in a full trial by examining the quality, completeness, and variability in the data;
4. Estimate the treatment effect sizes and standard deviations for outcome measures to inform the sample size calculation for a large-scale RCT;
5. Evaluate whether the treatment is operating as it is designed by analysing process measures, such as within-session visual analogue scales (VAS) of key ED symptoms;
6. Determine whether patients with BED evaluate concurrent ABM + tDCS as acceptable and credible;
7. Obtain information about patients' willingness to undergo random allocation to ABM paired with either real or sham tDCS administration, or the wait-list control condition.

A secondary aim is to investigate the potential efficacy of concurrent delivery of both forms of treatment on binge eating disorder.

This will involve evaluating if:

1. Concurrent sessions of ABM + real tDCS are superior to ABM + sham tDCS and to wait-list control in terms of frequency of objective binge eating episodes, food cue reactivity, food craving, food intake, eating disorder psychopathology and mood.

For peer review only - http://bmjopen.bmj.com/site/about/guidelines.xhtml

For peer review only

2. Concurrent ABM + tDCS is superior to the two other conditions in having an effect on the targeted neurocognitive mechanism (approach bias for high calorie food) and related neurocognitive parameters (i.e. impulsivity, delayed gratification, emotional regulation).
3. Concurrent ABM and sham tDCS is superior to the waitlist control in eliciting therapeutic effects on the aforementioned clinical outcomes and neurocognitive mechanisms, yet demonstrates an efficacy level below that of concurrent ABM and real tDCS.

METHODS AND ANALYSIS

This study protocol has been written according to the SPIRIT statement (Standard Protocol Items for Randomised Trials)[73] and the CONSORT 2010 statement (Consolidated Standards of Reporting Trials)[72].

Study design

The ICARUS trial (Investigating Concurrent Approach Bias Modification Training and Transcranial Direct Current Stimulation in Binge Eating Disorder) is an exploratory randomised controlled feasibility trial with three parallel treatment conditions; ABM + real tDCS, ABM + sham tDCS and wait-list control. All participants across the two intervention groups will receive a treatment protocol of 6 sessions of ABM + real/sham tDCS conducted over 3 weeks. The comparator groups of a wait-list control and ABM + sham tDCS are necessary to evaluate the potential effect of real versus sham tDCS in participants with BED. The wait-list control group will be examined at the same time points to control for the possibility that improvements in the intervention groups are simply due to regression to the mean, spontaneous remission or other non-specific time effects. Any participants who are engaging in treatment for their ED will continue with TAU, and thus this selection of comparators is deemed acceptable. Within treatment session measures will involve visual analogues scales evaluating mood, stress and eating disorder symptoms. Assessments will be conducted 3 times during the study; at baseline, post-treatment (week 3), and at follow-up (week 7).

Participants

Inclusion criteria entail: (1) male and female community-dwelling adults (aged 18-70); (2) overweight or obese according to WHO criteria (BMI>25 kg/m^2)[74]; (3) a diagnosis of full-syndrome or sub-threshold Binge Eating Disorder according to the DSM-5[75]L; (4) fluency in English; (5) normal or corrected to normal vision .
Exclusion criteria entail: (1) all known contraindications to tDCS[76]; (2) pregnancy; (3) a current significant/unstable medical or psychiatric disorder needing acute treatment in its own right; (4) a lifetime diagnosis of substance dependence, psychosis, bipolar disorder or borderline personality disorder; (5) taking psychotropic medication other than a stable dosage of selective serotonin reuptake inhibitors (SSRI) for at least 14 days prior to study enrolment; (6) allergies to any of the foods presented in the study; (7) smoking >10 cigarettes per day; (8) drinking >3-4 units (men) or 2-3 units (women) of alcohol per day. In line with the CONSORT guidelines[77,78], we will record the number and reasons for any participants we must exclude, or any who decline consent or withdraw from the study.

Sample size

For peer review only - http://bmjopen.bmj.com/site/about/guidelines.xhtml

As ICARUS is a feasibility study, an a priori sample size calculation is not necessary. Rather, its aim is to provide effect sizes on which future large-scale studies can be powered. Total sample sizes of n=24 to n=50 have been recommended for feasibility trials with a primary outcome measured on a continuous scale, mainly because estimates of the standard deviation for normally distributed variables tend to stabilise around this size[79,80]. We have chosen a target end sample size of n=60, (i.e. exceeds the upper end recommended for feasibility trials). However, assuming the attrition to follow-up rate is a = 0.10 (as found in previous eating disorder trials [81,82]) and applying an attrition correction factor of 1/(1-a), we will recruit an actual sample size of 66, i.e., 22 participants per group.

Randomisation

After the baseline assessment, participants will be allocated to one of three conditions at random to receive 6 sessions of either concurrent ABM + real tDCS or ABM + sham tDCS, or no intervention in the wait-list control condition. Participants in the wait-list control group will be offered the opportunity to receive ABM + real tDCS after the end of the follow-up. Participants will be individually randomised on a 1:1:1 ratio to the intervention or control groups in equal numbers. The generation and implementation of the randomisation sequence will be conducted independently from the trial team through a randomisation administrator who is not involved in any recruitment or research activity related to the ICARUS study. Online randomisation software (Sealed Envelope, London, UK) will be used for this purpose. Upon participant enrolment, the researcher will contact the randomisation administrator, who will inform this researcher in charge of carrying out the intervention of the participant's allocation via phone or email.

Blinding

Double blinding is implemented only for the intervention group cohorts of the trial. The research assessor will remain blind to each participant's tDCS assignment within the two intervention conditions until after the participant has completed the follow-up assessment. This double blinding protocol will be ensured via administration of the tDCS (NeuroConn DC-STIMULATOR PLUS) using "study mode". This involves a five-digit numerical code unique to each patient will be inputted into the device prior to the participant's testing session, that will initialise either sham or real (active) stimulation. The tDCS administrator and participants will remain blind to tDCS stimulation type throughout the study. Set-up of the randomisation codes and programming of the tDCS device will be performed by an investigator not involved in the trial. To assess blinding success, each participant and the researcher will be asked to guess the treatment allocation at the end of the 6 treatment sessions and to indicate how certain they are of this guess. The study group allocation will be revealed to the participant after their follow-up assessment. In the event of a reported change in a participant's medication, or a new clinical diagnosis made during their study participation, the early unblinding of study condition for an intervention group participant will be permissible. The trial database will be maintained 'blind' until the point of study data analyses.

Recruitment

The study will take place at the Institute of Psychiatry, Psychology and Neuroscience (IoPPN), King's College London (KCL), UK. Participants will be recruited from the Eating Disorders Service at the South London and Maudsley NHS Foundation Trust, from the KCL research recruitment webpage and social media account, and via posters placed on notice boards on KCL campuses. Participants who

For peer review only - http://bmjopen.bmj.com/site/about/guidelines.xhtml

have previously taken part in research at the KCL Eating Disorders Unit and who have consented to be informed of future studies may also be contacted. The ICARUS study will also be advertised on the Beat (National Eating Disorders Association) website, callforparticipants.com and www.mqmentalhealth.org. Potential participants will receive written and verbal study information and will be screened for eligibility. Eligible participants will provide informed written consent for study participation as a prerequisite for enrolment (See Appendices A and B).

Procedure

Figure 1 illustrates the timeline of the study procedures. All participants will partake in assessments at each of the three measurement points; baseline, post-treatment, and follow-up. Each assessment will comprise of an in-person study visit with tasks and measures, and online/hardcopy questionnaires and scales to be completed at home by the participant within 36 hours following the study visit. Table 1 details the tasks and measures allocated to each assessment and training visit. After the baseline assessment, participants are randomised to one of 3 groups; (1) ABM + real tDCS, (2) ABM + sham tDCS, or a waitlist control group (CG). Participants allocated to an intervention group will be offered 6 sessions of ABM + real/sham tDCS across 3 weeks. All study participants may receive TAU e.g. if they are currently engaged in outpatient treatment for their ED. The control group will not receive any study intervention, and any participants receiving outpatient services treatment for their ED will continue TAU during this 3 week period. The post-treatment assessment will be conducted on all participants after the 6th (final) session of ABM + real/sham tDCS for the intervention groups, and 3 weeks after the baseline assessment for the control group. The follow-up assessment will be conducted 28 days after the end of treatment, i.e. 7 weeks post-randomisation. A follow-up period is included because, if the effects of the intervention result from increased neuroplasticity, behavioural changes may need time to emerge. Assessing the longevity of favourable clinical outcomes beyond the treatment period is also relevant to the objectives of this feasibility study. The researcher conducting the assessment and testing sessions will remain blind to the study condition of intervention group participants until the follow-up has been completed.

Outcome assessment

Measures of feasibility, safety and adherence will be collected throughout the study. Outcomes related to ED symptoms, general psychopathology and neurocognition will be measured before and after the study intervention to assess treatment success. Each assessment session will be split into an in-person visit and at-home component to accommodate time constraints and minimise disruption to task performance due to participant fatigue. The in-person assessment measures will take approximately 75 minutes to complete, and the online questionnaires will take approximately 45 minutes.

Outcome Measures

Feasibility outcomes

As this is a feasibility study, an extensive range of outcome measures are included to help determine which are most sensitive to detecting a treatment effect. This will enable us to determine primary outcome(s) for a future large-scale RCT. However, based on previous research[49], the Eating Disorder Examination Questionnaire (EDE-Q) is anticipated to be a key outcome measure.

For peer review only - http://bmjopen.bmj.com/site/about/guidelines.xhtml

Intervention/service related outcomes

Feasibility outcomes include recruitment, attendance and retention rates, and acceptability of treatment by participants. Patients' acceptance of study interventions will be assessed by measuring treatment dropout rates and via the treatment tolerance and acceptability questionnaires. An interview assessment of treatment experience will be conducted with 20 participants after the follow-up is completed. 10 participants from each intervention group will be invited to provide feedback on their initial expectations and experiences of the ABM + real/sham tDCS treatments, perceived strengths and weaknesses of the treatment they received, and suggestions for improvements in procedures. Interviews will be recorded, transcribed and analysed using thematic analysis. This will allow future studies to consider patients' feedback in the development of research and clinical protocols of concurrent ABM and tDCS.

Clinical outcomes

Eating disorder and related psychopathology

(a) Eating Disorder Examination (EDE-Q)[83]: The EDE-Q is a widely used measure of eating-disordered behaviour and is widely regarded as the instrument of choice for the assessment of EDs. This will be administered at baseline, post-assessment and follow-up.

b) Body Mass Index (BMI (kg/m2)): This assessment of body composition provides accurate estimates of body fat percentages in adults, where sex and age are factored into the analysis measuring height and weight[84]. To calculate BMI, height and weight measurements will be obtained by the researcher at baseline, post-assessment and follow-up.

(c) *Approach bias assessment tasks*

To identify the most sensitive method of assessing change in approach bias towards high-calorie food items, two different computerised measures of approach bias will be used. In the Food Approach-Avoidance Task (F-AAT)[85] participants are shown colour photographs of high-calorie, palatable foods such as chocolate, cake, and pizza, and non-food household and office items such as sponges and stationary on a computer-screen[86]. They are instructed to approach and avoid these stimuli by moving a joystick toward themselves (approach) or away from themselves (avoidance). In the Stimulus Response Compatibility Task (SRC)[87], participants perform a symbolic movement by making a manikin image walk toward (approach) or away from stimuli (avoidance). See Appendix C for more detailed information. Both of these tasks will be administered at baseline, post-assessment and follow-up.

(d) Food choice attitudes/behaviour

The Food Choice Task[88–90] is a computer-based paradigm that measures responses to images of foods to assess food attitudes and characteristics of eating behaviour. Participants rate images of food on a computer screen according to healthiness as well as tastiness. Based on these ratings they are then offered a choice between a food that they consider "neutral" and a series of other foods. See Appendix C for more detailed information. This task will be performed at baseline and post-assessment.

For peer review only - http://bmjopen.bmj.com/site/about/guidelines.xhtml

(e) *Food craving after cue exposure task*
The Food Challenge Task (FCT)[41] will be used to examine cue-induced food craving. In this task, participants rate their state food craving using the Food Cravings Questionnaire State Version[91,92] before and after being presented with a video on a computer screen of foods shown to be highly appetising[93]. See Appendix C for more detailed information. This task will be performed at baseline and post-assessment.

(f) *Trait food craving*
Three questionnaires will be used to comprehensively assess mechanisms implicated in trait food craving. The Food Cravings Questionnaire Trait Version - reduced (FCQ-T-r)[94] is a 15 items only reduced version of a self-report questionnaire that measures trait levels of craving for food. The 21-item Power of Food Scale (PFS)[95] scale assesses the psychological influence of the mere presence or availability of food. It measures appetite for, rather than consumption of, palatable foods, at three levels of food proximity (food available, food present, and food tasted). The Yale Food Addiction Scale Version 2.0 (YFAS 2.0)[96] reflects the current diagnostic understanding of addiction to further investigate the potential role of an addictive process in problematic eating behaviour[75,97–101]. See Appendix C for more detailed information. Each of these scales will be administered at baseline, post-assessment and follow-up.

(g) *Food intake in a bogus taste test*
During the bogus taste test[102], participants will be instructed to rate and optionally consume highly palatable high-calorie food items presented in 3 bowls. See Appendix C for more detailed information. This task will be performed at baseline and post-assessment.

(h) *Increased preference for delayed rewards*
The Delay Discounting Task with Money and Food[103] examines whether small amounts of food would be discounted more steeply than money, as occurs with larger amounts. See Appendix C for more detailed information. This will be administered at baseline, post-assessment and follow-up.

(i) *Inhibitory control*
The cued Go/No-Go computer task is a classic test of executive function, requiring effortful response inhibition, and measures impulse control by the ability to inhibit instigated, prepotent responses. A food specific go/no-go task[104] measures impulsivity and response inhibition with respect to food and nonfood items. The Stop Signal Task (SST)[105]measures inhibitory control. Participants are required to engage in a computer task but withhold their response in the presence of a stop signal. An adaptation of the food version of the SST[106] will facilitate a comparison of responses between food and non-food categories. See Appendix C for more detailed information. Both of these tasks will be performed at baseline, post-assessment and follow-up.

Mood and emotion regulation

The Emotion Regulation Questionnaire (ERQ)[107] is designed to measure respondents' tendency to regulate their emotions regarding cognitive reappraisal and expressive suppression. The Positive and Negative Affect Schedule (PANAS)[108] measures the degree of positive or negative affect experienced "right now" in the current study. The Depression, Anxiety and Stress Scale (DASS-

For peer review only - http://bmjopen.bmj.com/site/about/guidelines.xhtml

21)[109] evaluates mood, anxiety and stress levels over the previous week. See Appendix C for more detailed information. All of these measures will be administered at baseline, post-assessment and follow-up.

Within session measures

Within each training session, i.e. immediately before and after the ABM + real/sham tDCS procedure the researcher will administer paper-based Visual Analogue Scales (VAS) assessing current hunger, feeling of fullness, urge to eat, urge to binge eat, feeling low, level of tension, level of stress, level of anxiety, and any discomfort due to tDCS and ABM in the training session. See Appendix C for more detailed information.

Intervention

In both intervention groups participants will receive six sessions of concurrent ABM and real or sham tDCS which will be delivered twice a week for three weeks. A researcher trained in tDCS administration will deliver the training sessions.

Rationale for number of sessions:

Treatment parameters for interventions of ABM and tDCS separately in psychiatric disorder research have not yet been standardised and vary from 1-12 sessions across a timeframe of days to multiple weeks. Mixed results regarding optimal frequency of ABM sessions and related forms of cognitive bias modification has been reported[110]. A maximum accumulative effect of modification efficacy at 6 sessions has been found for approach bias modification for alcohol dependence[44]. While there is a similar paucity of specifications for treatment parameters within tDCS, multisession NIBS interventions are significantly more effective at reducing cravings and strengthening the ability to refrain from food consumption than single session protocols in eating disorders and obesity[111]. As a single session of tDCS on patients with BED was found to reduce craving and caloric intake[59], it was hypothesised that repeated administration of tDCS would enhance this effect and may decrease binge eating frequency.

Within session safety procedures

The participant's blood pressure and heartrate will be taken by the researcher immediately before and after the session. While the participant is comfortably seated, the tDCS and ABM will be administered at the same time, i.e. participants will engage in ABM training whilst receiving tDCS. Each session will last 20 minutes. The ABM training will start 5 minutes after the start of the brain stimulation. ABM training will take place over 10 minutes and tDCS will then continue for a further 5 minutes. Participants will be reminded that they have the option to withdraw immediately and terminate their participation in the study if they experience discomfort during tDCS administration, or if they wish to withdraw for any reason that they may or may not wish to disclose.

Approach bias modification training

The ABM programme will use an implicit learning paradigm, based on a modified version of the Food Approach/Avoidance Task (AAT)[85,112–114]. In this task, participants are shown pictures of food and control (i.e. neutral office) items. They are required to pull (pictures grow bigger) or push (pictures grow smaller) a joystick in response to the outer frame of the picture (round vs.

For peer review only - http://bmjopen.bmj.com/site/about/guidelines.xhtml

For peer review only

rectangular), irrespective of the picture content. The training version of the Food-AAT utilises an implicit learning paradigm by presenting all food pictures in the "push" (i.e. avoid) format. The study procedure for ABM administration is aligned with previous research[50,115].

Transcranial direct current stimulation

TDCS (both real and sham) will be delivered using a NeuroConn® DC-STIMULATOR PLUS device at a constant current of 2 mA (with a 10-second fade in/out) using two 25cm² surface sponge electrodes soaked in a sterile saline solution (0.9% sodium chloride). The anode will be placed over the right dorsolateral prefrontal cortex (dlPFC) and the cathode over the left dlPFC. This montage has been used in sham-controlled studies on food craving, bulimia nervosa and BED[59]. The stimulation site will be located using the Beam F3 calculation method, which is based on the International 10-20 system. TDCS can occasionally result in mild discomfort during administration (i.e., tingling or itching sensation, a slightly metallic taste, occasional redness at the site of the electrodes). Fatigue, headache, nausea and insomnia have been reported as potential adverse reactions[116]. Participants who are at-risk for adverse effects[76] will be excluded from the study at the screening stage.

Data analysis

Data will be analysed with the Statistical Package of Social Sciences (SPSS). Feasibility outcome data will be analysed with appropriate summary statistics. To determine quality, completeness, and variability of the clinical outcome data, descriptive statistical analyses and graphical methods will be used. Intent-to-treat analyses will be performed. The size of the treatment effect on each outcome measure will be the difference in outcome data between those in the two treatment conditions and control condition at post-assessment and follow-up. Group differences will be estimated using linear mixed effects regression models, controlling for the baseline level of the outcome. The goal here is not to determine significant group differences but to establish a suitably precise effect size for the primary outcome at the post treatment assessment. This estimate will be used to guide the sample size of a future efficacy trial. Correlational analyses may be computed to analyse relationships between outcome variables and influences of potential covariates such as demographic variables, i.e. gender, age, BMI and clinical variables, i.e. start/stopping of psychotherapy, psychotropic medication and presence of comorbidities. Outcome data already obtained for participants who discontinue or deviate from the intervention protocol will be kept and analysed.

PATIENT AND PUBLIC INVOLVEMENT

Patients and/or public were not involved in the study design process, however we will obtain 20 intervention participants' qualitative views on their treatment experience in this study to inform future clinical trials.

ETHICS AND DISSEMINATION

Data management and data monitoring

Participant data will be anonymised and all anonymised data will be stored electronically on a password protected computer at the IoPPN. All trial data will be stored in line with the General Data Protection Regulation (GDPR) 2018. Hard copies of participant-related data (i.e. GP letters) will be

For peer review only - http://bmjopen.bmj.com/site/about/guidelines.xhtml

kept in locked cabinets at the IoPPN, King's College London. The final trial data set will not be accessed by anyone other than members of the research team.

Data will be stored on manual files, university and laptop computers. There will be no personal data stored on laptop computers. Confidentiality and anonymity of all personal data will be retained throughout the entire study. Manual files will be securely locked in a lockable filing cabinet, and all electronic files will be password protected. Identifying information will be removed from the data, stored separately and replaced with a numeric identification code. All participants will be allocated a numeric code, which will be used to identify their data. The master list of names which correspond to each participant's numeric identification code will be stored electronically and will be password protected. This information will only be accessible to key researchers involved in the study.

The online component of the assessment will use Online Surveys software (formerly BOS). King's College London uses this software for large scale surveys, and it is fully compliant with UK data protection laws. Participants will be emailed the link after the in-person component of each assessment session, and instructed to complete the second online component of the assessment within 36 hours. Participants will also have the option to receive and complete a hard copy version of the questionnaires with a stamped addressed envelope to post back to the study researcher at the IoPPN.

It is intended that the results of this feasibility study will be reported and disseminated at national and international conferences. Research findings may also be disseminated through internal newsletters and publications in collaboration with Beat, the UK's largest eating disorder charity.

Owing to the size and nature of this small-scale feasibility study, a data monitoring committee was not deemed to be required. There are no scheduled interim analyses and this trial may be prematurely discontinued by the Chief Investigator on the basis of new safety information.

Ethics and safety aspects

This study has been approved by the North West - Liverpool East Research Ethics Committee. This trial will be conducted in compliance with the study protocol, the Declaration of Helsinki, the principles of good clinical practice (ICH-E6 guideline), the ICH-E8 guideline and the principles of GCP and in accordance with all applicable regulatory requirements including but not limited to the UK policy framework for health and social care research. The ICARUS trial is registered with the International Standard Randomised Controlled Trial Number registry (number ISRCTN35717198). All participants will be asked by the study researcher to provide written informed consent prior to enrolment. Participants who are at-risk for adverse side effects[76]will be excluded from the study at the screening stage (i.e. such as those with pregnancy or epilepsy). Current safety parameters of tDCS administration regarding voltage amplitude and duration of brain stimulation sessions will be adhered to. Participants have the option to withdraw immediately and terminate their participation in the study if they experience discomfort during tDCS administration, or if they wish to terminate their participation for any other reason that they may or may not wish to disclose. After each training session, participants will complete the tolerance, discomfort and side effects questionnaire to report any adverse effects of the intervention training session. The researcher will record this description of any reported adverse effects, and record the severity and duration of symptoms and how the adverse effect was managed at the following training session. If a participant reports a new clinical diagnosis or change in medication during their involvement in the study, a decision regarding

For peer review only - http://bmjopen.bmj.com/site/about/guidelines.xhtml

their continued participation in the study will be made by the research team and withdrawal of the participant may be deemed necessary. Standard King's College London insurance and NHS indemnity arrangements apply to this study. To promote study adherence, upon completion of the follow-up assessment, each participant will be reimbursed for their time, efforts and travel (£60 for assessments and up to £60 for travel expenses). Additionally, participants in the wait-list control group will be offered the opportunity to receive 6 sessions of ABM + tDCS after the follow-up.

DISCUSSION

The ICARUS study represents the first feasibility study that aims to exploit a synergistic therapeutic effect by combining two brain-directed interventions in a single treatment intervention for BED. The rising clinical need of individuals with BED is currently met with few available psychological and neuropharmacological treatment options[117]. Therefore, such research advancing the identification and validation of novel therapies is greatly warranted.

This paper delineates the protocol for a feasibility trial which will inform future studies (i.e., provide effect sizes for a large RCT) and contribute to the extant research advocating brain-directed interventions for BED. The protocol aligns with current parameters of tDCS administration used to treat BED and BN[58,59] and utilises a multitudes of measures to identify the most appropriate and sensitive tools to detect treatment induced changes across pathological and neurocognitive domains.

Pragmatic concerns related to the recruitment process entail ensuring a sufficient and consistent rate of participant enrollment to meet the target sample number within the allocated timeframe. Additionally, drop-out rates for CBT treatment among a BED cohort are moderately high (17–30%)[118], thus study adherence will need to be monitored, with a revision of incentives/ reimbursement if necessary. Participants who were randomly allocated to the wait list control may avail themselves of 6 sessions of ABM + real tDCS after they have completed the study, which may promote recruitment and participant retention. Documenting the management of these issues will help to inform the development of a future large-scale RCT of this combined treatment adjunct for BED.

To conclude, investigating novel treatments for BED is an imperative issue. Combining ABM with tDCS is the strategic amalgamation of two techniques that have already demonstrated therapeutic efficacy in their own right. This feasibility RCT will be the first to systematically assess the acceptability and efficacy of a noninvasive, safe and potentially effective treatment adjunct to other therapies, which will enhance the ability of healthcare services to provide optimal care to patients with BED.

Trial progress

Recruitment will commence in February 2018 and data collection is expected to be complete (including follow-up assessments) by April 2020. Any substantial protocol amendments will be communicated to investigators via email and to other parties as required. Amendments to the study protocol will be reported in publications reporting the study outcomes.

For peer review only - http://bmjopen.bmj.com/site/about/guidelines.xhtml

Author affiliations

[1] Section of Eating Disorders, Department of Psychological Medicine, Institute of Psychiatry, Psychology & Neuroscience, King's College London, London, UK

[2] Department of Clinical Psychology and Psychotherapy, Institute of Psychology, University of Goettingen, Goettingen, Germany

Acknowledgements Not applicable

Contributors GG, IC, US and TB were each involved in the study conception and design. GG drafted the study protocol and IC, US and TB reviewed the paper and informed subsequent drafts. GG, IC, US and TB approved the final protocol paper.

Authorship policy No professional writers were involved in this study protocol, nor will be involved in the study report write-up.

Funding The project is supported by the NIHR Biomedical Research Centre (BRC) at South London and Maudsley NHS Foundation Trust (SLaM) and King's College London (KCL). Gemma Gordon is supported by a BRC PhD studentship and Ulrike Schmidt is supported by an NIHR Senior Investigator Award and receives salary support from the NIHR Mental Health BRC at SLaM NHS Foundation Trust and KCL. The funder was not involved in the study design and writing of this trial protocol paper, and will not be involved in the collection and analysis of data, nor the writing of the study report. The funders will not have ultimate authority over these activities.

Disclaimer The views expressed are those of the author(s) and not necessarily those of the NHS, the NIHR or the Department of Health.

Competing interests None declared

Patient consent Informed consent will be obtained by the researcher.

Ethics approval Ethical approval was given by the North West - Liverpool East Research Ethics Committee (ref: 18/NW/0648).

Provenance and peer review Not commissioned; externally peer reviewed.

Data sharing statement Not applicable

Open access This is an open access article distributed in accordance with the terms of the Creative Commons Attribution (CC BY 4.0) license, which permits others to distribute, remix, adapt and build upon this work, for commercial use, provided the original work is properly cited. See: http://creativecommons.org/ licenses/by/4.0/

© Article author(s) (or their employer(s) unless otherwise stated in the text of the article) 2018. All rights reserved. No commercial use is permitted unless otherwise expressly granted.

For peer review only

REFERENCES

1 McCuen-Wurst C, Ruggieri M, Allison KC. Disordered eating and obesity: associations between binge-eating disorder, night-eating syndrome, and weight-related comorbidities: Disordered eating and obesity. *Annals of the New York Academy of Sciences* 2018;**1411**:96–105. doi:10.1111/nyas.13467

2 Kessler RC, Berglund PA, Chiu WT, *et al.* The Prevalence and Correlates of Binge Eating Disorder in the World Health Organization World Mental Health Surveys. *Biological Psychiatry* 2013;**73**:904–14. doi:10.1016/j.biopsych.2012.11.020

3 de Zwaan M. Binge eating disorder and obesity. *Int J Obes Relat Metab Disord* 2001;**25 Suppl 1**:S51-55. doi:10.1038/sj.ijo.0801699

4 Hutson PH, Balodis IM, Potenza MN. Binge-eating disorder: Clinical and therapeutic advances. *Pharmacol Ther* 2018;**182**:15–27. doi:10.1016/j.pharmthera.2017.08.002

5 Cossrow N, Pawaskar M, Witt EA, *et al.* Estimating the Prevalence of Binge Eating Disorder in a Community Sample From the United States: Comparing DSM-IV-TR and DSM-5 Criteria. *J Clin Psychiatry* 2016;**77**:e968-974. doi:10.4088/JCP.15m10059

6 Kornstein SG, Kunovac JL, Herman BK, *et al.* Recognizing Binge-Eating Disorder in the Clinical Setting: A Review of the Literature. *Prim Care Companion CNS Disord* 2016;**18**. doi:10.4088/PCC.15r01905

7 Spitzer RL, Devlin M, Walsh BT, *et al.* Binge eating disorder: A multisite field trial of the diagnostic criteria. *International Journal of Eating Disorders* 1992;**11**:191–203. doi:10.1002/1098-108X(199204)11:3<191::AID-EAT2260110302>3.0.CO;2-S

8 Spitzer RL, Yanovski S, Wadden T, *et al.* Binge eating disorder: its further validation in a multisite study. *Int J Eat Disord* 1993;**13**:137–53.

9 Grucza RA, Przybeck TR, Cloninger CR. Prevalence and correlates of binge eating disorder in a community sample. *Comprehensive Psychiatry* 2007;**48**:124–31. doi:10.1016/j.comppsych.2006.08.002

10 Allison KC, Wadden TA, Sarwer DB, *et al.* Night eating syndrome and binge eating disorder among persons seeking bariatric surgery: prevalence and related features. *Obesity (Silver Spring)* 2006;**14 Suppl 2**:77S-82S. doi:10.1038/oby.2006.286

11 Adami GF, Gandolfo P, Bauer B, *et al.* Binge eating in massively obese patients undergoing bariatric surgery. *Int J Eat Disord* 1995;**17**:45–50.

12 Pawaskar M, Witt EA, Supina D, *et al.* Impact of binge eating disorder on functional impairment and work productivity in an adult community sample in the United States. *International Journal of Clinical Practice* 2017;**71**:e12970. doi:10.1111/ijcp.12970

13 Brown KL, LaRose JG, Mezuk B. The relationship between body mass index, binge eating disorder and suicidality. *BMC Psychiatry* 2018;**18**. doi:10.1186/s12888-018-1766-z

14 Watson HJ, Zerwas S, Torgersen L, *et al.* Maternal Eating Disorders and Perinatal Outcomes: A Three-Generation Study in the Norwegian Mother and Child Cohort Study. *J Abnorm Psychol* 2017;**126**:552–64. doi:10.1037/abn0000241

For peer review only - http://bmjopen.bmj.com/site/about/guidelines.xhtml

15 Raevuori A, Suokas J, Haukka J, *et al.* Highly increased risk of type 2 diabetes in patients with binge eating disorder and bulimia nervosa. *Int J Eat Disord* 2015;**48**:555–62. doi:10.1002/eat.22334

16 Palavras MA, Hay P, Filho CADS, *et al.* The Efficacy of Psychological Therapies in Reducing Weight and Binge Eating in People with Bulimia Nervosa and Binge Eating Disorder Who Are Overweight or Obese-A Critical Synthesis and Meta-Analyses. *Nutrients* 2017;**9**. doi:10.3390/nu9030299

17 Brownley KA, Berkman ND, Peat CM, *et al.* Binge-Eating Disorder in Adults: A Systematic Review and Meta-analysis. *Ann Intern Med* 2016;**165**:409–20. doi:10.7326/M15-2455

18 de Zwaan M, Herpertz S, Zipfel S, *et al.* Effect of Internet-Based Guided Self-help vs Individual Face-to-Face Treatment on Full or Subsyndromal Binge Eating Disorder in Overweight or Obese Patients: The INTERBED Randomized Clinical Trial. *JAMA Psychiatry* 2017;**74**:987–95. doi:10.1001/jamapsychiatry.2017.2150

19 Hilbert A, Bishop ME, Stein RI, *et al.* Long-term efficacy of psychological treatments for binge eating disorder. *Br J Psychiatry* 2012;**200**:232–7. doi:10.1192/bjp.bp.110.089664

20 Brownley KA, Berkman ND, Sedway JA, *et al.* Binge eating disorder treatment: a systematic review of randomized controlled trials. *Int J Eat Disord* 2007;**40**:337–48. doi:10.1002/eat.20370

21 Vocks S, Tuschen-Caffier B, Pietrowsky R, *et al.* Meta-analysis of the effectiveness of psychological and pharmacological treatments for binge eating disorder. *Int J Eat Disord* 2010;**43**:205–17. doi:10.1002/eat.20696

22 Wilson GT, Wilfley DE, Agras WS, *et al.* Psychological treatments of binge eating disorder. *Arch Gen Psychiatry* 2010;**67**:94–101. doi:10.1001/archgenpsychiatry.2009.170

23 McElroy SL, Hudson J, Ferreira-Cornwell MC, *et al.* Lisdexamfetamine Dimesylate for Adults with Moderate to Severe Binge Eating Disorder: Results of Two Pivotal Phase 3 Randomized Controlled Trials. *Neuropsychopharmacology* 2016;**41**:1251–60. doi:10.1038/npp.2015.275

24 Guerdjikova AI, Fitch A, McElroy SL. Successful Treatment of Binge Eating Disorder With Combination Phentermine/Topiramate Extended Release. *Prim Care Companion CNS Disord* 2015;**17**. doi:10.4088/PCC.14l01708

25 Arif H, Buchsbaum R, Weintraub D, *et al.* Patient-reported cognitive side effects of antiepileptic drugs: Predictors and comparison of all commonly used antiepileptic drugs. *Epilepsy & Behavior* 2009;**14**:202–9. doi:10.1016/j.yebeh.2008.10.017

26 Razzoli M, Pearson C, Crow S, *et al.* Stress, overeating, and obesity: Insights from human studies and preclinical models. *Neurosci Biobehav Rev* 2017;**76**:154–62. doi:10.1016/j.neubiorev.2017.01.026

27 Vannucci A, Nelson EE, Bongiorno DM, *et al.* Behavioral and neurodevelopmental precursors to binge-type eating disorders: support for the role of negative valence systems. *Psychological Medicine* 2015;**45**:2921–36. doi:10.1017/S003329171500104X

28 Smith DG, Robbins TW. The neurobiological underpinnings of obesity and binge eating: a rationale for adopting the food addiction model. *Biol Psychiatry* 2013;**73**:804–10. doi:10.1016/j.biopsych.2012.08.026

For peer review only - http://bmjopen.bmj.com/site/about/guidelines.xhtml

29 Devlin M. Binge Eating Disorder. In: *In K.D Brownell & B.T. Walsh (Eds.), Eating Disorders and Obesity, A Comprehemsive Handbook*. New York: : The Guildford Press 2017. 192–7.

30 Kessler RM, Hutson PH, Herman BK, *et al.* The neurobiological basis of binge-eating disorder. *Neuroscience & Biobehavioral Reviews* 2016;**63**:223–38. doi:10.1016/j.neubiorev.2016.01.013

31 Wu M, Brockmeyer T, Hartmann M, *et al.* Reward-related decision making in eating and weight disorders: A systematic review and meta-analysis of the evidence from neuropsychological studies. *Neurosci Biobehav Rev* 2016;**61**:177–96. doi:10.1016/j.neubiorev.2015.11.017

32 Schienle A, Schäfer A, Hermann A, *et al.* Binge-eating disorder: reward sensitivity and brain activation to images of food. *Biol Psychiatry* 2009;**65**:654–61. doi:10.1016/j.biopsych.2008.09.028

33 Stice E, Spoor S, Ng J, *et al.* Relation of obesity to consummatory and anticipatory food reward. *Physiol Behav* 2009;**97**:551–60. doi:10.1016/j.physbeh.2009.03.020

34 Brignell C, Griffiths T, Bradley BP, *et al.* Attentional and approach biases for pictorial food cues. Influence of external eating. *Appetite* 2009;**52**:299–306. doi:10.1016/j.appet.2008.10.007

35 Mehl N, Mueller-Wieland L, Mathar D, *et al.* Retraining automatic action tendencies in obesity. *Physiol Behav* 2018;**192**:50–8. doi:10.1016/j.physbeh.2018.03.031

36 Brockmeyer T, Simon JJ, Becker A, *et al.* Reward-related decision making and long-term weight loss maintenance. *Physiol Behav* 2017;**181**:69–74. doi:10.1016/j.physbeh.2017.09.008

37 Paslakis G, Kühn S, Grunert S, *et al.* Explicit and Implicit Approach vs. Avoidance Tendencies towards High vs. Low Calorie Food Cues in Patients with Obesity and Active Binge Eating Disorder. *Nutrients* 2017;**9**. doi:10.3390/nu9101068

38 Schag K, Teufel M, Junne F, *et al.* Impulsivity in binge eating disorder: food cues elicit increased reward responses and disinhibition. *PLoS ONE* 2013;**8**:e76542. doi:10.1371/journal.pone.0076542

39 Svaldi J, Naumann E, Trentowska M, *et al.* General and food-specific inhibitory deficits in binge eating disorder: Behavioral Inhibition in Bed. *International Journal of Eating Disorders* 2014;**47**:534–42. doi:10.1002/eat.22260

40 Aviram-Friedman R, Astbury N, Ochner CN, *et al.* Neurobiological evidence for attention bias to food, emotional dysregulation, disinhibition and deficient somatosensory awareness in obesity with binge eating disorder. *Physiol Behav* 2018;**184**:122–8. doi:10.1016/j.physbeh.2017.11.003

41 Meule A, Küppers C, Harms L, *et al.* Food cue-induced craving in individuals with bulimia nervosa and binge-eating disorder. *PLoS ONE* 2018;**13**:e0204151. doi:10.1371/journal.pone.0204151

42 Giel KE, Teufel M, Junne F, *et al.* Food-Related Impulsivity in Obesity and Binge Eating Disorder-A Systematic Update of the Evidence. *Nutrients* 2017;**9**. doi:10.3390/nu9111170

43 Kakoschke N, Kemps E, Tiggemann M. Approach bias modification training and consumption: A review of the literature. *Addict Behav* 2017;**64**:21–8. doi:10.1016/j.addbeh.2016.08.007

For peer review only - http://bmjopen.bmj.com/site/about/guidelines.xhtml

44 Eberl C, Wiers RW, Pawelczack S, *et al.* Implementation of approach bias re-training in alcoholism-how many sessions are needed? *Alcohol Clin Exp Res* 2014;**38**:587–94. doi:10.1111/acer.12281

45 Wittekind CE, Feist A, Schneider BC, *et al.* The approach-avoidance task as an online intervention in cigarette smoking: a pilot study. *J Behav Ther Exp Psychiatry* 2015;**46**:115–20. doi:10.1016/j.jbtep.2014.08.006

46 Sherman BJ, Baker NL, Squeglia LM, *et al.* Approach bias modification for cannabis use disorder: A proof-of-principle study. *Journal of Substance Abuse Treatment* 2018;**87**:16–22. doi:10.1016/j.jsat.2018.01.012

47 Schumacher SE, Kemps E, Tiggemann M. Bias modification training can alter approach bias and chocolate consumption. *Appetite* 2016;**96**:219–24. doi:10.1016/j.appet.2015.09.014

48 Warschburger P, Gmeiner M, Morawietz M, *et al.* Evaluation of an approach-avoidance training intervention for children and adolescents with obesity: A randomized placebo-controlled prospective trial. *Eur Eat Disord Rev* 2018;**26**:472–82. doi:10.1002/erv.2607

49 Brockmeyer T, Hahn C, Reetz C, *et al.* Approach Bias Modification in Food Craving-A Proof-of-Concept Study. *Eur Eat Disord Rev* 2015;**23**:352–60. doi:10.1002/erv.2382

50 Brockmeyer T, Friederich H-C, Küppers C, *et al.* Approach bias modification training in bulimia nervosa and binge-eating disorder: A pilot randomized controlled trial. *Int J Eat Disord* Published Online First: 28 January 2019. doi:10.1002/eat.23024

51 Becker D, Jostmann NB, Wiers RW, *et al.* Approach avoidance training in the eating domain: testing the effectiveness across three single session studies. *Appetite* 2015;**85**:58–65. doi:10.1016/j.appet.2014.11.017

52 Lindgren KP, Wiers RW, Teachman BA, *et al.* Attempted Training of Alcohol Approach and Drinking Identity Associations in US Undergraduate Drinkers: Null Results from Two Studies. *PLoS ONE* 2015;**10**:e0134642. doi:10.1371/journal.pone.0134642

53 Becker D, Jostmann NB, Holland RW. Does approach bias modification really work in the eating domain? A commentary on Kakoschke et al. (2017). *Addict Behav* 2018;**77**:293–4. doi:10.1016/j.addbeh.2017.02.025

54 Brunoni AR, Moffa AH, Sampaio-Junior B, *et al.* Trial of Electrical Direct-Current Therapy versus Escitalopram for Depression. http://dx.doi.org/10.1056/NEJMoa1612999. 2017. doi:10.1056/NEJMoa1612999

55 Bose A, Shivakumar V, Agarwal SM, *et al.* Efficacy of fronto-temporal transcranial direct current stimulation for refractory auditory verbal hallucinations in schizophrenia: A randomized, double-blind, sham-controlled study. *Schizophrenia Research* 2018;**195**:475–80. doi:10.1016/j.schres.2017.08.047

56 Lupi M, Martinotti G, Santacroce R, *et al.* Transcranial Direct Current Stimulation in Substance Use Disorders: A Systematic Review of Scientific Literature. *J ECT* 2017;**33**:203–9. doi:10.1097/YCT.0000000000000401

For peer review only - http://bmjopen.bmj.com/site/about/guidelines.xhtml

57 Dalton B, Campbell IC, Schmidt U. Neuromodulation and neurofeedback treatments in eating disorders and obesity. *Curr Opin Psychiatry* 2017;**30**:458–73. doi:10.1097/YCO.0000000000000361

58 Kekic M, McClelland J, Campbell I, *et al.* The effects of prefrontal cortex transcranial direct current stimulation (tDCS) on food craving and temporal discounting in women with frequent food cravings. *Appetite* 2014;**78**:55–62. doi:10.1016/j.appet.2014.03.010

59 Burgess EE, Sylvester MD, Morse KE, *et al.* Effects of transcranial direct current stimulation (tDCS) on binge eating disorder. *Int J Eat Disord* 2016;**49**:930–6. doi:10.1002/eat.22554

60 Fregni F, Orsati F, Pedrosa W, *et al.* Transcranial direct current stimulation of the prefrontal cortex modulates the desire for specific foods. *Appetite* 2008;**51**:34–41. doi:10.1016/j.appet.2007.09.016

61 Goldman RL, Borckardt JJ, Frohman HA, *et al.* Prefrontal cortex transcranial direct current stimulation (tDCS) temporarily reduces food cravings and increases the self-reported ability to resist food in adults with frequent food craving. *Appetite* 2011;**56**:741–6. doi:10.1016/j.appet.2011.02.013

62 Val-Laillet D, Aarts E, Weber B, *et al.* Neuroimaging and neuromodulation approaches to study eating behavior and prevent and treat eating disorders and obesity. *Neuroimage Clin* 2015;**8**:1–31. doi:10.1016/j.nicl.2015.03.016

63 Heeren A, Baeken C, Vanderhasselt M-A, *et al.* Impact of Anodal and Cathodal Transcranial Direct Current Stimulation over the Left Dorsolateral Prefrontal Cortex during Attention Bias Modification: An Eye-Tracking Study. *PLoS ONE* 2015;**10**:e0124182. doi:10.1371/journal.pone.0124182

64 Fritsch B, Reis J, Martinowich K, *et al.* Direct current stimulation promotes BDNF-dependent synaptic plasticity: potential implications for motor learning. *Neuron* 2010;**66**:198–204. doi:10.1016/j.neuron.2010.03.035

65 Sedgmond J, Lawrence NS, Verbruggen F, *et al.* Prefrontal brain stimulation during food-related inhibition training: effects on food craving, food consumption and inhibitory control. *R Soc Open Sci* 2019;**6**:181186. doi:10.1098/rsos.181186

66 Antal A, Terney D, Poreisz C, *et al.* Towards unravelling task-related modulations of neuroplastic changes induced in the human motor cortex. *Eur J Neurosci* 2007;**26**:2687–91. doi:10.1111/j.1460-9568.2007.05896.x

67 Wiers RW, Gladwin TE, Hofmann W, *et al.* Cognitive Bias Modification and Cognitive Control Training in Addiction and Related Psychopathology: Mechanisms, Clinical Perspectives, and Ways Forward. *Clinical Psychological Science* 2013;**1**:192–212. doi:10.1177/2167702612466547

68 den Uyl TE, Gladwin TE, Rinck M, *et al.* A clinical trial with combined transcranial direct current stimulation and alcohol approach bias retraining. *Addict Biol* 2017;**22**:1632–40. doi:10.1111/adb.12463

69 Ray MK, Sylvester MD, Osborn L, *et al.* The critical role of cognitive-based trait differences in transcranial direct current stimulation (tDCS) suppression of food craving and eating in frank obesity. *Appetite* 2017;**116**:568–74. doi:10.1016/j.appet.2017.05.046

For peer review only - http://bmjopen.bmj.com/site/about/guidelines.xhtml

70 Gluck ME, Alonso-Alonso M, Piaggi P, *et al.* Noninvasive neuromodulation targeted to the lateral prefrontal cortex induces changes in energy intake and weight loss in obesity. *Obesity (Silver Spring)* 2015;**23**:2149–56. doi:10.1002/oby.21313

71 Jauch-Chara K, Kistenmacher A, Herzog N, *et al.* Repetitive electric brain stimulation reduces food intake in humans. *Am J Clin Nutr* 2014;**100**:1003–9. doi:10.3945/ajcn.113.075481

72 Eldridge SM, Chan CL, Campbell MJ, *et al.* CONSORT 2010 statement: extension to randomised pilot and feasibility trials. *BMJ* 2016;**355**:i5239. doi:10.1136/bmj.i5239

73 Chan A-W, Tetzlaff JM, Altman DG, *et al.* SPIRIT 2013 Statement: defining standard protocol items for clinical trials. *Rev Panam Salud Publica* 2015;**38**:506–14.

74 Physical status: the use and interpretation of anthropometry. Report of a WHO Expert Committee. *World Health Organ Tech Rep Ser* 1995;**854**:1–452.

75 Association AP. *Diagnostic and Statistical Manual of Mental Disorders (DSM-5®)*. American Psychiatric Pub 2013.

76 Brunoni AR, Nitsche MA, Bolognini N, *et al.* Clinical research with transcranial direct current stimulation (tDCS): challenges and future directions. *Brain Stimul* 2012;**5**:175–95. doi:10.1016/j.brs.2011.03.002

77 Moher D, Schulz KF, Altman DG. The CONSORT statement: revised recommendations for improving the quality of reports of parallel-group randomised trials. *Lancet* 2001;**357**:1191–4.

78 Schulz KF, Altman DG, Moher D, *et al.* CONSORT 2010 statement: updated guidelines for reporting parallel group randomized trials. *Ann Intern Med* 2010;**152**:726–32. doi:10.7326/0003-4819-152-11-201006010-00232

79 Lancaster GA, Dodd S, Williamson PR. Design and analysis of pilot studies: recommendations for good practice. *J Eval Clin Pract* 2004;**10**:307–12. doi:10.1111/j.2002.384.doc.x

80 Julious SA. Sample size of 12 per group rule of thumb for a pilot study. *Pharmaceutical Statistics* 2005;**4**:287–91. doi:10.1002/pst.185

81 Schmidt U, Oldershaw A, Jichi F, *et al.* Out-patient psychological therapies for adults with anorexia nervosa: randomised controlled trial. *Br J Psychiatry* 2012;**201**:392–9. doi:10.1192/bjp.bp.112.112078

82 Schmidt U, Renwick B, Lose A, *et al.* The MOSAIC study - comparison of the Maudsley Model of Treatment for Adults with Anorexia Nervosa (MANTRA) with Specialist Supportive Clinical Management (SSCM) in outpatients with anorexia nervosa or eating disorder not otherwise specified, anorexia nervosa type: study protocol for a randomized controlled trial. *Trials* 2013;**14**:160. doi:10.1186/1745-6215-14-160

83 Fairburn CG, Beglin S, editors. *Cognitive Behavior Therapy and Eating Disorders.* New York: Guildford Press 2008.

84 Deurenberg P, Weststrate JA, Seidell JC. Body mass index as a measure of body fatness: age- and sex-specific prediction formulas. *British Journal of Nutrition* 1991;**65**:105–14. doi:10.1079/BJN19910073

For peer review only

For peer review only - http://bmjopen.bmj.com/site/about/guidelines.xhtml

85 Rinck M, Becker ES. Approach and avoidance in fear of spiders. *J Behav Ther Exp Psychiatry* 2007;**38**:105–20. doi:10.1016/j.jbtep.2006.10.001

86 Blechert J, Meule A, Busch NA, *et al.* Food-pics: an image database for experimental research on eating and appetite. *Front Psychol* 2014;**5**:617. doi:10.3389/fpsyg.2014.00617

87 De Houwer J, Crombez G, Baeyens F, *et al.* On the generality of the affective Simon effect. *Cognition & Emotion* 2001;**15**:189–206. doi:10.1080/02699930125883

88 Steinglass J, Foerde K, Kostro K, *et al.* Restrictive food intake as a choice--a paradigm for study. *Int J Eat Disord* 2015;**48**:59–66. doi:10.1002/eat.22345

89 Foerde K, Gianini L, Wang Y, *et al.* Assessment of test-retest reliability of a food choice task among healthy individuals. *Appetite* 2018;**123**:352–6. doi:10.1016/j.appet.2018.01.010

90 Hare TA, Camerer CF, Rangel A. Self-control in decision-making involves modulation of the vmPFC valuation system. *Science* 2009;**324**:646–8. doi:10.1126/science.1168450

91 Cepeda-Benito A, Gleaves DH, Williams TL, *et al.* The development and validation of the state and trait food-cravings questionnaires. *Behavior Therapy* 2000;**31**:151–73. doi:10.1016/S0005-7894(00)80009-X

92 Meule A, Lutz A, Vögele C, *et al.* Food cravings discriminate differentially between successful and unsuccessful dieters and non-dieters. Validation of the Food Cravings Questionnaires in German. *Appetite* 2012;**58**:88–97. doi:10.1016/j.appet.2011.09.010

93 Kekic M, McClelland J, Bartholdy S, *et al.* Single-Session Transcranial Direct Current Stimulation Temporarily Improves Symptoms, Mood, and Self-Regulatory Control in Bulimia Nervosa: A Randomised Controlled Trial. *PLoS ONE* 2017;**12**:e0167606. doi:10.1371/journal.pone.0167606

94 Meule A, Hermann T, Kübler A. A short version of the Food Cravings Questionnaire-Trait: the FCQ-T-reduced. *Front Psychol* 2014;**5**:190. doi:10.3389/fpsyg.2014.00190

95 Lowe MR, Butryn ML, Didie ER, *et al.* The Power of Food Scale. A new measure of the psychological influence of the food environment. *Appetite* 2009;**53**:114–8. doi:10.1016/j.appet.2009.05.016

96 Gearhardt AN, Corbin WR, Brownell KD. Development of the Yale Food Addiction Scale Version 2.0. *Psychol Addict Behav* 2016;**30**:113–21. doi:10.1037/adb0000136

97 Gearhardt AN, White MA, Masheb RM, *et al.* An examination of food addiction in a racially diverse sample of obese patients with binge eating disorder in primary care settings. *Compr Psychiatry* 2013;**54**:500–5. doi:10.1016/j.comppsych.2012.12.009

98 Steward T, Mestre-Bach G, Vintró-Alcaraz C, *et al.* Food addiction and impaired executive functions in women with obesity. *European Eating Disorders Review* 2018;**26**:574–84. doi:10.1002/erv.2636

99 Meule A, Heckel D, Kübler A. Factor Structure and Item Analysis of the Yale Food Addiction Scale in Obese Candidates for Bariatric Surgery. *European Eating Disorders Review* 2012;**20**:419–22. doi:10.1002/erv.2189

For peer review only - http://bmjopen.bmj.com/site/about/guidelines.xhtml

100 Clark SM, Saules KK. Validation of the Yale Food Addiction Scale among a weight-loss surgery population. *Eating Behaviors* 2013;**14**:216–9. doi:10.1016/j.eatbeh.2013.01.002

101 Carter JC, Van Wijk M, Rowsell M. Symptoms of ‘food addiction’ in binge eating disorder using the Yale Food Addiction Scale version 2.0. *Appetite* 2019;**133**:362–9. doi:10.1016/j.appet.2018.11.032

102 Robinson E, Haynes A, Hardman CA, *et al.* The bogus taste test: Validity as a measure of laboratory food intake. *Appetite* 2017;**116**:223–31. doi:10.1016/j.appet.2017.05.002

103 Odum AL, Baumann AAL, Rimington DD. Discounting of delayed hypothetical money and food: effects of amount. *Behav Processes* 2006;**73**:278–84. doi:10.1016/j.beproc.2006.06.008

104 Teslovich T, Freidl EK, Kostro K, *et al.* Probing behavioral responses to food: development of a food-specific go/no-go task. *Psychiatry Res* 2014;**219**:166–70. doi:10.1016/j.psychres.2014.04.053

105 Verbruggen F, Logan GD. Response inhibition in the stop-signal paradigm. *Trends Cogn Sci (Regul Ed)* 2008;**12**:418–24. doi:10.1016/j.tics.2008.07.005

106 Manasse SM, Goldstein SP, Wyckoff E, *et al.* Slowing down and taking a second look: Inhibitory deficits associated with binge eating are not food-specific. *Appetite* 2016;**96**:555–9. doi:10.1016/j.appet.2015.10.025

107 Gross JJ, John OP. Individual differences in two emotion regulation processes: implications for affect, relationships, and well-being. *J Pers Soc Psychol* 2003;**85**:348–62.

108 Watson D, Clark LA, Tellegen A. Development and validation of brief measures of positive and negative affect: the PANAS scales. *J Pers Soc Psychol* 1988;**54**:1063–70.

109 Lovibond SH, Lovibond PF. *Manual for the Depression Anxiety Stress Scales (2nd. ed.)*. Sydney Psychology Foundation 1995.

110 Jones EB, Sharpe L. Cognitive bias modification: A review of meta-analyses. *J Affect Disord* 2017;**223**:175–83. doi:10.1016/j.jad.2017.07.034

111 Song S, Zilverstand A, Gui W, *et al.* Effects of single-session versus multi-session non-invasive brain stimulation on craving and consumption in individuals with drug addiction, eating disorders or obesity: A meta-analysis. *Brain Stimul* Published Online First: 27 December 2018. doi:10.1016/j.brs.2018.12.975

112 Neumann R, Strack F. Approach and avoidance: the influence of proprioceptive and exteroceptive cues on encoding of affective information. *J Pers Soc Psychol* 2000;**79**:39–48.

113 Vrijsen JN, Fischer VS, Müller BW, *et al.* Cognitive bias modification as an add-on treatment in clinical depression: Results from a placebo-controlled, single-blinded randomized control trial. *J Affect Disord* 2018;**238**:342–50. doi:10.1016/j.jad.2018.06.025

114 Becker ES, Ferentzi H, Ferrari G, *et al.* Always Approach the Bright Side of Life: A General Positivity Training Reduces Stress Reactions in Vulnerable Individuals. *Cognit Ther Res* 2016;**40**:57–71. doi:10.1007/s10608-015-9716-2

For peer review only - http://bmjopen.bmj.com/site/about/guidelines.xhtml

115 Brockmeyer T, Schmidt U, Friederich H-C. The ABBA study - approach bias modification in bulimia nervosa and binge eating disorder: study protocol for a randomised controlled trial. *Trials* 2016;**17**:466. doi:10.1186/s13063-016-1596-6

116 Poreisz C, Boros K, Antal A, *et al.* Safety aspects of transcranial direct current stimulation concerning healthy subjects and patients. *Brain Research Bulletin* 2007;**72**:208–14. doi:10.1016/j.brainresbull.2007.01.004

117 Hutson PH, Balodis IM, Potenza MN. Binge-eating disorder: Clinical and therapeutic advances. *Pharmacol Ther* 2018;**182**:15–27. doi:10.1016/j.pharmthera.2017.08.002

118 Vroling MS, Wiersma FE, Lammers MW, *et al.* Predicting Dropout from Intensive Outpatient Cognitive Behavioural Therapy for Binge Eating Disorder Using Pre-treatment Characteristics: A Naturalistic Study. *Eur Eat Disord Rev* 2016;**24**:494–502. doi:10.1002/erv.2474

119 Marinilli Pinto A, Guarda AS, Heinberg LJ, *et al.* Development of the eating disorder recovery self-efficacy questionnaire. *Int J Eat Disord* 2006;**39**:376–84. doi:10.1002/eat.20256

120 Patton JH, Stanford MS, Barratt ES. Factor structure of the Barratt impulsiveness scale. *J Clin Psychol* 1995;**51**:768–74.

121 Bohn K, Fairburn CG. *The Clinical Impairment Assessment Questionnaire (CIA 3.0). In Cognitive Behavior Therapy and Eating Disorders.* New York: Guildford Press 2008.

122 Hoerger M, Quirk SW, Weed NC. Development and validation of the Delaying Gratification Inventory. *Psychol Assess* 2011;**23**:725–38. doi:10.1037/a0023286

For peer review only

For peer review only - http://bmjopen.bmj.com/site/about/guidelines.xhtml

Figure 1 Study procedure. The 3 assessment time points are baseline, post-assessment and follow-up. IG1 is intervention group 1; IG2 is intervention group 2, CG is the wait list control group.

Table 1 Study schedule of measurement and testing time points

**Approximate time since baseline**	**Screening of potential participants** -------	**Baseline assessment (all participants)** -------	**Training: 6 sessions of ABM + real tDCS 0-3 weeks**	**Training: 6 sessions of ABM + sham tDCS 0-3 weeks**	**Post-assessment (all participants 3 weeks**	**Follow-up (all participants) 7 weeks**
Informed consent		X				
EDDS, SCID-I	X					
TDCS safety screening	X					
Demographic information		X				
EDE-Q[83]		X			X	X
Inhibitory control tasks; Go/No-Go task[104], SST[106]		X			X	X
Delay Discounting Task with Money and Food[103]		X			X	X
Food related tasks; Food Choice Task[88], FCT, Bogus Taste Test[102]		X			X	
Approach bias assessment tasks; F-AAT[85], SRC[87]		X			X	X
Questionnaires & scales (incl. at home); EDRSQ[119], FCQ-T-r[94], PFS[95], YFAS 2.0[96], ERQ[107], PANAS[108], BIS-11[120], CIA[121], DGI[122], DASS-21[109]		X			X	X
Pre-[tDCS + ABM] measures: -Multiple VAS, blood pressure, pulse			X	X		
Real or sham tDCS to dLPFC			X	X		
Approach Bias Modification training			X	X		
Post-[tDCS + ABM] measures: -Multiple VAS, blood pressure, pulse			X	X		
Tolerance, discomfort and side effects			X	X		
Acceptability questionnaire						X
Blinding assessment questionnaire						X

BIS-11; Barrett Impulsiveness Scale, CIA; Clinical Impairment Assessment, DASS-21; Depression, Anxiety and Stress Scale, DGI; Delayed Gratification Inventory, EDDS; Eating Disorder Diagnostic Screen, EDE-Q; Eating Disorder Examination, EDRSQ; Eating Disorder Recovery Self Efficacy Questionnaire, ERQ; Emotion Regulation Questionnaire, FCQ-T-r; Food Cravings Questionnaire Trait Version – reduced, PANAS; Positive and Negative Affect Schedule, PFS; The 21-item Power of Food Scale, SCID-I; Structured Clinical Interview for Diagnostic and Statistical Manual (DSM) Axis I Disorders, SRC; Stimulus Response Compatibility Task, SST; Stop Signal Task, YFAS 2.0; The Yale Food Addiction Scale Version 2.0, .

For peer review only - http://bmjopen.bmj.com/site/about/guidelines.xhtml

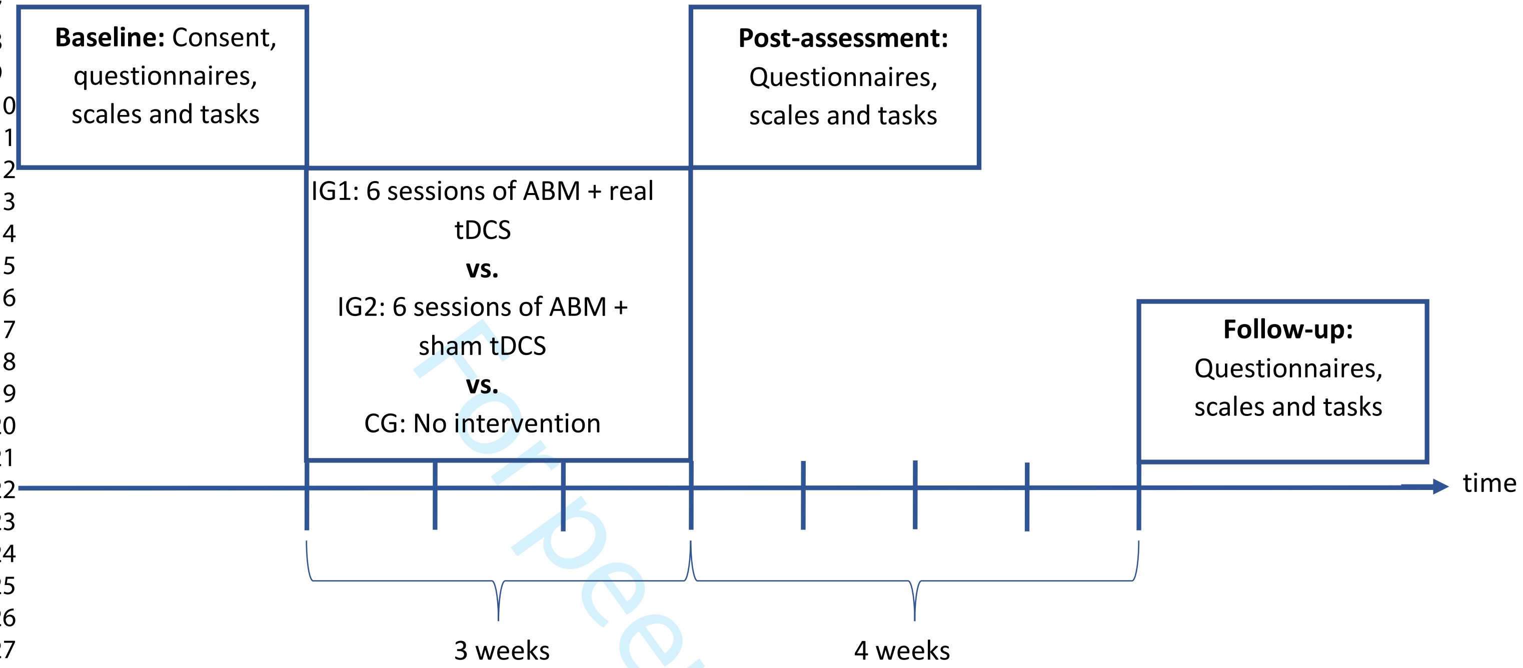

For peer review only

Supplementary File

Appendix A: Information sheet for all participants

PARTICIPANT INFORMATION SHEET

ICARUS: An Investigation of Approach Bias Modification Training (ABM) and Transcranial Direct Current Stimulation (tDCS) in Binge Eating Disorder

IRAS Project ID: 244170
Research Ethics Committee reference number: 18/NW/0648

We would like to invite you to participate in this postdoctoral research project which is being conducted by a PhD student for research and educational purposes. You should only participate if you want to; choosing not to take part will not disadvantage you in any way. Before you decide whether you want to take part, it is important for you to understand why the research is being done and what your participation will involve. Please take time to read the following information carefully and discuss it with others if you wish. Part 1 tells you the purpose of this study and what will happen to you if you take part. Part 2 gives you more detailed information about the conduct of the study. Please ask us if there is anything that is not clear or if you would like more information.

PART ONE

What is the purpose of the study?
Psychological therapy as a main treatment for Binge Eating Disorder (BED) may not be effective for many people and may not be readily accessible in some areas. Medical treatments for BED can have side effects and often do not remain effective in the long-term. Therefore, there is an ongoing need for the development of new treatments.

Computerised approach bias modification training (ABM) is a specific form of cognitive bias modification (CBM) that has been used to successfully treat mental disorders such as anxiety, depression, and addictive disorders. This technique involves several sessions of computerised training, a procedure which has shown to be effective in reducing the severity of some eating disorder symptoms in people with BED and Bulimia Nervosa (BN). ABM works as such; automatic approach and avoidance tendencies towards food-related cues are modified by repeated training of arm movements in front of a computer screen. ABM has shown to reduce approach tendencies and attention towards food cues in a subclinical sample of eating disorders involving binge eating, but its efficacy on these features in people with full-syndrome eating disorders remains unclear. Further research is needed to examine if ABM is effective in reducing the frequency of binge eating episodes in people with BED.

Transcranial direct current stimulation (tDCS) is a non-invasive technique that is capable of stimulating specific brain areas. Research shows that the frontal areas of the brain play a role in the development and maintenance of eating disorders, including BED. Stimulating these brain areas to alter their functioning is therefore believed to have the

For peer review only - http://bmjopen.bmj.com/site/about/guidelines.xhtml

potential to reduce eating disorder symptoms. This involves the delivery of a low electrical current via small electrodes placed on the scalp. This procedure is widely used in research and is being applied in clinical settings. Recent research using tDCS on people with BED has suggested that it may be helpful in reducing immediate food intake and cravings, and may decrease the frequency of a desire to binge eat at home after the treatment.

Combining ABM and tDCS

Previous studies suggest that these two techniques potentially help people better regulate their behaviours through similar mechanisms in the brain. Delivering both treatments together at the same time may have a stronger effect on reducing eating disorder symptoms in people with BED than either of the treatments alone. This will be the first time that this specific combination of interventions is conducted on people with an eating disorder.

In the present study, we aim to investigate combined ABM and tDCS as a treatment for BED by comparing the effect of 6 sessions of (ABM + real tDCS) vs. (ABM + placebo tDCS) across a 3-week period in adult men and women with BED. We will also compare these two groups against a control group. Participants will be allocated by chance to either one of two intervention groups, or to the control group. Participants in the intervention groups will receive 6 sessions of ABM delivered simultaneously with either real tDCS or a placebo version of tDCS. Participants assigned to the control group will not receive any intervention. We will measure eating disorder symptoms and other outcomes in all participants at baseline, post-treatment, and at the 4-week follow-up to assess outcomes of each study group. In particular, we are interested in changes in the frequency of binge eating and craving, and thought processes and emotions related to food and eating. We will also ask participants about their experience of this treatment. Participants assigned to the control condition will be offered 6 sessions of (ABM + real tDCS) after they have completed their involvement in the study.

Why have I been invited?

You are invited to participate if you are a male or female aged between 18 and 70 who has a current diagnosis of binge eating disorder (BED). We will be recruiting 66 participants in total.

Do I have to take part?

You do not have to take part in this experiment; it is your choice. If you decide to take part, you will be asked to sign three identical consent forms. You will be free to withdraw from the study at any time without giving a reason. Whether you decide to take part or not will in no way influence your care or the timing of your treatment.

What will happen to me if I take part and what will I have to do?

If you decide you want to participate you will firstly be asked to engage in a telephone conversation with the researcher (lasting approximately 20 minutes) to confirm that you are eligible to take part. If you are, you will be invited to the Institute of Psychiatry, Psychology and Neuroscience (Kings College London, Denmark Hill Campus) for a baseline assessment session on a day that is convenient for both you and the researcher, in either the morning or the afternoon. On this day, the researcher will discuss the study with you in person, answer your questions, and if you are happy to take part, we will

For peer review only - http://bmjopen.bmj.com/site/about/guidelines.xhtml

ask you to sign three copies of a consent form: one for you to keep, one for us to keep, and one that will be sent to your general practitioner (GP).

This baseline assessment visit is longer that the treatment visits and is comprised of an in-person visit and online questionnaires to complete at home. During the visit, you will give informed study consent, complete two questionnaires, neuropsychological tasks (brain puzzles), and a food task for which you will be asked to rate different foods. This visit also involves an assessment version of the approach bias modification (ABM) training programme, which is a computer based-task involving pushing and pulling a joystick in response to shapes appearing on the computer screen. Weight and height will be measured at each assessment, and participants may choose not to see the figures recorded. Assessment visits will last 65-75 minutes. Within 36 hours after the in-person visit, you will be emailed a link to a series of questionnaires (assessing mood and eating disorder-related thoughts and habits) which will take 30-45 minutes to complete. There is also the option to complete these assessment questionnaires in hardcopy paper format.

You will then be then randomised and informed of your randomly assigned study condition within a week. If you are assigned to one of the two intervention conditions, you will be asked to attend 6 sessions where you will perform the ABM task while receiving either real or placebo tDCS, in addition to completing the post-treatment and follow-up assessments. If you are assigned to the control group, you will not receive any intervention, and will be asked to attend the post-treatment and follow-up assessments.

All 6 intervention sessions will be identical and last approximately 40–50 minutes. There is no need for any special preparation before the visits. Before and after each intervention session, you will complete some scales related to aspects of your mood and level of hunger. Your blood pressure and pulse will also be measured before and after each intervention session to monitor your wellbeing.
During the (ABM + real/placebo tDCS) session you will sit on a comfortable chair facing a computer screen, with a joystick on a table in front of you. You will wear a plastic headband to keep the two tDCS electrodes in place (as shown in the diagram below). The electrodes will be placed in small sponges soaked in a salt water solution, so they might feel a bit wet against your head. The researcher will turn the machine on which will deliver the currents. Depending on your assigned study condition, you will receive real or placebo brain stimulation. The placebo session will be the same as the real session, but the tDCS machine won’t deliver any electrical current. Most people can’t tell the difference between real and placebo tDCS sessions.

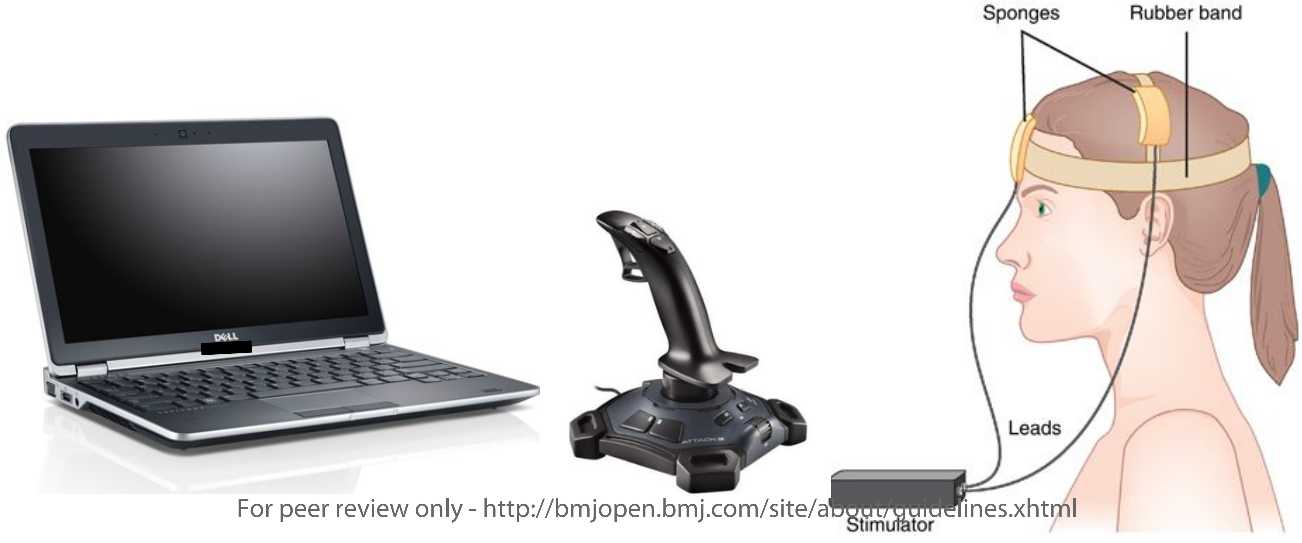

The tDCS will begin a few minutes prior to the start of the computer training programme, to allow participants to become used to the sensation before starting the ABM task on the computer. The training version of the ABM programme is a computer based-task involving pushing and pulling a joystick in response to shapes appearing on the computer screen. The tDCS will also continue for a few minutes after the ABM task has ended. You will then be asked to rate any discomfort experienced during the session due the tDCS.

You will be asked to return to the Institute for 5 more identical intervention sessions within 3 weeks, leaving a gap of at least 24 hours between each session. The post-assessment will be conducted immediately after the 6th intervention session. This in-person visit comprising of the final intervention session plus the post-assessment will therefore last 90-120 minutes, with the online questionnaires to be completed within 36 hours after this visit (45 minutes). The follow-up assessment 4 weeks later will be similar to the baseline assessment. This final visit will therefore take 75 minutes, with the online questionnaires taking 45 minutes to complete at home afterwards.

20 participants (10 participants from each intervention condition) will be invited to provide feedback on their experiences of study participation, initial expectations of the intervention, perceived strengths and weaknesses, and suggestions for improvements in an interview with the researcher. Declining this invitation to interview does not affect study payment.

6 months after the baseline assessment, participants may be contacted by phone by a member of the King's College London Eating Disorders Unit for a brief phone call to evaluate the presence of eating disorder symptoms. This 6 month check-in will allow any long-term therapeutic effects of the study treatment intervention to be evaluated. Participation in this phone call will not affect participant payment, which will have been administered earlier after the 1-month follow up assessment.

Expenses and payments

Upon completion of the study, all participants will be paid a maximum of £60 for completing each of the three assessment sessions (comprised of an in-person visit and at-home questionnaires); the baseline assessment, post-treatment assessment and follow-up assessment (£20 each). This payment should be declared for tax and/or benefit purposes. If you are assigned to an intervention condition involving 6 sessions of brain stimulation and computer training, you may also be compensated up to £10 per day for your travel expenses on these intervention session days.

What is expected from you as a participant?

We would expect you to complete all assessment sessions (pre-, post-treatment, follow-up), and if you are randomised to an intervention group, to attend all 6 (ABM + real/placebo tDCS) sessions as scheduled. We ask you to inform us immediately if for any reason you suddenly find yourself unable to attend a scheduled session.

For peer review only - http://bmjopen.bmj.com/site/about/guidelines.xhtml

Please let us know of any health problem that has developed, or any new diagnosis made since you enrolled for the study. Further, we would ask you to let us know of any new medication or change in medication whilst you are taking part in the study.

What are the possible disadvantages and risks of taking part? What are the side effects?
Combined brain stimulation and cognitive training sessions are time-consuming and may cause fatigue from concentrating on the task.

TDCS has been shown to be safe when used correctly in a clinical setting. However, you may find the procedure slightly uncomfortable. This is because a number of sensations can occur beneath the electrodes during stimulation including tingling, pain, itching, and burning. Not everyone feels these sensations or finds them uncomfortable, but if you do, remember you are free to stop the study at any point without giving an explanation. In some rarer cases, tDCS has been known to cause a headache, but this can be treated with mild painkillers (e.g. paracetamol). No side effects of ABM are known. We will assess any discomfort you may experience during intervention sessions throughout your involvement in the trial.

What are the possible benefits of taking part?
Unfortunately, there are no direct benefits to taking part in this study, but the information we get may help us to improve the treatment of BED in the future.

What happens when the research study stops?
When the research study stops, no further ABM + tDCS sessions will be available to those who have received 6 sessions. Participants in the control group will have the option of receiving 6 sessions of [ABM + real tDCS] once they have completed the waiting period and follow-up assessment.
Participants assigned to one of the intervention groups can request to be informed if they had received ABM combined with real or placebo tDCS, once they have completed the follow-up assessment.

What if there is a problem?
Any complaint about the way you have been dealt with during the study or any possible harm you might suffer will be addressed. Detailed information on this is given in Part 2.

Will my taking part in the study be kept confidential?
Yes. We will follow ethical and legal practice and all information about you will be handled in confidence. The details are included in Part 2.

If the information in Part 1 has interested you and you are considering participation, please read the additional information in Part 2 before making a decision.

PART TWO

What if relevant and new information becomes available?
Sometimes we get new information about the treatment being studied. This is not expected to occur given the short time frame of participation (6 sessions across 3

For peer review only - http://bmjopen.bmj.com/site/about/guidelines.xhtml

weeks); however, if any new and relevant information becomes available during this time we will inform you immediately. You can then decide whether you wish to continue in the study.

What will happen if I don't want to carry on with the study?
Your participation is voluntary and you do not have to take part in the study. You can change your mind at any point and terminate your participation without giving a reason to the researcher. You are free to withdraw from this study at any time without consequence.

Will participation in this study affect my routine healthcare, or the waiting period for treatment for my eating disorder if I am currently on a waiting list?
No. Participation in this study will have no impact on your treatment as usual, or waiting time if you are currently awaiting treatment for your eating disorder. We fully encourage you to begin treatment as provided by a health care professional as soon as it becomes available to you. We simply ask that you inform us of any changes to your treatment or medication while you are partaking in the study.

What if there is a problem?
If you have a concern about any aspect of the study, please ask the researcher (gemma.gordon@kcl.ac.uk, 0207 848 0183) who will do their best to answer your questions.

What if I wish to make a complaint?
If you remain unhappy and wish to formally complain, complaints to the IoPPN should be addressed to Dr Gill Dale. Director of Research Quality; Head, Joint R&D Office of South London and Maudsley NHS Foundation Trust and Institute of Psychiatry, Psychology & Neuroscience (IoPPN), P005, Institute of Psychiatry, Psychology & Neuroscience (IoPPN), King's College London, De Crespigny Park, London SE5 8AF. NHS complaints will follow NHS complaints procedures.

Other sources of support for your eating disorder
To access support and treatment, please see your Eating Disorders clinician or your GP who will be able to advise you and refer you to the right service for you. You can also obtain further information and support from www.beateatingdisorders.org.uk, the national Eating Disorders charity.

Should you wish to speak to someone outside of the university, please talk to your Eating Disorders clinician, GP, the Beat helpline, and/or one of the study researchers who is happy to liaise on your behalf if you so wish. The eating disorders charity Beat provides helplines for adults and young people which offer support and information to sufferers, carers and professionals. Further information can be found on their website www.beateatingdisorders.org.uk, or by ringing their helpline 0808 801 0677.

Will my taking part in the study be kept confidential?
Your personal information and the data we collect from you will remain confidential at all times. It will also remain anonymous to everyone apart from the primary researchers. Manual files will be locked securely in a filing cabinet, which will be kept in a locked office in the KCL Section of Eating Disorders, Department of Psychological

For peer review only - http://bmjopen.bmj.com/site/about/guidelines.xhtml

Medicine, IoPPN, and all electronic files will be password protected. All information which is collected during the course of the research will be kept strictly confidential according to the General Data Protection Regulation (GDPR), brought into effect on 25th May, 2018. This new legislation creates some new rights for individuals to better reflect data protection challenges in the modern digital age, as well as strengthening some of the rights that currently exist under the Data Protection Act 1998.

How will my personal data be used and what are my rights?
King's College London is the sponsor for this study based in the United Kingdom. We will be using information from you in order to undertake this study and will act as the data controller for this study. This means that we are responsible for looking after your information and using it properly. King's College London will keep identifiable information about you for four years after the study has finished.
Your rights to access, change or move your information are limited, as we need to manage your information in specific ways in order for the research to be reliable and accurate. If you withdraw from the study, we will keep the information about you that we have already obtained. To safeguard your rights, we will use the minimum personally-identifiable information possible. You can find out more about how we use your information by contacting the Study Coordinator Gemma Gordon.

KCL will use your name and contact details to contact you about the research study, and make sure that relevant information about the study is recorded for your care, and to oversee the quality of the study. Individuals from KCL and regulatory organisations may look at your medical and research records to check the accuracy of the research study. SLaM will pass these details to KCL along with the information collected from you. The only people in KCL who will have access to information that identifies you will be people who need to contact you regarding your participation or audit the data collection process. The people who analyse the information will not be able to identify you and will not be able to find out your name or contact details. KCL will keep identifiable information about you from this study for four years after the study has finished.

Involvement of the General Practitioner (GP)
As a matter of courtesy and in the interest of your wellbeing, we may let your GP know about your participation in the study, and may request your permission to send them a letter when you enroll. If you agree to this, you will be asked to provide us with your GP's contact details so that we can send them a letter with details of the research.

Insurance/indemnity
Standard KCL insurance and NHS indemnity arrangements apply.

Involvement of the insurance company
If you have private medical insurance, you should inform your insurance company that you are taking part in this study.

Will any genetic tests be done?
No.

What will happen to the results of the research study?

For peer review only - http://bmjopen.bmj.com/site/about/guidelines.xhtml

You will be offered the opportunity to be informed about your individual results once the data for all participants has been collected. If you want written feedback of the study's findings you can contact the researcher (gemma.gordon@kcl.ac.uk) for a lay summary. The results will be included in an examined postgraduate report, presented as part of a postgraduate presentation, and sent to a medical journal for publication. Your participation in the study will not be disclosed.

Who is organising and funding the research?

This study is being funded by King's College London.

Who has reviewed the study?

All research in the NHS is looked at by an independent group of people, called a Research Ethics Committee, to protect your interests. This study has been reviewed and given favourable opinion by North West -Liverpool East Research Ethics Committee.

Further information and contact details

If you have any questions or require more information about this study, please contact the researcher using the following contact details:

Gemma Gordon (gemma.gordon@kcl.ac.uk) (0207 848 5608)
Section of Eating Disorders, Department of Psychological Medicine
KCL Institute of Psychiatry, Psychology and Neuroscience,
16 De Crespigny Park
London, SE5 8AF

Appendix B: Consent form for all participants

CONSENT FORM

IRAS Project ID: 244170
Research Ethics Committee reference number: 18/NW/0648

Please complete this form after you have read the information sheet and listened to an explanation about the research.

Title of Study: ICARUS - An Investigation of Approach Bias Modification Training (ABM) and Transcranial Direct Current Stimulation (tDCS) in Binge Eating Disorder

Name of Researcher: Gemma Gordon

Thank you for considering taking part in this research. The person organising the research must explain the project to you before you agree to take part. If you have any questions arising from the information sheet or explanation already given to you, please ask the researcher before you decide whether to join in. You will be given a copy of this consent form to keep and refer to at any time.

Please initial box

For peer review only - http://bmjopen.bmj.com/site/about/guidelines.xhtml

1. I confirm that I have read the information sheet dated 18.09.2018 (version 3) for the above study. I have had the opportunity to consider the information, ask questions and have had these answered satisfactorily. ☐

2. I understand that my participation is voluntary and that I am free to withdraw at any time without giving a reason, without my medical care or legal rights being affected. ☐

3. I consent to the processing of my personal information for the purposes explained to me. I understand that such information will be handled in accordance with the terms of the General Data Protection Regulation (GDPR) 2018. I understand that confidentiality and anonymity will be maintained and it will not be possible to identify me in any publications. ☐

4. I know that if I would like to, I can contact the research team and request a written summary of the study findings. ☐

5. I understand that I must not take part if I have any of the conditions listed in the exclusion criteria. ☐

6. I understand that during study participation, I must inform the researcher of any changes in my medication or of any new medical diagnoses made. ☐

7. I agree to my General Practitioner (GP) being informed of my participation in this study. ☐

8. **I consent/do not consent** to be contacted in the future by King's College London researchers who would like to invite me to participate in follow up studies to this project, or in future studies of a similar nature. ☐

9. I agree to take part in the above study. ☐

Participant's Statement:

I ____

agree that the research project named above has been explained to me to my satisfaction and I agree to take part in the study. I have read both the notes written above and the Information Sheet about the project, and understand what the research study involves.

Signed **Date**

Investigator's Statement:

For peer review only - http://bmjopen.bmj.com/site/about/guidelines.xhtml

I ____
Confirm that I have carefully explained the nature, demands and any foreseeable risks (where applicable) of the proposed research to the participant.

Signed **Date**

<u>For administration purposes, please indicate your preference below</u>

Q: In what format would you like to complete the at-home assessment questionnaires?

Tick '√' in one box to indicate your choice

1. I would like to complete the assessment questionnaires online ☐

2. I would like to receive a hardcopy paper version of the questionnaires in a stamped addressed envelope, to complete and post back to the researcher within 36 hours of the in-person assessment visit. ☐

Enquiries:
Gemma Gordon (gemma.gordon@kcl.ac.uk)
Department of Psychological Medicine
Section of Eating Disorders
KCL Institute of Psychology, Psychiatry and Neuroscience,
16 De Crespigny Park
London, SE5 8AF
Phone: 0207 848 5608

Appendix C: ICARUS study task, questionnaire, measure and scale information

(1) *Approach bias assessment tasks*

(i) Food Approach-Avoidance Task (F-AAT) (Rinck & Becker, 2007): The F-AAT is a computerised task that measures approach and avoidance behaviour by means of joystick movements in response to food and neutral stimuli presented on a computer screen. This task will be used to assess approach bias towards visual cues of high-calorie food (expected target cognitive mechanism of intervention). Images of palatable edible foods (i.e. chocolate, pizza) and non-edible objects (i.e. sponges, stapler) are used as in previously (Brockmeyer et al., 2019). The assessment version of the AAT is identical to the treatment version except that the required response is unrelated to the picture content (i.e. food and neutral stimuli are presented equally often in round and rectangular, i.e. push and pull, format). Format movement assignments are counterbalanced among participants (i.e., half push round pictures and half push rectangular pictures). When the joystick is pulled, the picture grows bigger, and diminishes in size when the joystick is pushed. Zooming-in and

For peer review only

zooming-out via joystick movements enacts motions of approaching and avoiding respectively and thus combines the proprioceptive (arm movement) and exteroceptive (zooming feature) cues of approach and avoidance behaviour (Neumann & Strack, 2000; Brockmeyer et al., 2019). The assessment version of the AAT consists of 80 trials (40 food item pictures and 40 non-food item pictures). To evaluate approach bias towards food, a compatibility score is calculated by subtracting the median reaction times (RTs) of compatible trials (i.e., RT pull food + RT push nonfood) from median RTs of incompatible trials (i.e., RT push food + RT pull nonfood) (Brockmeyer et al., 2019; Becker et al., 2016; Vrijsen et al., 2018). A positive value indicates a food-specific approach bias (i.e. the participant is faster at pulling than pushing food pictures, relative to the approach bias towards non-food), whereas a negative value indicates an avoidance bias. This task will be performed using Inquisit 5 (Millisecond Software).

(ii) The Stimulus Response Compatibility Task (SRC) (De Houwer, Crombez, Baeyens, & Hermans, 2001): In this task, participants respond to images on a computer screen by pressing keys on the keyboard. Pictures are presented in the centre of the screen with a manikin (12 mm high) positioned 33 mm above or below the picture. Participants are required to categorise the presented pictures by making an approach response (pressing the *up* or *down* key to move the manikin toward the picture) or an avoidance response (pressing the *up* or *down* key to move the manikin away from the picture). After making a correct response, an animation is shown of the manikin walking toward the picture (approach) or away from the picture (avoidance) for 1,000 ms. After making an incorrect response, a red cross appears on the screen for 500 ms, after which the next trial starts. Fourteen food and fourteen non-food pictures will be used from the food-pics database (Blechert, Meule, Busch & Ohla, 2014). The experiment comprises 8 practice trials and 56 experimental trials. Bias scores will be calculated by subtracting the mean of approach food/avoid non-food trials from the mean of avoid food/approach non-food trials. A positive score indicates a food-related approach bias, with higher scores indicative of stronger biases. This task will be performed using Inquisit 5 (Millisecond Software).

(2) Food Choice Task

The Food Choice Task (Hare, Camerer & Rangel., 2009) adapted for eating disorders (Steinglass et al., 2015) is a computer-based paradigm that measures responses to images of foods to assess food attitudes and characteristics of eating behaviour. Food stimuli is used to investigate how individuals make decisions about what to eat, and measures decision-making around food by directly probing personal preferences. There are no learning requirements and individualised assessments of food along two dimensions (healthiness and tastiness) allow the tasks to be used in diverse populations with differing valuations of food. The task consists of three phases. In each phase participants are presented with 43 images of food items. The food items represent an array of dietary options (Steinglass et al., 2015). Twenty-five food items are low fat (<30% calories from fat) and 18 are high fat (>30% calories from fat), as determined by Foerde et al. (2018). In the Health phase, participants rate the healthiness of each food item on a 5-point scale (*1 = "Unhealthy", 5 = "Healthy"*). In the Taste phase, participants rate the tastiness of each food item in a similar fashion, (*1 = "Bad", 5 = "Good"*). In the Choice phase, in each trial participants choose between the presented food item and a "Neutral" reference food item (rated as 3 in both Health and Taste phases). If no item is rated 3 on both scales, an item rated 3 on Health and greater

For peer review only - http://bmjopen.bmj.com/site/about/guidelines.xhtml

than 3 on the Taste scale is selected as a reference food. Good test-retest reliability of the FCT suggests that it is suitable for measuring food-based decision-making in studies with multiple assessment points (Foerde et al., 2018).

(3) *State food craving - Food craving after cue exposure task*
The Food Challenge Task (FCT) (Kekic et al., 2017; Meule et al., 2018) will be used to examine cue-induced food craving. In this task, participants rate their state food craving using the Food Cravings Questionnaire State Version (Cepeda-Benito et al., 2000; Meule et al., 2012) before and after being presented with a video on a computer screen of foods shown to be highly appetitising and to elevate hunger levels (Kekic et al., 2017). The questionnaire consists of 15 items that measure the strength of food cravings (i.e. *I would feel more alert if I could satisfy my craving*). Participants are asked to indicate how much they agree with each statement 'at this very moment' using a five-point scale (from 1 'strongly disagree' to 5 'strongly agree'). There are five craving subscales; intense desire to eat, anticipation of relief from negative states, physiological craving, preoccupation with food or lack of control over eating and anticipation of positive reinforcement. Scores can be calculated for specific subscales or a total score can be calculated (ranging from 15 to 75). This questionnaires will be completed using Inquisit 5 (Millisecond Software), and the video shown using QuickTime player.

(4) *Trait food craving*
The Yale Food Addiction Scale Version 2.0 (YFAS 2.0; Gearhardt, Corbin, & Brownell, 2016) is the most commonly used instrument to assess food-related addictive behaviours (Steward et al., 2018). This self-report questionnaire consists of 35 items scored on an 8-point Likert scale (from 0 = never to 7 = every day) and is adapted to assess addictive eating behaviours based on DSM-5 substance-related and addictive disorders criteria (APA, 2013). It refers specifically to consumption of foods high in fat, sugar, salt or refined carbohydrates. It includes items that assess specific criteria, such as diminished control over consumption, a persistent desire or repeated unsuccessful attempts to quit, withdrawal, and clinically significant impairment (i.e. *'I kept eating in the same way even though my eating caused emotional problems')*. The YFAS includes two scoring options: 1) a "symptom count" ranging from 0 to 7 that reflects the number of addiction-like criteria endorsed and 2) a categorical scoring option that classifies respondents as having either no, mild, moderate or severe 'food addiction'. The YFAS has received psychometric support in binge eating populations (Gearhardt, White et al., 2013; Carter, Van Wijk & Rowsell, 2019), and obese bariatric surgery patients (Clark & Saules, 2013; Meule, Heckel, & Kübler, 2012). The YFAS 2.0 was developed to maintain consistency with the current diagnostic understanding of addiction and to improve the psychometric properties of the original YFAS. Exceeding the food addiction threshold was more strongly associated with obesity for the YFAS 2.0 than the original YFAS. The YFAS 2.0 has demonstrated good internal consistency (Carter, Van Wijk & Rowsell, 2019), as well as convergent, discriminant and incremental validity (Gearhardt, Corbin & Brownell, 2016).

(5) *Food intake in a Bogus Taste Test*
Actual food consumption will be measured by means of a Bogus Taste Test (Robinson et al.,

For peer review only - http://bmjopen.bmj.com/site/about/guidelines.xhtml

2017); a food consumption test presented under the guise of a taste test. Participants will be instructed to rate 3 bowls of highly palatable high-calorie food items (chocolate, sweets, crisps) in terms of their visual attractiveness, smell, and taste on a paper form. The researcher will inform the participant that she/he will leave the room for 10 minutes and during this time they can complete their ratings and are free to eat as much of the offered items as they like. A small bin with lid will be provided and participants will be instructed to consume as much food as they need or want, and to discard the remainder of the food items in the bin before 10 minutes are over. After the participant has left, the discarded food items will be recovered from the bin, sorted, and placed in their original bowls. Consumption will be determined by weighing the bowls both before and after the “taste test” and the difference in weight from pre-to post-assessment will be converted into calories and used as a measure of food intake.

(6) *Delay discounting*

Delay Discounting Task with Money and Food (Odum, Baumann, & Rimington, 2006): Participants indicate their preferences in a series of choices for two hypothetical outcome types: immediate versus delayed food and immediate versus delayed money. Participants make choices involving either relatively small maximum amounts of food (10 dollars worth) and money (10 dollars) or for relatively large maximum amounts of food (100 dollars worth) and money (100 dollars). Performance on this task can be used to study self-regulation, delayed gratification and valuation of reward. This task will be performed using Inquisit 5 (Millisecond Software).

(7) *Inhibitory control*

(i) Go/No-Go task: The cued go/no go task is a useful measure of impulse control in clinical populations. This task is a classic test of executive function, requiring effortful response inhibition. The food specific version of the cued go no-go task (Teslovich et al., 2014) measures impulsivity and response inhibition with respect to appetising food and nonfood items (i.e. toys), via assessing the ability to inhibit instigated, prepotent responses. The task manipulates response prepotency by presenting a preliminary go or no-go cue before the actual go or no-go target is displayed. The cues provide information concerning the probability that a go or no-go target will be presented. The cue-target relationship is manipulated so that the cues have a high probability of correctly signaling a go or no-go target (valid cues), and a low probability of incorrectly signaling a target (invalid cues). Valid cues tend to facilitate response inhibition and speed response execution, whereas invalid cue cues tend to impair response inhibition and slow response execution. The set of stimuli consists of 30 colour images of common high- (8) and low-calorie (7) foods and common toys (15). The outcome variables include: (1) overall reaction time (RT) in milliseconds during correct “go” trials, (2) rate of omission errors (missed “go” trials), and (3) false alarm rate (rate at which participants erroneously press to a no-go stimulus). This task will be performed using Inquisit 5 (Millisecond Software).

(i) Stop Signal Task (SST): This is a task measuring inhibitory control. Participants are required to engage in a computer task but withhold their response in the presence of a stop signal. This SST includes food-specific and neutral non-food stimuli in the same task (adaptation of Manasse et al., 2016). This allows for isolation of any unique food specific inhibitory control deficits from general difficulties inhibiting responses. The outcome measure is the stop signal reaction time (SSRT). The SSRT is calculated for each set of stimuli

For peer review only - http://bmjopen.bmj.com/site/about/guidelines.xhtml

(i.e., SSRT stimulus type) for each subject by subtracting the average stop signal delay from the average reaction time on “go” trials (Verbruggen & Logan, 2008). The recording accuracy of reaction time and stop signal delay measurement is in milliseconds. A smaller SSRT is indicative of greater inhibitory control and a larger SSRT reflects weaker/impaired inhibitory control. This task will be performed using Inquisit 5 (Millisecond Software).

(8) *Mood and emotion regulation*
The Emotion Regulation Questionnaire (ERQ) (Gross & John, 2003): A 10-item scale designed to measure respondents' tendency to regulate their emotions in two ways: (1) cognitive reappraisal and (2) expressive suppression. Respondents answer each item on a 7-point Likert-type scale ranging from 1 (strongly disagree) to 7 (strongly agree). This will be administered at pre-assessment, post-assessment and follow-up.

Positive and Negative Affect Schedule (PANAS) (Watson, Clarke & Tellegen, 1988): The PANAS consists of two 10-item self-report scales which measure positive and negative affect. On a Likert scale ranging from 0 (very slightly or not at all) to 4 (extremely), participants rate the extent to which they have experienced each of the 20 descriptors within a particular time frame (“right now” in the current study). Two scores are generated: positive (PANAS-positive) and negative (PANAS- negative) affect. This will be administered at pre-assessment, post-assessment and follow-up.

Depression, Anxiety and Stress Scale (21-item version; DASS-21) (Lovibond & Lovibond, 1995): This is a 21 item self-report questionnaire which aims to evaluate mood, anxiety and stress levels over the previous week. The DASS-21 will be administered at pre-assessment, post-assessment and follow-up.

(9) *Within session measures (immediately after each [ABM+real/sham tDCS] treatment session)*
(i) Paper-based Visual Analogue Scales (VAS) assessing current hunger, feeling of fullness, urge to eat, urge to binge eat, feeling low, level of tension, level of stress, level of anxiety. These scales consist of a 10cm line. Participants are requested to indicate on this line a degree or level of a specific emotion or behavioural urge. There are indications of what range (e.g. from 'not at all' to 'extremely').

(ii) Tolerance, discomfort and side effects: An evaluation of discomfort with the training session (tDCS and ABM aspects separately) will be completed with another paper-based 10cm VAS (rated from none to extreme discomfort). Participants will be asked to report any side effects in an open ended question.

For peer review only - http://bmjopen.bmj.com/site/about/guidelines.xhtml

SPIRIT

STANDARD PROTOCOL ITEMS: RECOMMENDATIONS FOR INTERVENTIONAL TRIALS

SPIRIT 2013 Checklist: Recommended items to address in a clinical trial protocol and related documents*

Section/item	Item No	Description	Addressed on page number
**Administrative information**			
Title	1	Descriptive title identifying the study design, population, interventions, and, if applicable, trial acronym	________1______
Trial registration	2a	Trial identifier and registry name. If not yet registered, name of intended registry	________1______
	2b	All items from the World Health Organization Trial Registration Data Set	______________
Protocol version	3	Date and version identifier	______1_______
Funding	4	Sources and types of financial, material, and other support	______12_______
Roles and responsibilities	5a	Names, affiliations, and roles of protocol contributors	______12______
	5b	Name and contact information for the trial sponsor	______12_______
	5c	Role of study sponsor and funders, if any, in study design; collection, management, analysis, and interpretation of data; writing of the report; and the decision to submit the report for publication, including whether they will have ultimate authority over any of these activities	______12______
	5d	Composition, roles, and responsibilities of the coordinating centre, steering committee, endpoint adjudication committee, data management team, and other individuals or groups overseeing the trial, if applicable (see Item 21a for data monitoring committee)	_____11____

For peer review only

**Introduction**			
Background and rationale	6a	Description of research question and justification for undertaking the trial, including summary of relevant studies (published and unpublished) examining benefits and harms for each intervention	_____2-4___
	6b	Explanation for choice of comparators	______4__
Objectives	7	Specific objectives or hypotheses	______4___
Trial design	8	Description of trial design including type of trial (eg, parallel group, crossover, factorial, single group), allocation ratio, and framework (eg, superiority, equivalence, noninferiority, exploratory)	______5______
**Methods: Participants, interventions, and outcomes**			
Study setting	9	Description of study settings (eg, community clinic, academic hospital) and list of countries where data will be collected. Reference to where list of study sites can be obtained	_____6_____
Eligibility criteria	10	Inclusion and exclusion criteria for participants. If applicable, eligibility criteria for study centres and individuals who will perform the interventions (eg, surgeons, psychotherapists)	______5_____
Interventions	11a	Interventions for each group with sufficient detail to allow replication, including how and when they will be administered	____9-10___
	11b	Criteria for discontinuing or modifying allocated interventions for a given trial participant (eg, drug dose change in response to harms, participant request, or improving/worsening disease)	____11___
	11c	Strategies to improve adherence to intervention protocols, and any procedures for monitoring adherence (eg, drug tablet return, laboratory tests)	____11____
	11d	Relevant concomitant care and interventions that are permitted or prohibited during the trial	____4, 11___
Outcomes	12	Primary, secondary, and other outcomes, including the specific measurement variable (eg, systolic blood pressure), analysis metric (eg, change from baseline, final value, time to event), method of aggregation (eg, median, proportion), and time point for each outcome. Explanation of the clinical relevance of chosen efficacy and harm outcomes is strongly recommended	____6-9___

For peer review only - http://bmjopen.bmj.com/site/about/guidelines.xhtml

Participant timeline	13	Time schedule of enrolment, interventions (including any run-ins and washouts), assessments, and visits for participants. A schematic diagram is highly recommended (see Figure)	___6__
Sample size	14	Estimated number of participants needed to achieve study objectives and how it was determined, including clinical and statistical assumptions supporting any sample size calculations	____5___
Recruitment	15	Strategies for achieving adequate participant enrolment to reach target sample size	___11__

Methods: Assignment of interventions (for controlled trials)

Allocation:

Sequence generation	16a	Method of generating the allocation sequence (eg, computer-generated random numbers), and list of any factors for stratification. To reduce predictability of a random sequence, details of any planned restriction (eg, blocking) should be provided in a separate document that is unavailable to those who enrol participants or assign interventions	____5-6___
Allocation concealment mechanism	16b	Mechanism of implementing the allocation sequence (eg, central telephone; sequentially numbered, opaque, sealed envelopes), describing any steps to conceal the sequence until interventions are assigned	____6___
Implementation	16c	Who will generate the allocation sequence, who will enrol participants, and who will assign participants to interventions	____5____
Blinding (masking)	17a	Who will be blinded after assignment to interventions (eg, trial participants, care providers, outcome assessors, data analysts), and how	___5____
	17b	If blinded, circumstances under which unblinding is permissible, and procedure for revealing a participant’s allocated intervention during the trial	___6____

Methods: Data collection, management, and analysis

Data collection methods	18a	Plans for assessment and collection of outcome, baseline, and other trial data, including any related processes to promote data quality (eg, duplicate measurements, training of assessors) and a description of study instruments (eg, questionnaires, laboratory tests) along with their reliability and validity, if known. Reference to where data collection forms can be found, if not in the protocol	____6-8____

	18b	Plans to promote participant retention and complete follow-up, including list of any outcome data to be collected for participants who discontinue or deviate from intervention protocols	_____10-11____
Data management	19	Plans for data entry, coding, security, and storage, including any related processes to promote data quality (eg, double data entry; range checks for data values). Reference to where details of data management procedures can be found, if not in the protocol	_____10-11_____
Statistical methods	20a	Statistical methods for analysing primary and secondary outcomes. Reference to where other details of the statistical analysis plan can be found, if not in the protocol	_____10_____
	20b	Methods for any additional analyses (eg, subgroup and adjusted analyses)	_____10______
	20c	Definition of analysis population relating to protocol non-adherence (eg, as randomised analysis), and any statistical methods to handle missing data (eg, multiple imputation)	______10______
**Methods: Monitoring**			
Data monitoring	21a	Composition of data monitoring committee (DMC); summary of its role and reporting structure; statement of whether it is independent from the sponsor and competing interests; and reference to where further details about its charter can be found, if not in the protocol. Alternatively, an explanation of why a DMC is not needed	_____10_____
	21b	Description of any interim analyses and stopping guidelines, including who will have access to these interim results and make the final decision to terminate the trial	_____11______
Harms	22	Plans for collecting, assessing, reporting, and managing solicited and spontaneously reported adverse events and other unintended effects of trial interventions or trial conduct	_____11_____
Auditing	23	Frequency and procedures for auditing trial conduct, if any, and whether the process will be independent from investigators and the sponsor	_______n/a____
**Ethics and dissemination**			
Research ethics approval	24	Plans for seeking research ethics committee/institutional review board (REC/IRB) approval	______1, 13____

Protocol amendments	25	Plans for communicating important protocol modifications (eg, changes to eligibility criteria, outcomes, analyses) to relevant parties (eg, investigators, REC/IRBs, trial participants, trial registries, journals, regulators)	_____1, 12_____
Consent or assent	26a	Who will obtain informed consent or assent from potential trial participants or authorised surrogates, and how (see Item 32)	_____12_____
	26b	Additional consent provisions for collection and use of participant data and biological specimens in ancillary studies, if applicable	_____n/a_____
Confidentiality	27	How personal information about potential and enrolled participants will be collected, shared, and maintained in order to protect confidentiality before, during, and after the trial	____10-11____
Declaration of interests	28	Financial and other competing interests for principal investigators for the overall trial and each study site	_____12______
Access to data	29	Statement of who will have access to the final trial dataset, and disclosure of contractual agreements that limit such access for investigators	_____ 10_____
Ancillary and post-trial care	30	Provisions, if any, for ancillary and post-trial care, and for compensation to those who suffer harm from trial participation	______11_____
Dissemination policy	31a	Plans for investigators and sponsor to communicate trial results to participants, healthcare professionals, the public, and other relevant groups (eg, via publication, reporting in results databases, or other data sharing arrangements), including any publication restrictions	______1, 11_____
	31b	Authorship eligibility guidelines and any intended use of professional writers	______12______
	31c	Plans, if any, for granting public access to the full protocol, participant-level dataset, and statistical code	______n/a______
**Appendices**			
Informed consent materials	32	Model consent form and other related documentation given to participants and authorised surrogates	______24______
Biological specimens	33	Plans for collection, laboratory evaluation, and storage of biological specimens for genetic or molecular analysis in the current trial and for future use in ancillary studies, if applicable	______n/a______

For peer review only - http://bmjopen.bmj.com/site/about/guidelines.xhtml

*It is strongly recommended that this checklist be read in conjunction with the SPIRIT 2013 Explanation & Elaboration for important clarification on the items. Amendments to the protocol should be tracked and dated. The SPIRIT checklist is copyrighted by the SPIRIT Group under the Creative Commons "Attribution-NonCommercial-NoDerivs 3.0 Unported" license.

For peer review only

CONSORT 2010 checklist of information to include when reporting a randomised trial*

Section/Topic	Item No	Checklist item	Reported on page No
**Title and abstract**			
	1a	Identification as a randomised trial in the title	1
	1b	Structured summary of trial design, methods, results, and conclusions (for specific guidance see CONSORT for abstracts)	1(no results)
**Introduction**			
Background and objectives	2a	Scientific background and explanation of rationale	2-4
	2b	Specific objectives or hypotheses	4-5
**Methods**			
Trial design	3a	Description of trial design (such as parallel, factorial) including allocation ratio	5
	3b	Important changes to methods after trial commencement (such as eligibility criteria), with reasons	n/a
Participants	4a	Eligibility criteria for participants	5
	4b	Settings and locations where the data were collected	6
Interventions	5	The interventions for each group with sufficient details to allow replication, including how and when they were actually administered	9-10
Outcomes	6a	Completely defined pre-specified primary and secondary outcome measures, including how and when they were assessed	6-9
	6b	Any changes to trial outcomes after the trial commenced, with reasons	n/a
Sample size	7a	How sample size was determined	5
	7b	When applicable, explanation of any interim analyses and stopping guidelines	11
Randomisation:			
Sequence generation	8a	Method used to generate the random allocation sequence	5-6
	8b	Type of randomisation; details of any restriction (such as blocking and block size)	5-6
Allocation concealment mechanism	9	Mechanism used to implement the random allocation sequence (such as sequentially numbered containers), describing any steps taken to conceal the sequence until interventions were assigned	6
Implementation	10	Who generated the random allocation sequence, who enrolled participants, and who assigned participants to interventions	5
Blinding	11a	If done, who was blinded after assignment to interventions (for example, participants, care providers, those	5

For peer review only

For peer review only - http://bmjopen.bmj.com/site/about/guidelines.xhtml

		assessing outcomes) and how	
	11b	If relevant, description of the similarity of interventions	4
Statistical methods	12a	Statistical methods used to compare groups for primary and secondary outcomes	10
	12b	Methods for additional analyses, such as subgroup analyses and adjusted analyses	10
**Results**			
Participant flow (a diagram is strongly recommended)	13a	For each group, the numbers of participants who were randomly assigned, received intended treatment, and were analysed for the primary outcome	n/a
	13b	For each group, losses and exclusions after randomisation, together with reasons	n/a
Recruitment	14a	Dates defining the periods of recruitment and follow-up	n/a
	14b	Why the trial ended or was stopped	n/a
Baseline data	15	A table showing baseline demographic and clinical characteristics for each group	n/a
Numbers analysed	16	For each group, number of participants (denominator) included in each analysis and whether the analysis was by original assigned groups	n/a
Outcomes and estimation	17a	For each primary and secondary outcome, results for each group, and the estimated effect size and its precision (such as 95% confidence interval)	n/a
	17b	For binary outcomes, presentation of both absolute and relative effect sizes is recommended	n/a
Ancillary analyses	18	Results of any other analyses performed, including subgroup analyses and adjusted analyses, distinguishing pre-specified from exploratory	n/a
Harms	19	All important harms or unintended effects in each group (for specific guidance see CONSORT for harms)	n/a
**Discussion**			
Limitations	20	Trial limitations, addressing sources of potential bias, imprecision, and, if relevant, multiplicity of analyses	n/a
Generalisability	21	Generalisability (external validity, applicability) of the trial findings	n/a
Interpretation	22	Interpretation consistent with results, balancing benefits and harms, and considering other relevant evidence	n/a
**Other information**			
Registration	23	Registration number and name of trial registry	1
Protocol	24	Where the full trial protocol can be accessed, if available	n/a
Funding	25	Sources of funding and other support (such as supply of drugs), role of funders	12

*We strongly recommend reading this statement in conjunction with the CONSORT 2010 Explanation and Elaboration for important clarifications on all the items. If relevant, we also recommend reading CONSORT extensions for cluster randomised trials, non-inferiority and equivalence trials, non-pharmacological treatments, herbal interventions, and pragmatic trials. Additional extensions are forthcoming: for those and for up to date references relevant to this checklist, see www.consort-statement.org.

For peer review only - http://bmjopen.bmj.com/site/about/guidelines.xhtml

For peer review only

BMJ Open

Combining cognitive bias modification training (CBM) and transcranial direct current stimulation (tDCS) to treat binge eating disorder: study protocol of a randomised controlled feasibility trial

Journal:	*BMJ Open*
Manuscript ID	bmjopen-2019-030023.R2
Article Type:	Protocol
Date Submitted by the Author:	01-Aug-2019
Complete List of Authors:	Gordon, Gemma; King's College London, Section of Eating Disorders, Department of Psychological Medicine, Institute of Psychiatry, Psychology and Neuroscience,<br>Brockmeyer, Timo; University of Gottingen, Department of Clinical Psychology and Psychotherapy, Institute of Psychology<br>Schmidt, Ulrike; King's College London, Section of Eating Disorders, Department of Psychological Medicine, Institute of Psychiatry, Psychology and Neuroscience<br>Campbell, Iain; King's College London, Section of Eating Disorders, Department of Psychological Medicine, Institute of Psychiatry, Psychology and Neuroscience
**Primary Subject Heading**:	Mental health
Secondary Subject Heading:	Diabetes and endocrinology, Public health
Keywords:	Eating disorders < PSYCHIATRY, transcranial direct current stimulation, cognitive bias modification, binge eating, treatment

SCHOLARONE™
Manuscripts

07.07.2019 V1

Combining cognitive bias modification training (CBM) and transcranial direct current stimulation (tDCS) to treat binge eating disorder: study protocol of a randomised controlled feasibility trial

Gemma Gordon[1], Timo Brockmeyer[2], Ulrike Schmidt[1], Iain Campbell[1]

[1]Section of Eating Disorders, Department of Psychological Medicine, Institute of Psychiatry, Psychology and Neuroscience, King's College London, London, United Kingdom

[2]Department of Clinical Psychology and Psychotherapy, Institute of Psychology, University of Goettingen, Goettingen, Germany

Correspondence: Gemma Gordon, Section of Eating Disorders, Department of Psychological Medicine, Institute of Psychiatry, Psychology and Neuroscience, King's College London, London, United Kingdom; E-mail: gemma.gordon@kcl.ac.uk

Key words: cognitive bias modification, transcranial direct current stimulation, eating disorders, binge eating, treatment

Word count (abstract): 290 **Word count (manuscript): 5,908**

For peer review only

ABSTRACT

Introduction: Binge Eating Disorder (BED) is a common mental disorder, closely associated with obesity. Existing treatments are only moderately effective with high relapse rates, necessitating novel interventions. This paper describes the rationale for, and protocol of, a feasibility randomised controlled trial (RCT), evaluating the combination of transcranial Direct Current Stimulation (tDCS) and a computerised cognitive training, namely Approach Bias Modification training (ABM), in patients with Binge Eating Disorder who are overweight or obese. The aim of this trial is to obtain information that will guide decision making and protocol development in relation to a future large-scale RCT of combined tDCS + ABM treatment in this group of patients, and also to assess the preliminary efficacy of this intervention. **Methods and analysis:** 66 participants with DSM-5 diagnosis of BED and a body mass index (BMI) of >25 kg/m^2 will be randomly allocated to one of 3 groups: ABM + real tDCS; ABM + sham tDCS or a waitlist control group. Participants in both intervention groups will receive 6 sessions of ABM + real/sham tDCS over 3 weeks; engaging in the ABM task while simultaneously receiving bilateral tDCS to the dorsolateral prefrontal cortex. ABM is based on an implicit learning paradigm in which participants are trained to enact an avoidance behaviour in response to visual food cues. Assessments will be conducted at baseline, post-treatment (3 weeks), and follow-up (7 weeks post-randomisation). Feasibility outcomes assess recruitment and retention rates, acceptability of random allocation, blinding success (allocation concealment), completion of treatment sessions and research assessments. Other outcomes include eating disorder psychopathology and related neurocognitive outcomes (i.e. delay of gratification and inhibitory control), BMI, other psychopathology (i.e. mood), approach bias towards food, and surrogate endpoints (i.e. food cue reactivity, trait food craving, and food intake).

Ethics and dissemination: This study has been approved by the North West - Liverpool East Research Ethics Committee. Results will be published in peer-reviewed journals.

Trial registration number: ISRCTN35717198

Strengths and limitations of this study

- The ICARUS study is the first randomised controlled feasibility trial of multi-session transcranial Direct Current Stimulation (tDCS) combined with Cognitive Bias Modification training (CBM) for adults with Binge Eating Disorder.
- ICARUS will compare [tDCS + CBM] vs. [sham tDCS + CBM] and a wait-list control group.
- ICARUS is designed to answer questions about the efficacy of the treatments tested.
- Results would need to be replicated in a larger trial before recommendations for tDCS + CBM as a treatment adjunct for patients receiving outpatient treatment for BED can be made.

For peer review only - http://bmjopen.bmj.com/site/about/guidelines.xhtml

INTRODUCTION

Binge Eating Disorder (BED) is the most prevalent eating disorder (ED) worldwide, with 1-3% of the general population meeting diagnostic criteria[1,2]. Binge eating is a core symptom, characterised by consumption of large amounts of food, a sense of loss of control, and significant distress. Nearly 80% of those with lifetime BED have a comorbid psychiatric disorder, such as mood, anxiety, substance use disorders or another ED[2]. Due to the lack of compensatory behaviours (e.g. vomiting, excessive exercising), BED is often accompanied by, or leads to, obesity and associated physical complications[3,4]. In the general population, approximately 30-42% of people with BED are obese[2,5,6]. Around 30% of treatment-seeking obese people[7–9] and up to 47% of bariatric surgery candidates have full or partial BED[1,10,11]. Whilst BED itself has considerable individual and societal costs[12], the combination of BED and obesity is associated with more severe obesity, greater medical and psychiatric comorbidity, greater functional impairment and perinatal complications[12–15]. Treatments for BED and obesity are sub-optimally effective, with cognitive behavioural therapy (CBT)[16] and some medications[17] reducing binge eating and related psychopathology[1], and approximately 50-60% of patients achieving abstinence from bingeing at the end of treatment[18] with some sustained cessation at follow-up[19]. However, drop-out rates in established BED treatments reach 12-34%, and 30%-50% of BED patients relapse in long-term follow-ups[20–22], indicating that a substantial proportion do not maintain binge eating remission. Lisdexamfetamine[23] and topiramate[24] also reduce weight in the short-term but have considerable side effects[25], and their longer term efficacy is uncertain. Thus, there is a need for novel treatment developments.

The aetiology of BED is widely seen as multi-factorial. Emerging neurobiological models emphasise both the role of stress in the onset and maintenance of the disorder[26,27], and the development of addiction-like features; craving, tolerance and binge escalation over time[28,29], impulsivity and compulsivity, alterations in executive function and attention[30] and reward-related decision making[31].

Upon encountering images of high-calorie food, BED patients report enhanced reward sensitivity and exhibit stronger medial orbitofrontal cortex responses compared to healthy controls and participants with bulimia nervosa[32]. In individuals with obesity, who may or may not have BED, activation in the ventral striatum (part of the reward system) has been found to be higher compared to normal-weight controls [33], in tandem with a more pronounced approach bias towards appetising food images [34,35], leading to greater likelihood of consumption. Furthermore, poor reward-related decision making behaviour may be a maintaining factor in obesity[36]. Converging data using different methodologies, such as brain imaging, eye tracking, and behavioural test paradigms [37] have found that patients with BED demonstrate a higher arousal rate in response to food stimuli, a concurrent motor plan to start eating, a higher reward sensitivity, and greater inhibitory deficits as compared to individuals without BED[32,38,39]. Those with obesity and BED (compared to obesity alone) have demonstrated that their attentional bias to food images held higher motivational value[40], and responded more to high calorie food images in sites of cognitive planning of motor movements, driven by emotions, which may reflect impulsive tendencies in the face of a binge-eating trigger. This tendency to approach and consume palatable food items may thus be compounded by a greater sensitivity to reward and a decreased capacity to inhibit action tendencies. This is corroborated by the recent finding that individuals with BED or Bulimia Nervosa

For peer review only - http://bmjopen.bmj.com/site/about/guidelines.xhtml

(BN) show higher food cue reactivity (increased cravings) when exposed to visual food cues compared to healthy controls [41]. Such accumulating evidence of BED as a unique diagnostic group situates it as a distinct phenotype within the obesity spectrum that is characterised by increased impulsivity [42].

Conventional treatments of BED, such as CBT may not be best suited to target highly automatic cognitive processes that occur at an early stage in information processing and that are considered to contribute to food craving and associated maladaptive cognitions/behaviours. Two "brain-directed" treatments may provide an avenue for modifying these processes: Approach Bias Modification training (ABM) and transcranial Direct Current Stimulation (tDCS).

Approach Bias Modification training (ABM) is a form of cognitive bias modification training (CBM) that aims to retrain approach bias tendencies (reach out towards) into avoidance ones (move away from)[43] regarding stimuli such as appetitive cues. Participants are systematically trained to show an avoidance movement in response to illness-related rewarding stimuli (e.g. food or alcohol) on a computer screen. ABM techniques have shown potential in several pilot and large-scale randomised controlled studies to treat alcohol[44] and tobacco[45] addictions, and to reduce consumption of cannabis[46] and unhealthy foods[47,48]. ABM has also yielded promising results in people with high levels of food craving and in bulimic eating disorders, including BED[49,50]. However, mixed results in empirical studies across these domains[51,52] raise methodological issues of ABM studies to date, such as low statistical power and suboptimal choice (or absence) of control groups[53] and administration of single versus multiple training sessions.

Transcranial Direct Current Stimulation (tDCS) is a form of non-invasive brain stimulation (NIBS) that has been used as a treatment adjunct for a range of psychiatric disorders, such as depression, schizophrenia and addictions[54–56]. Preliminary evidence suggests that tDCS and other forms of non-invasive brain stimulation are promising tools to reduce food craving, ED symptoms and body weight in bulimic EDs, including BED, and obesity[57]. Additionally, some studies indicate that NIBS may reduce depression/stress levels and improve reward-based decision making in ED patients[58]. A frequent stimulation target is the dorsolateral prefrontal cortex (dlPFC) which plays a major role in cognitive-inhibition, emotion regulation, and reward processing[58–61]. Although precise mechanisms of action of tDCS have yet to be understood, a key hypothesis in relation to BED is that enhancing dlPFC activity via tDCS alters the reward-cognition balance towards facilitation of cognitive control and suppression of reward-related mechanisms driving food craving/overeating[62].

If given concurrently (i.e. 'online training'), NIBS is reported to boost the effects of cognitive training on the reduction of cognitive biases and the improvement of response inhibition[63]. NIBS may enhance synaptic strength in neuronal pathways activated by cognitive training, amplifying effects of training, and thus cognitive bias modification efficacy [64]. As the effectiveness of tDCS may thus be improved by pairing administration with a cognitive task inducing activity in the target brain region[65–67], such combined treatment interventions have been investigated among alcohol dependent inpatients (ABM and tDCS)[68], and to enhance inhibitory control related to food consumption (Go/No-Go Task and tDCS)[65]. The insignificant findings from these studies warrant commentary that to date, studies that have found positive effects of tDCS have either included obese participants or have had multisession protocols[65,69–71]. As this study incorporates both

For peer review only - http://bmjopen.bmj.com/site/about/guidelines.xhtml

aspects, it is optimally designed to yield significant results.

In light of both the individual and societal burden incurred by the rising prevalence of BED and obesity, research interventions informing treatments that lead to stable and long-lasting remission are of critical importance, and novel therapies may play a role in serving as adjuncts to treatment as usual, to enhance improvement in clinical outcomes obtained from engaging with eating disorder treatment services. This research trial is the first to combine two promising novel intervention strategies in an integrated treatment and will yield important findings to shape future clinical trials. The intervention conditions of this feasibility study will involve 6 sessions of concurrent ABM and real or sham tDCS over 3 weeks, and will assess participant acceptability and dropout rates at this treatment frequency and duration. Additionally, the frequency of participants' ED symptoms and other outcomes related to general psychopathology and neurocognition will be measured before and after the study interventions to assess treatment success. In summary, this proof-of-concept and feasibility study will establish the utility of concurrent ABM + real tDCS in improving clinical outcomes in participants with BED, compared to ABM + sham tDCS, and a wait-list control group.

STUDY AIMS

In line with established recommendations for outcomes of feasibility trials[72], which at present are supported by the National Institute for Health Research, the primary aim is to assess the feasibility of using concurrent ABM + real tDCS compared to concurrent ABM + sham tDCS as a potential adjunct to treatment as usual (TAU) in this patient population, and acquire key information to inform the development of a large-scale randomised sham-controlled trial (RCT).

The specific objectives of the proposed feasibility study are to:

1. Establish the feasibility of conducting a large-scale RCT of ABM + tDCS in patients with BED by assessing recruitment, attendance, and retention rates;
2. Determine the practicality of administering both ABM and tDCS simultaneously;
3. Determine the best instruments for measuring outcomes in a full trial by examining the quality, completeness, and variability in the data;
4. Estimate the treatment effect sizes and standard deviations for outcome measures to inform the sample size calculation for a large-scale RCT;
5. Evaluate whether the treatment is operating as it is designed by analysing process measures, such as within-session visual analogue scales (VAS) of key ED symptoms;
6. Determine whether patients with BED evaluate concurrent ABM + tDCS as acceptable and credible;
7. Obtain information about patients' willingness to undergo random allocation to ABM paired with either real or sham tDCS administration, or the wait-list control condition.

A secondary aim is to investigate the potential efficacy of concurrent delivery of both forms of treatment on binge eating disorder.

This will involve evaluating if:

1. Concurrent sessions of ABM + real tDCS are superior to ABM + sham tDCS and to wait-list control in terms of frequency of objective binge eating episodes, food cue reactivity, food craving, food intake, eating disorder psychopathology and mood.

For peer review only - http://bmjopen.bmj.com/site/about/guidelines.xhtml

2. Concurrent ABM + tDCS is superior to the two other conditions in having an effect on the targeted neurocognitive mechanism (approach bias for high calorie food) and related neurocognitive parameters (i.e. impulsivity, delayed gratification, emotional regulation).
3. Concurrent ABM and sham tDCS is superior to the waitlist control in eliciting therapeutic effects on the aforementioned clinical outcomes and neurocognitive mechanisms, yet demonstrates an efficacy level below that of concurrent ABM and real tDCS.

METHODS AND ANALYSIS

This study protocol has been written according to the SPIRIT statement (Standard Protocol Items for Randomised Trials)[73] and the CONSORT 2010 statement (Consolidated Standards of Reporting Trials)[72].

Study design

The ICARUS trial (Investigating Concurrent Approach Bias Modification Training and Transcranial Direct Current Stimulation in Binge Eating Disorder) is an exploratory randomised controlled feasibility trial with three parallel treatment conditions; ABM + real tDCS, ABM + sham tDCS and wait-list control. All participants across the two intervention groups will receive a treatment protocol of 6 sessions of ABM + real/sham tDCS conducted over 3 weeks. The comparator groups of a wait-list control and ABM + sham tDCS are necessary to evaluate the potential effect of real versus sham tDCS in participants with BED. The wait-list control group will be examined at the same time points to control for the possibility that improvements in the intervention groups are simply due to regression to the mean, spontaneous remission or other non-specific time effects. Any participants who are engaging in treatment for their ED will continue with TAU, and thus this selection of comparators is deemed acceptable. Within treatment session measures will involve visual analogues scales evaluating mood, stress and eating disorder symptoms. Assessments will be conducted 3 times during the study; at baseline, post-treatment (week 3), and at follow-up (week 7).

Participants

Inclusion criteria entail: (1) male and female community-dwelling adults (aged 18-70); (2) overweight or obese according to WHO criteria (BMI>25 kg/m^2)[74]; (3) a diagnosis of full-syndrome or sub-threshold Binge Eating Disorder according to the DSM-5[75]L; (4) fluency in English; (5) normal or corrected to normal vision .
Exclusion criteria entail: (1) all known contraindications to tDCS[76]; (2) pregnancy; (3) a current significant/unstable medical or psychiatric disorder needing acute treatment in its own right; (4) a lifetime diagnosis of substance dependence, psychosis, bipolar disorder or borderline personality disorder; (5) taking psychotropic medication other than a stable dosage of selective serotonin reuptake inhibitors (SSRI) for at least 14 days prior to study enrolment; (6) allergies to any of the foods presented in the study; (7) smoking >10 cigarettes per day; (8) drinking >3-4 units (men) or 2-3 units (women) of alcohol per day. In line with the CONSORT guidelines[77,78], we will record the number and reasons for any participants we must exclude, or any who decline consent or withdraw from the study.

Sample size

For peer review only - http://bmjopen.bmj.com/site/about/guidelines.xhtml

As ICARUS is a feasibility study, an a priori sample size calculation is not necessary. Rather, its aim is to provide effect sizes on which future large-scale studies can be powered. Total study sample sizes of n=24 to n=50 have been recommended for feasibility trials with a primary outcome measured on a continuous scale, mainly because estimates of the standard deviation for normally distributed variables tend to stabilise around this size[79,80]. We have chosen a target end study sample size of n=60, (i.e. exceeds the upper end recommended for feasibility trials). However, assuming the attrition to follow-up rate is a = 0.10 (as found in previous eating disorder trials [81,82]) and applying an attrition correction factor of 1/(1-a), we will recruit an actual sample size of 66, i.e., 22 participants per group.

Randomisation

After the baseline assessment, participants will be allocated to one of three conditions at random to receive 6 sessions of either concurrent ABM + real tDCS or ABM + sham tDCS, or no intervention in the wait-list control condition. As a proportion of participants recruited from an outpatient eating disorder clinic will be on a waiting list to receive treatment at the time of enrolment in this study and may commence treatment shortly after study enrolment, this study will not seek to balance groups in terms of therapy engagement or medication usage. Participants in the wait-list control group will be offered the opportunity to receive ABM + real tDCS after the end of the follow-up. Participants will be individually randomised on a 1:1:1 ratio to the intervention or control groups in equal numbers. The generation and implementation of the randomisation sequence will be conducted independently from the trial team through a randomisation administrator who is not involved in any recruitment or research activity related to the ICARUS study. Online randomisation software (Sealed Envelope, London, UK) will be used for this purpose. Upon participant enrolment, the researcher will contact the randomisation administrator, who will inform this researcher in charge of carrying out the intervention of the participant's allocation via phone or email.

Blinding

Double blinding is implemented only for the intervention group cohorts of the trial. The research assessor will remain blind to each participant’s tDCS assignment within the two intervention conditions until after the participant has completed the follow-up assessment. This double blinding protocol will be ensured via administration of the tDCS (NeuroConn DC-STIMULATOR PLUS) using “study mode”. This involves a five-digit numerical code unique to each patient will be inputted into the device prior to the participant’s testing session, that will initialise either sham or real (active) stimulation. The tDCS administrator and participants will remain blind to tDCS stimulation type throughout the study. Set-up of the randomisation codes and programming of the tDCS device will be performed by an investigator not involved in the trial. To assess blinding success, each participant and the researcher will be asked to guess the treatment allocation at the end of the 6 treatment sessions and to indicate how certain they are of this guess. The study group allocation will be revealed to the participant after their follow-up assessment. In the event of a reported change in a participant’s medication, or a new clinical diagnosis made during their study participation, the early unblinding of study condition for an intervention group participant will be permissible. The trial database will be maintained ‘blind’ until the point of study data analyses.

For peer review only - http://bmjopen.bmj.com/site/about/guidelines.xhtml

Recruitment

The study will take place at the Institute of Psychiatry, Psychology and Neuroscience (IoPPN), King's College London (KCL), UK. Participants will be recruited from the Eating Disorders Service at the South London and Maudsley NHS Foundation Trust, from the KCL research recruitment webpage and social media account, and via posters placed on notice boards on KCL campuses. Participants who have previously taken part in research at the KCL Eating Disorders Unit and who have consented to be informed of future studies may also be contacted. The ICARUS study will also be advertised on the Beat (National Eating Disorders Association) website, callforparticipants.com and www.mqmentalhealth.org. Potential participants will receive written and verbal study information and will be screened for eligibility. Eligible participants will provide informed written consent for study participation as a prerequisite for enrolment (See Appendices A and B).

Procedure

Figure 1 illustrates the timeline of the study procedures. All participants will partake in assessments at each of the three measurement points; baseline, post-treatment, and follow-up. Each assessment will comprise of an in-person study visit with tasks and measures, and online/hardcopy questionnaires and scales to be completed at home by the participant within 36 hours following the study visit. Table 1 details the tasks and measures allocated to each assessment and training visit. After the baseline assessment, participants are randomised to one of 3 groups; (1) ABM + real tDCS, (2) ABM + sham tDCS, or a waitlist control group (CG). Participants allocated to an intervention group will be offered 6 sessions of ABM + real/sham tDCS across 3 weeks. All study participants may receive TAU e.g. if they are currently engaged in outpatient treatment for their ED. The control group will not receive any study intervention, and any participants receiving outpatient services treatment for their ED will continue TAU during this 3 week period. The post-treatment assessment will be conducted on all participants after the 6th (final) session of ABM + real/sham tDCS for the intervention groups, and 3 weeks after the baseline assessment for the control group. The follow-up assessment will be conducted 28 days after the end of treatment, i.e. 7 weeks post-randomisation. A follow-up period is included because, if the effects of the intervention result from increased neuroplasticity, behavioural changes may need time to emerge. Assessing the longevity of favourable clinical outcomes beyond the treatment period is also relevant to the objectives of this feasibility study. The researcher conducting the assessment and testing sessions will remain blind to the study condition of intervention group participants until the follow-up has been completed.

Outcome assessment

Measures of feasibility, safety and adherence will be collected throughout the study. Outcomes related to ED symptoms, general psychopathology and neurocognition will be measured before and after the study intervention to assess treatment success. Each assessment session will be split into an in-person visit and at-home component to accommodate time constraints and minimise disruption to task performance due to participant fatigue. The in-person assessment measures will take approximately 75 minutes to complete, and the online questionnaires will take approximately 45 minutes.

Outcome Measures

For peer review only - http://bmjopen.bmj.com/site/about/guidelines.xhtml

Feasibility outcomes

As this is a feasibility study, an extensive range of outcome measures are included to help determine which are most sensitive to detecting a treatment effect. This will enable us to determine primary outcome(s) for a future large-scale RCT. However, based on previous research[49], the Eating Disorder Examination Questionnaire (EDE-Q) is anticipated to be a key outcome measure.

Intervention/service related outcomes

Feasibility outcomes include recruitment, attendance and retention rates, and acceptability of treatment by participants. Patients' acceptance of study interventions will be assessed by measuring treatment dropout rates and via the treatment tolerance and acceptability questionnaires. An interview assessment of treatment experience will be conducted with 20 participants after the follow-up is completed. 10 participants from each intervention group will be invited to provide feedback on their initial expectations and experiences of the ABM + real/sham tDCS treatments, perceived strengths and weaknesses of the treatment they received, and suggestions for improvements in procedures. Interviews will be recorded, transcribed and analysed using thematic analysis. This will allow future studies to consider patients' feedback in the development of research and clinical protocols of concurrent ABM and tDCS.

Clinical outcomes

Eating disorder and related psychopathology
(a) Eating Disorder Examination (EDE-Q)[83]: The EDE-Q is a widely used measure of eating-disordered behaviour and is widely regarded as the instrument of choice for the assessment of EDs. This will be administered at baseline, post-assessment and follow-up.

b) Body Mass Index (BMI (kg/m2)): This assessment of body composition provides accurate estimates of body fat percentages in adults, where sex and age are factored into the analysis measuring height and weight[84]. To calculate BMI, height and weight measurements will be obtained by the researcher at baseline, post-assessment and follow-up.

(c) *Approach bias assessment tasks*
To identify the most sensitive method of assessing change in approach bias towards high-calorie food items, two different computerised measures of approach bias will be used. In the Food Approach-Avoidance Task (F-AAT)[85] participants are shown colour photographs of high-calorie, palatable foods such as chocolate, cake, and pizza, and non-food household and office items such as sponges and stationary on a computer-screen[86]. They are instructed to approach and avoid these stimuli by moving a joystick toward themselves (approach) or away from themselves (avoidance). In the Stimulus Response Compatibility Task (SRC)[87], participants perform a symbolic movement by making a manikin image walk toward (approach) or away from stimuli (avoidance). See Appendix C for more detailed information. Both of these tasks will be administered at baseline, post-assessment and follow-up.

(d) Food choice attitudes/behaviour
The Food Choice Task[88–90] is a computer-based paradigm that measures responses to images of

For peer review only - http://bmjopen.bmj.com/site/about/guidelines.xhtml

foods to assess food attitudes and characteristics of eating behaviour. Participants rate images of food on a computer screen according to healthiness as well as tastiness. Based on these ratings they are then offered a choice between a food that they consider "neutral" and a series of other foods. See Appendix C for more detailed information. This task will be performed at baseline and post-assessment.

(e) *Food craving after cue exposure task*

The Food Challenge Task (FCT)[41] will be used to examine cue-induced food craving. In this task, participants rate their state food craving using the Food Cravings Questionnaire State Version[91,92] before and after being presented with a video on a computer screen of foods shown to be highly appetising[93]. See Appendix C for more detailed information. This task will be performed at baseline and post-assessment.

(f) *Trait food craving*

Three questionnaires will be used to comprehensively assess mechanisms implicated in trait food craving. The Food Cravings Questionnaire Trait Version - reduced (FCQ-T-r)[94] is a 15 items only reduced version of a self-report questionnaire that measures trait levels of craving for food. The 21-item Power of Food Scale (PFS)[95] scale assesses the psychological influence of the mere presence or availability of food. It measures appetite for, rather than consumption of, palatable foods, at three levels of food proximity (food available, food present, and food tasted). The Yale Food Addiction Scale Version 2.0 (YFAS 2.0)[96] reflects the current diagnostic understanding of addiction to further investigate the potential role of an addictive process in problematic eating behaviour[75,97–101]. See Appendix C for more detailed information. Each of these scales will be administered at baseline, post-assessment and follow-up.

(g) *Food intake in a bogus taste test*

During the bogus taste test[102], participants will be instructed to rate and optionally consume highly palatable high-calorie food items presented in 3 bowls. See Appendix C for more detailed information. This task will be performed at baseline and post-assessment.

(h) *Increased preference for delayed rewards*

The Delay Discounting Task with Money and Food[103] examines whether small amounts of food would be discounted more steeply than money, as occurs with larger amounts. See Appendix C for more detailed information. This will be administered at baseline, post-assessment and follow-up.

(i) *Inhibitory control*

The cued Go/No-Go computer task is a classic test of executive function, requiring effortful response inhibition, and measures impulse control by the ability to inhibit instigated, prepotent responses. A food specific go/no-go task[104] measures impulsivity and response inhibition with respect to food and nonfood items. The Stop Signal Task (SST)[105]measures inhibitory control. Participants are required to engage in a computer task but withhold their response in the presence of a stop signal. An adaptation of the food version of the SST[106] will facilitate a comparison of responses between food and non-food categories. See Appendix C for more detailed information. Both of these tasks will be performed at baseline, post-assessment and follow-up.

For peer review only - http://bmjopen.bmj.com/site/about/guidelines.xhtml

Mood and emotion regulation

The Emotion Regulation Questionnaire (ERQ)[107] is designed to measure respondents' tendency to regulate their emotions regarding cognitive reappraisal and expressive suppression. The Positive and Negative Affect Schedule (PANAS)[108] measures the degree of positive or negative affect experienced "right now" in the current study. The Depression, Anxiety and Stress Scale (DASS-21)[109] evaluates mood, anxiety and stress levels over the previous week. See Appendix C for more detailed information. All of these measures will be administered at baseline, post-assessment and follow-up.

Within session measures

Within each training session, i.e. immediately before and after the ABM + real/sham tDCS procedure the researcher will administer paper-based Visual Analogue Scales (VAS) assessing current hunger, feeling of fullness, urge to eat, urge to binge eat, feeling low, level of tension, level of stress, level of anxiety, and any discomfort due to tDCS and ABM in the training session. See Appendix C for more detailed information.

Intervention

In both intervention groups participants will receive six sessions of concurrent ABM and real or sham tDCS which will be delivered twice a week for three weeks. A researcher trained in tDCS administration will deliver the training sessions.

Rationale for number of sessions:

Treatment parameters for interventions of ABM and tDCS separately in psychiatric disorder research have not yet been standardised and vary from 1-12 sessions across a timeframe of days to multiple weeks. Mixed results regarding optimal frequency of ABM sessions and related forms of cognitive bias modification has been reported[110]. A maximum accumulative effect of modification efficacy at 6 sessions has been found for approach bias modification for alcohol dependence[44]. While there is a similar paucity of specifications for treatment parameters within tDCS, multisession NIBS interventions are significantly more effective at reducing cravings and strengthening the ability to refrain from food consumption than single session protocols in eating disorders and obesity[111]. As a single session of tDCS on patients with BED was found to reduce craving and caloric intake[59], it was hypothesised that repeated administration of tDCS would enhance this effect and may decrease binge eating frequency.

Within session safety procedures

The participant's blood pressure and heartrate will be taken by the researcher immediately before and after the session. While the participant is comfortably seated, the tDCS and ABM will be administered at the same time, i.e. participants will engage in ABM training whilst receiving tDCS. Each session will last 20 minutes. The ABM training will start 5 minutes after the start of the brain stimulation. ABM training will take place over 10 minutes and tDCS will then continue for a further 5 minutes. Participants will be reminded that they have the option to withdraw immediately and terminate their participation in the study if they experience discomfort during tDCS administration, or if they wish to withdraw for any reason that they may or may not wish to disclose.

For peer review only - http://bmjopen.bmj.com/site/about/guidelines.xhtml

Approach bias modification training

The ABM programme will use an implicit learning paradigm, based on a modified version of the Food Approach/Avoidance Task (AAT)[85,112–114]. In this task, participants are shown pictures of food and control (i.e. neutral office) items. They are required to pull (pictures grow bigger) or push (pictures grow smaller) a joystick in response to the outer frame of the picture (round vs. rectangular), irrespective of the picture content. The training version of the Food-AAT utilises an implicit learning paradigm by presenting all food pictures in the "push" (i.e. avoid) format. The study procedure for ABM administration is aligned with previous research[50,115].

Transcranial direct current stimulation

TDCS (both real and sham) will be delivered using a NeuroConn® DC-STIMULATOR PLUS device at a constant current of 2 mA (with a 10-second fade in/out) using two 25cm² surface sponge electrodes soaked in a sterile saline solution (0.9% sodium chloride). The anode will be placed over the right dorsolateral prefrontal cortex (dlPFC) and the cathode over the left dlPFC. This montage has been used in sham-controlled studies on food craving, bulimia nervosa and BED[59]. The stimulation site will be located using the Beam F3 calculation method, which is based on the International 10-20 system. TDCS can occasionally result in mild discomfort during administration (i.e., tingling or itching sensation, a slightly metallic taste, occasional redness at the site of the electrodes). Fatigue, headache, nausea and insomnia have been reported as potential adverse reactions[116]. Participants who are at-risk for adverse effects[76] will be excluded from the study at the screening stage.

Data analysis

Data will be analysed with the Statistical Package of Social Sciences (SPSS). Feasibility outcome data will be analysed with appropriate summary statistics. To determine quality, completeness, and variability of the clinical outcome data, descriptive statistical analyses and graphical methods will be used. Intent-to-treat analyses will be performed. The size of the treatment effect on each outcome measure will be the difference in outcome data between those in the two treatment conditions and control condition at post-assessment and follow-up. Group differences will be estimated using linear mixed effects regression models, controlling for the baseline level of the outcome. The goal here is not to determine significant group differences but to establish a suitably precise effect size for the primary outcome at the post treatment assessment. This estimate will be used to guide the sample size of a future efficacy trial. Correlational analyses may be computed to analyse relationships between outcome variables and influences of potential covariates such as demographic variables, i.e. gender, age, BMI and clinical variables, i.e. start/stopping of psychotherapy, psychotropic medication and presence of comorbidities. Outcome data already obtained for participants who discontinue or deviate from the intervention protocol will be kept and analysed.

PATIENT AND PUBLIC INVOLVEMENT

Patients and/or public were not involved in the study design process, however we will obtain 20 intervention participants' qualitative views on their treatment experience in this study to inform future clinical trials.

For peer review only - http://bmjopen.bmj.com/site/about/guidelines.xhtml

ETHICS AND DISSEMINATION

Data management and data monitoring

Participant data will be anonymised and all anonymised data will be stored electronically on a password protected computer at the IoPPN. All trial data will be stored in line with the General Data Protection Regulation (GDPR) 2018. Hard copies of participant-related data (i.e. GP letters) will be kept in locked cabinets at the IoPPN, King's College London. The final trial data set will not be accessed by anyone other than members of the research team.

Data will be stored on manual files, university and laptop computers. There will be no personal data stored on laptop computers. Confidentiality and anonymity of all personal data will be retained throughout the entire study. Manual files will be securely locked in a lockable filing cabinet, and all electronic files will be password protected. Identifying information will be removed from the data, stored separately and replaced with a numeric identification code. All participants will be allocated a numeric code, which will be used to identify their data. The master list of names which correspond to each participant's numeric identification code will be stored electronically and will be password protected. This information will only be accessible to key researchers involved in the study.

The online component of the assessment will use Online Surveys software (formerly BOS). King's College London uses this software for large scale surveys, and it is fully compliant with UK data protection laws. Participants will be emailed the link after the in-person component of each assessment session, and instructed to complete the second online component of the assessment within 36 hours. Participants will also have the option to receive and complete a hard copy version of the questionnaires with a stamped addressed envelope to post back to the study researcher at the IoPPN.

It is intended that the results of this feasibility study will be reported and disseminated at national and international conferences. Research findings may also be disseminated through internal newsletters and publications in collaboration with Beat, the UK's largest eating disorder charity.

Owing to the size and nature of this small-scale feasibility study, a data monitoring committee was not deemed to be required. There are no scheduled interim analyses and this trial may be prematurely discontinued by the Chief Investigator on the basis of new safety information.

Ethics and safety aspects

This study has been approved by the North West - Liverpool East Research Ethics Committee. This trial will be conducted in compliance with the study protocol, the Declaration of Helsinki, the principles of good clinical practice (ICH-E6 guideline), the ICH-E8 guideline and the principles of GCP and in accordance with all applicable regulatory requirements including but not limited to the UK policy framework for health and social care research. The ICARUS trial is registered with the International Standard Randomised Controlled Trial Number registry (number ISRCTN35717198). All participants will be asked by the study researcher to provide written informed consent prior to enrolment. Participants who are at-risk for adverse side effects[76]will be excluded from the study at the screening stage (i.e. such as those with pregnancy or epilepsy). Current safety parameters of tDCS administration regarding voltage amplitude and duration of brain stimulation sessions will be adhered to. Participants have the option to withdraw immediately and terminate their participation in the study if they experience discomfort during tDCS administration, or if they wish to terminate

For peer review only - http://bmjopen.bmj.com/site/about/guidelines.xhtml

their participation for any other reason that they may or may not wish to disclose. After each training session, participants will complete the tolerance, discomfort and side effects questionnaire to report any adverse effects of the intervention training session. The researcher will record this description of any reported adverse effects, and record the severity and duration of symptoms and how the adverse effect was managed at the following training session. If a participant reports a new clinical diagnosis or change in medication during their involvement in the study, a decision regarding their continued participation in the study will be made by the research team and withdrawal of the participant may be deemed necessary. Standard King's College London insurance and NHS indemnity arrangements apply to this study. To promote study adherence, upon completion of the follow-up assessment, each participant will be reimbursed for their time, efforts and travel (£60 for assessments and up to £60 for travel expenses). Additionally, participants in the wait-list control group will be offered the opportunity to receive 6 sessions of ABM + tDCS after the follow-up.

DISCUSSION

The ICARUS study represents the first feasibility study that aims to exploit a synergistic therapeutic effect by combining two brain-directed interventions in a single treatment intervention for BED. The rising clinical need of individuals with BED is currently met with few available psychological and neuropharmacological treatment options[117]. Therefore, such research advancing the identification and validation of novel therapies is greatly warranted.

This paper delineates the protocol for a feasibility trial which will inform future studies (i.e., provide effect sizes for a large RCT) and contribute to the extant research advocating brain-directed interventions for BED. The protocol aligns with current parameters of tDCS administration used to treat BED and BN[58,59] and utilises a multitudes of measures to identify the most appropriate and sensitive tools to detect treatment induced changes across pathological and neurocognitive domains.

Pragmatic concerns related to the recruitment process entail ensuring a sufficient and consistent rate of participant enrollment to meet the target sample number within the allocated timeframe. Additionally, drop-out rates for CBT treatment among a BED cohort are moderately high (17–30%)[118], thus study adherence will need to be monitored, with a revision of incentives/ reimbursement if necessary. Participants who were randomly allocated to the wait list control may avail themselves of 6 sessions of ABM + real tDCS after they have completed the study, which may promote recruitment and participant retention. Documenting the management of these issues will help to inform the development of a future large-scale RCT of this combined treatment adjunct for BED.

To conclude, investigating novel treatments for BED is an imperative issue. Combining ABM with tDCS is the strategic amalgamation of two techniques that have already demonstrated therapeutic efficacy in their own right. This feasibility RCT will be the first to systematically assess the acceptability and efficacy of a noninvasive, safe and potentially effective treatment adjunct to other therapies, which will enhance the ability of healthcare services to provide optimal care to patients with BED.

For peer review only - http://bmjopen.bmj.com/site/about/guidelines.xhtml

Trial progress

Recruitment will commence in February 2018 and data collection is expected to be complete (including follow-up assessments) by April 2020. Any substantial protocol amendments will be communicated to investigators via email and to other parties as required. Amendments to the study protocol will be reported in publications reporting the study outcomes.

Author affiliations

[1] Section of Eating Disorders, Department of Psychological Medicine, Institute of Psychiatry, Psychology & Neuroscience, King's College London, London, UK

[2] Department of Clinical Psychology and Psychotherapy, Institute of Psychology, University of Goettingen, Goettingen, Germany

Acknowledgements Not applicable

Contributors GG, IC, US and TB were each involved in the study conception and design. GG drafted the study protocol and IC, US and TB reviewed the paper and informed subsequent drafts. GG, IC, US and TB approved the final protocol paper.

Authorship policy No professional writers were involved in this study protocol, nor will be involved in the study report write-up.

Funding The project is supported by the NIHR Biomedical Research Centre (BRC) at South London and Maudsley NHS Foundation Trust (SLaM) and King's College London (KCL). Gemma Gordon is supported by a BRC PhD studentship and Ulrike Schmidt is supported by an NIHR Senior Investigator Award and receives salary support from the NIHR Mental Health BRC at SLaM NHS Foundation Trust and KCL. The funder was not involved in the study design and writing of this trial protocol paper, and will not be involved in the collection and analysis of data, nor the writing of the study report. The funders will not have ultimate authority over these activities.

Disclaimer The views expressed are those of the author(s) and not necessarily those of the NHS, the NIHR or the Department of Health.

Competing interests None declared

Patient consent Informed consent will be obtained by the researcher.

Ethics approval Ethical approval was given by the North West - Liverpool East Research Ethics Committee (ref: 18/NW/0648).

Provenance and peer review Not commissioned; externally peer reviewed.

Data sharing statement Not applicable

Open access This is an open access article distributed in accordance with the terms of the Creative Commons Attribution (CC BY 4.0) license, which permits others to distribute, remix, adapt and build upon this work, for commercial use, provided the original work is properly cited. See: http://creativecommons.org/ licenses/by/4.0/

For peer review only - http://bmjopen.bmj.com/site/about/guidelines.xhtml

For peer review only

© Article author(s) (or their employer(s) unless otherwise stated in the text of the article) 2018. All rights reserved. No commercial use is permitted unless otherwise expressly granted.

REFERENCES

1 McCuen-Wurst C, Ruggieri M, Allison KC. Disordered eating and obesity: associations between binge-eating disorder, night-eating syndrome, and weight-related comorbidities: Disordered eating and obesity. *Annals of the New York Academy of Sciences* 2018;**1411**:96–105. doi:10.1111/nyas.13467

2 Kessler RC, Berglund PA, Chiu WT, *et al.* The Prevalence and Correlates of Binge Eating Disorder in the World Health Organization World Mental Health Surveys. *Biological Psychiatry* 2013;**73**:904–14. doi:10.1016/j.biopsych.2012.11.020

3 de Zwaan M. Binge eating disorder and obesity. *Int J Obes Relat Metab Disord* 2001;**25 Suppl 1**:S51-55. doi:10.1038/sj.ijo.0801699

4 Hutson PH, Balodis IM, Potenza MN. Binge-eating disorder: Clinical and therapeutic advances. *Pharmacol Ther* 2018;**182**:15–27. doi:10.1016/j.pharmthera.2017.08.002

5 Cossrow N, Pawaskar M, Witt EA, *et al.* Estimating the Prevalence of Binge Eating Disorder in a Community Sample From the United States: Comparing DSM-IV-TR and DSM-5 Criteria. *J Clin Psychiatry* 2016;**77**:e968-974. doi:10.4088/JCP.15m10059

6 Kornstein SG, Kunovac JL, Herman BK, *et al.* Recognizing Binge-Eating Disorder in the Clinical Setting: A Review of the Literature. *Prim Care Companion CNS Disord* 2016;**18**. doi:10.4088/PCC.15r01905

7 Spitzer RL, Devlin M, Walsh BT, *et al.* Binge eating disorder: A multisite field trial of the diagnostic criteria. *International Journal of Eating Disorders* 1992;**11**:191–203. doi:10.1002/1098-108X(199204)11:3<191::AID-EAT2260110302>3.0.CO;2-S

8 Spitzer RL, Yanovski S, Wadden T, *et al.* Binge eating disorder: its further validation in a multisite study. *Int J Eat Disord* 1993;**13**:137–53.

9 Grucza RA, Przybeck TR, Cloninger CR. Prevalence and correlates of binge eating disorder in a community sample. *Comprehensive Psychiatry* 2007;**48**:124–31. doi:10.1016/j.comppsych.2006.08.002

10 Allison KC, Wadden TA, Sarwer DB, *et al.* Night eating syndrome and binge eating disorder among persons seeking bariatric surgery: prevalence and related features. *Obesity (Silver Spring)* 2006;**14 Suppl 2**:77S-82S. doi:10.1038/oby.2006.286

For peer review only

For peer review only - http://bmjopen.bmj.com/site/about/guidelines.xhtml

11 Adami GF, Gandolfo P, Bauer B, *et al.* Binge eating in massively obese patients undergoing bariatric surgery. *Int J Eat Disord* 1995;**17**:45–50.

12 Pawaskar M, Witt EA, Supina D, *et al.* Impact of binge eating disorder on functional impairment and work productivity in an adult community sample in the United States. *International Journal of Clinical Practice* 2017;**71**:e12970. doi:10.1111/ijcp.12970

13 Brown KL, LaRose JG, Mezuk B. The relationship between body mass index, binge eating disorder and suicidality. *BMC Psychiatry* 2018;**18**. doi:10.1186/s12888-018-1766-z

14 Watson HJ, Zerwas S, Torgersen L, *et al.* Maternal Eating Disorders and Perinatal Outcomes: A Three-Generation Study in the Norwegian Mother and Child Cohort Study. *J Abnorm Psychol* 2017;**126**:552–64. doi:10.1037/abn0000241

15 Raevuori A, Suokas J, Haukka J, *et al.* Highly increased risk of type 2 diabetes in patients with binge eating disorder and bulimia nervosa. *Int J Eat Disord* 2015;**48**:555–62. doi:10.1002/eat.22334

16 Palavras MA, Hay P, Filho CADS, *et al.* The Efficacy of Psychological Therapies in Reducing Weight and Binge Eating in People with Bulimia Nervosa and Binge Eating Disorder Who Are Overweight or Obese-A Critical Synthesis and Meta-Analyses. *Nutrients* 2017;**9**. doi:10.3390/nu9030299

17 Brownley KA, Berkman ND, Peat CM, *et al.* Binge-Eating Disorder in Adults: A Systematic Review and Meta-analysis. *Ann Intern Med* 2016;**165**:409–20. doi:10.7326/M15-2455

18 de Zwaan M, Herpertz S, Zipfel S, *et al.* Effect of Internet-Based Guided Self-help vs Individual Face-to-Face Treatment on Full or Subsyndromal Binge Eating Disorder in Overweight or Obese Patients: The INTERBED Randomized Clinical Trial. *JAMA Psychiatry* 2017;**74**:987–95. doi:10.1001/jamapsychiatry.2017.2150

19 Hilbert A, Bishop ME, Stein RI, *et al.* Long-term efficacy of psychological treatments for binge eating disorder. *Br J Psychiatry* 2012;**200**:232–7. doi:10.1192/bjp.bp.110.089664

20 Brownley KA, Berkman ND, Sedway JA, *et al.* Binge eating disorder treatment: a systematic review of randomized controlled trials. *Int J Eat Disord* 2007;**40**:337–48. doi:10.1002/eat.20370

21 Vocks S, Tuschen-Caffier B, Pietrowsky R, *et al.* Meta-analysis of the effectiveness of psychological and pharmacological treatments for binge eating disorder. *Int J Eat Disord* 2010;**43**:205–17. doi:10.1002/eat.20696

22 Wilson GT, Wilfley DE, Agras WS, *et al.* Psychological treatments of binge eating disorder. *Arch Gen Psychiatry* 2010;**67**:94–101. doi:10.1001/archgenpsychiatry.2009.170

23 McElroy SL, Hudson J, Ferreira-Cornwell MC, *et al.* Lisdexamfetamine Dimesylate for Adults with Moderate to Severe Binge Eating Disorder: Results of Two Pivotal Phase 3 Randomized Controlled Trials. *Neuropsychopharmacology* 2016;**41**:1251–60. doi:10.1038/npp.2015.275

24 Guerdjikova AI, Fitch A, McElroy SL. Successful Treatment of Binge Eating Disorder With Combination Phentermine/Topiramate Extended Release. *Prim Care Companion CNS Disord* 2015;**17**. doi:10.4088/PCC.14l01708

For peer review only

For peer review only - http://bmjopen.bmj.com/site/about/guidelines.xhtml

25 Arif H, Buchsbaum R, Weintraub D, *et al.* Patient-reported cognitive side effects of antiepileptic drugs: Predictors and comparison of all commonly used antiepileptic drugs. *Epilepsy & Behavior* 2009;**14**:202–9. doi:10.1016/j.yebeh.2008.10.017

26 Razzoli M, Pearson C, Crow S, *et al.* Stress, overeating, and obesity: Insights from human studies and preclinical models. *Neurosci Biobehav Rev* 2017;**76**:154–62. doi:10.1016/j.neubiorev.2017.01.026

27 Vannucci A, Nelson EE, Bongiorno DM, *et al.* Behavioral and neurodevelopmental precursors to binge-type eating disorders: support for the role of negative valence systems. *Psychological Medicine* 2015;**45**:2921–36. doi:10.1017/S003329171500104X

28 Smith DG, Robbins TW. The neurobiological underpinnings of obesity and binge eating: a rationale for adopting the food addiction model. *Biol Psychiatry* 2013;**73**:804–10. doi:10.1016/j.biopsych.2012.08.026

29 Devlin M. Binge Eating Disorder. In: *In K.D Brownell & B.T. Walsh (Eds.), Eating Disorders and Obesity, A Comprehemsive Handbook*. New York: : The Guildford Press 2017. 192–7.

30 Kessler RM, Hutson PH, Herman BK, *et al.* The neurobiological basis of binge-eating disorder. *Neuroscience & Biobehavioral Reviews* 2016;**63**:223–38. doi:10.1016/j.neubiorev.2016.01.013

31 Wu M, Brockmeyer T, Hartmann M, *et al.* Reward-related decision making in eating and weight disorders: A systematic review and meta-analysis of the evidence from neuropsychological studies. *Neurosci Biobehav Rev* 2016;**61**:177–96. doi:10.1016/j.neubiorev.2015.11.017

32 Schienle A, Schäfer A, Hermann A, *et al.* Binge-eating disorder: reward sensitivity and brain activation to images of food. *Biol Psychiatry* 2009;**65**:654–61. doi:10.1016/j.biopsych.2008.09.028

33 Stice E, Spoor S, Ng J, *et al.* Relation of obesity to consummatory and anticipatory food reward. *Physiol Behav* 2009;**97**:551–60. doi:10.1016/j.physbeh.2009.03.020

34 Brignell C, Griffiths T, Bradley BP, *et al.* Attentional and approach biases for pictorial food cues. Influence of external eating. *Appetite* 2009;**52**:299–306. doi:10.1016/j.appet.2008.10.007

35 Mehl N, Mueller-Wieland L, Mathar D, *et al.* Retraining automatic action tendencies in obesity. *Physiol Behav* 2018;**192**:50–8. doi:10.1016/j.physbeh.2018.03.031

36 Brockmeyer T, Simon JJ, Becker A, *et al.* Reward-related decision making and long-term weight loss maintenance. *Physiol Behav* 2017;**181**:69–74. doi:10.1016/j.physbeh.2017.09.008

37 Paslakis G, Kühn S, Grunert S, *et al.* Explicit and Implicit Approach vs. Avoidance Tendencies towards High vs. Low Calorie Food Cues in Patients with Obesity and Active Binge Eating Disorder. *Nutrients* 2017;**9**. doi:10.3390/nu9101068

38 Schag K, Teufel M, Junne F, *et al.* Impulsivity in binge eating disorder: food cues elicit increased reward responses and disinhibition. *PLoS ONE* 2013;**8**:e76542. doi:10.1371/journal.pone.0076542

39 Svaldi J, Naumann E, Trentowska M, *et al.* General and food-specific inhibitory deficits in binge eating disorder: Behavioral Inhibition in Bed. *International Journal of Eating Disorders* 2014;**47**:534–42. doi:10.1002/eat.22260

For peer review only - http://bmjopen.bmj.com/site/about/guidelines.xhtml

40 Aviram-Friedman R, Astbury N, Ochner CN, *et al.* Neurobiological evidence for attention bias to food, emotional dysregulation, disinhibition and deficient somatosensory awareness in obesity with binge eating disorder. *Physiol Behav* 2018;**184**:122–8. doi:10.1016/j.physbeh.2017.11.003

41 Meule A, Küppers C, Harms L, *et al.* Food cue-induced craving in individuals with bulimia nervosa and binge-eating disorder. *PLoS ONE* 2018;**13**:e0204151. doi:10.1371/journal.pone.0204151

42 Giel KE, Teufel M, Junne F, *et al.* Food-Related Impulsivity in Obesity and Binge Eating Disorder-A Systematic Update of the Evidence. *Nutrients* 2017;**9**. doi:10.3390/nu9111170

43 Kakoschke N, Kemps E, Tiggemann M. Approach bias modification training and consumption: A review of the literature. *Addict Behav* 2017;**64**:21–8. doi:10.1016/j.addbeh.2016.08.007

44 Eberl C, Wiers RW, Pawelczack S, *et al.* Implementation of approach bias re-training in alcoholism-how many sessions are needed? *Alcohol Clin Exp Res* 2014;**38**:587–94. doi:10.1111/acer.12281

45 Wittekind CE, Feist A, Schneider BC, *et al.* The approach-avoidance task as an online intervention in cigarette smoking: a pilot study. *J Behav Ther Exp Psychiatry* 2015;**46**:115–20. doi:10.1016/j.jbtep.2014.08.006

46 Sherman BJ, Baker NL, Squeglia LM, *et al.* Approach bias modification for cannabis use disorder: A proof-of-principle study. *Journal of Substance Abuse Treatment* 2018;**87**:16–22. doi:10.1016/j.jsat.2018.01.012

47 Schumacher SE, Kemps E, Tiggemann M. Bias modification training can alter approach bias and chocolate consumption. *Appetite* 2016;**96**:219–24. doi:10.1016/j.appet.2015.09.014

48 Warschburger P, Gmeiner M, Morawietz M, *et al.* Evaluation of an approach-avoidance training intervention for children and adolescents with obesity: A randomized placebo-controlled prospective trial. *Eur Eat Disord Rev* 2018;**26**:472–82. doi:10.1002/erv.2607

49 Brockmeyer T, Hahn C, Reetz C, *et al.* Approach Bias Modification in Food Craving-A Proof-of-Concept Study. *Eur Eat Disord Rev* 2015;**23**:352–60. doi:10.1002/erv.2382

50 Brockmeyer T, Friederich H-C, Küppers C, *et al.* Approach bias modification training in bulimia nervosa and binge-eating disorder: A pilot randomized controlled trial. *Int J Eat Disord* Published Online First: 28 January 2019. doi:10.1002/eat.23024

51 Becker D, Jostmann NB, Wiers RW, *et al.* Approach avoidance training in the eating domain: testing the effectiveness across three single session studies. *Appetite* 2015;**85**:58–65. doi:10.1016/j.appet.2014.11.017

52 Lindgren KP, Wiers RW, Teachman BA, *et al.* Attempted Training of Alcohol Approach and Drinking Identity Associations in US Undergraduate Drinkers: Null Results from Two Studies. *PLoS ONE* 2015;**10**:e0134642. doi:10.1371/journal.pone.0134642

53 Becker D, Jostmann NB, Holland RW. Does approach bias modification really work in the eating domain? A commentary on Kakoschke et al. (2017). *Addict Behav* 2018;**77**:293–4. doi:10.1016/j.addbeh.2017.02.025

For peer review only

For peer review only - http://bmjopen.bmj.com/site/about/guidelines.xhtml

54 Brunoni AR, Moffa AH, Sampaio-Junior B, *et al.* Trial of Electrical Direct-Current Therapy versus Escitalopram for Depression. http://dx.doi.org/10.1056/NEJMoa1612999. 2017. doi:10.1056/NEJMoa1612999

55 Bose A, Shivakumar V, Agarwal SM, *et al.* Efficacy of fronto-temporal transcranial direct current stimulation for refractory auditory verbal hallucinations in schizophrenia: A randomized, double-blind, sham-controlled study. *Schizophrenia Research* 2018;**195**:475–80. doi:10.1016/j.schres.2017.08.047

56 Lupi M, Martinotti G, Santacroce R, *et al.* Transcranial Direct Current Stimulation in Substance Use Disorders: A Systematic Review of Scientific Literature. *J ECT* 2017;**33**:203–9. doi:10.1097/YCT.0000000000000401

57 Dalton B, Campbell IC, Schmidt U. Neuromodulation and neurofeedback treatments in eating disorders and obesity. *Curr Opin Psychiatry* 2017;**30**:458–73. doi:10.1097/YCO.0000000000000361

58 Kekic M, McClelland J, Campbell I, *et al.* The effects of prefrontal cortex transcranial direct current stimulation (tDCS) on food craving and temporal discounting in women with frequent food cravings. *Appetite* 2014;**78**:55–62. doi:10.1016/j.appet.2014.03.010

59 Burgess EE, Sylvester MD, Morse KE, *et al.* Effects of transcranial direct current stimulation (tDCS) on binge eating disorder. *Int J Eat Disord* 2016;**49**:930–6. doi:10.1002/eat.22554

60 Fregni F, Orsati F, Pedrosa W, *et al.* Transcranial direct current stimulation of the prefrontal cortex modulates the desire for specific foods. *Appetite* 2008;**51**:34–41. doi:10.1016/j.appet.2007.09.016

61 Goldman RL, Borckardt JJ, Frohman HA, *et al.* Prefrontal cortex transcranial direct current stimulation (tDCS) temporarily reduces food cravings and increases the self-reported ability to resist food in adults with frequent food craving. *Appetite* 2011;**56**:741–6. doi:10.1016/j.appet.2011.02.013

62 Val-Laillet D, Aarts E, Weber B, *et al.* Neuroimaging and neuromodulation approaches to study eating behavior and prevent and treat eating disorders and obesity. *Neuroimage Clin* 2015;**8**:1–31. doi:10.1016/j.nicl.2015.03.016

63 Heeren A, Baeken C, Vanderhasselt M-A, *et al.* Impact of Anodal and Cathodal Transcranial Direct Current Stimulation over the Left Dorsolateral Prefrontal Cortex during Attention Bias Modification: An Eye-Tracking Study. *PLoS ONE* 2015;**10**:e0124182. doi:10.1371/journal.pone.0124182

64 Fritsch B, Reis J, Martinowich K, *et al.* Direct current stimulation promotes BDNF-dependent synaptic plasticity: potential implications for motor learning. *Neuron* 2010;**66**:198–204. doi:10.1016/j.neuron.2010.03.035

65 Sedgmond J, Lawrence NS, Verbruggen F, *et al.* Prefrontal brain stimulation during food-related inhibition training: effects on food craving, food consumption and inhibitory control. *R Soc Open Sci* 2019;**6**:181186. doi:10.1098/rsos.181186

66 Antal A, Terney D, Poreisz C, *et al.* Towards unravelling task-related modulations of neuroplastic changes induced in the human motor cortex. *Eur J Neurosci* 2007;**26**:2687–91. doi:10.1111/j.1460-9568.2007.05896.x

For peer review only - http://bmjopen.bmj.com/site/about/guidelines.xhtml

67 Wiers RW, Gladwin TE, Hofmann W, *et al.* Cognitive Bias Modification and Cognitive Control Training in Addiction and Related Psychopathology: Mechanisms, Clinical Perspectives, and Ways Forward. *Clinical Psychological Science* 2013;**1**:192–212. doi:10.1177/2167702612466547

68 den Uyl TE, Gladwin TE, Rinck M, *et al.* A clinical trial with combined transcranial direct current stimulation and alcohol approach bias retraining. *Addict Biol* 2017;**22**:1632–40. doi:10.1111/adb.12463

69 Ray MK, Sylvester MD, Osborn L, *et al.* The critical role of cognitive-based trait differences in transcranial direct current stimulation (tDCS) suppression of food craving and eating in frank obesity. *Appetite* 2017;**116**:568–74. doi:10.1016/j.appet.2017.05.046

70 Gluck ME, Alonso-Alonso M, Piaggi P, *et al.* Noninvasive neuromodulation targeted to the lateral prefrontal cortex induces changes in energy intake and weight loss in obesity. *Obesity (Silver Spring)* 2015;**23**:2149–56. doi:10.1002/oby.21313

71 Jauch-Chara K, Kistenmacher A, Herzog N, *et al.* Repetitive electric brain stimulation reduces food intake in humans. *Am J Clin Nutr* 2014;**100**:1003–9. doi:10.3945/ajcn.113.075481

72 Eldridge SM, Chan CL, Campbell MJ, *et al.* CONSORT 2010 statement: extension to randomised pilot and feasibility trials. *BMJ* 2016;**355**:i5239. doi:10.1136/bmj.i5239

73 Chan A-W, Tetzlaff JM, Altman DG, *et al.* SPIRIT 2013 Statement: defining standard protocol items for clinical trials. *Rev Panam Salud Publica* 2015;**38**:506–14.

74 Physical status: the use and interpretation of anthropometry. Report of a WHO Expert Committee. *World Health Organ Tech Rep Ser* 1995;**854**:1–452.

75 Association AP. *Diagnostic and Statistical Manual of Mental Disorders (DSM-5®)*. American Psychiatric Pub 2013.

76 Brunoni AR, Nitsche MA, Bolognini N, *et al.* Clinical research with transcranial direct current stimulation (tDCS): challenges and future directions. *Brain Stimul* 2012;**5**:175–95. doi:10.1016/j.brs.2011.03.002

77 Moher D, Schulz KF, Altman DG. The CONSORT statement: revised recommendations for improving the quality of reports of parallel-group randomised trials. *Lancet* 2001;**357**:1191–4.

78 Schulz KF, Altman DG, Moher D, *et al.* CONSORT 2010 statement: updated guidelines for reporting parallel group randomized trials. *Ann Intern Med* 2010;**152**:726–32. doi:10.7326/0003-4819-152-11-201006010-00232

79 Lancaster GA, Dodd S, Williamson PR. Design and analysis of pilot studies: recommendations for good practice. *J Eval Clin Pract* 2004;**10**:307–12. doi:10.1111/j.2002.384.doc.x

80 Julious SA. Sample size of 12 per group rule of thumb for a pilot study. *Pharmaceutical Statistics* 2005;**4**:287–91. doi:10.1002/pst.185

81 Schmidt U, Oldershaw A, Jichi F, *et al.* Out-patient psychological therapies for adults with anorexia nervosa: randomised controlled trial. *Br J Psychiatry* 2012;**201**:392–9. doi:10.1192/bjp.bp.112.112078

For peer review only - http://bmjopen.bmj.com/site/about/guidelines.xhtml

82 Schmidt U, Renwick B, Lose A, *et al.* The MOSAIC study - comparison of the Maudsley Model of Treatment for Adults with Anorexia Nervosa (MANTRA) with Specialist Supportive Clinical Management (SSCM) in outpatients with anorexia nervosa or eating disorder not otherwise specified, anorexia nervosa type: study protocol for a randomized controlled trial. *Trials* 2013;**14**:160. doi:10.1186/1745-6215-14-160

83 Fairburn CG, Beglin S, editors. *Cognitive Behavior Therapy and Eating Disorders.* New York: Guildford Press 2008.

84 Deurenberg P, Weststrate JA, Seidell JC. Body mass index as a measure of body fatness: age- and sex-specific prediction formulas. *British Journal of Nutrition* 1991;**65**:105–14. doi:10.1079/BJN19910073

85 Rinck M, Becker ES. Approach and avoidance in fear of spiders. *J Behav Ther Exp Psychiatry* 2007;**38**:105–20. doi:10.1016/j.jbtep.2006.10.001

86 Blechert J, Meule A, Busch NA, *et al.* Food-pics: an image database for experimental research on eating and appetite. *Front Psychol* 2014;**5**:617. doi:10.3389/fpsyg.2014.00617

87 De Houwer J, Crombez G, Baeyens F, *et al.* On the generality of the affective Simon effect. *Cognition & Emotion* 2001;**15**:189–206. doi:10.1080/02699930125883

88 Steinglass J, Foerde K, Kostro K, *et al.* Restrictive food intake as a choice--a paradigm for study. *Int J Eat Disord* 2015;**48**:59–66. doi:10.1002/eat.22345

89 Foerde K, Gianini L, Wang Y, *et al.* Assessment of test-retest reliability of a food choice task among healthy individuals. *Appetite* 2018;**123**:352–6. doi:10.1016/j.appet.2018.01.010

90 Hare TA, Camerer CF, Rangel A. Self-control in decision-making involves modulation of the vmPFC valuation system. *Science* 2009;**324**:646–8. doi:10.1126/science.1168450

91 Cepeda-Benito A, Gleaves DH, Williams TL, *et al.* The development and validation of the state and trait food-cravings questionnaires. *Behavior Therapy* 2000;**31**:151–73. doi:10.1016/S0005-7894(00)80009-X

92 Meule A, Lutz A, Vögele C, *et al.* Food cravings discriminate differentially between successful and unsuccessful dieters and non-dieters. Validation of the Food Cravings Questionnaires in German. *Appetite* 2012;**58**:88–97. doi:10.1016/j.appet.2011.09.010

93 Kekic M, McClelland J, Bartholdy S, *et al.* Single-Session Transcranial Direct Current Stimulation Temporarily Improves Symptoms, Mood, and Self-Regulatory Control in Bulimia Nervosa: A Randomised Controlled Trial. *PLoS ONE* 2017;**12**:e0167606. doi:10.1371/journal.pone.0167606

94 Meule A, Hermann T, Kübler A. A short version of the Food Cravings Questionnaire-Trait: the FCQ-T-reduced. *Front Psychol* 2014;**5**:190. doi:10.3389/fpsyg.2014.00190

95 Lowe MR, Butryn ML, Didie ER, *et al.* The Power of Food Scale. A new measure of the psychological influence of the food environment. *Appetite* 2009;**53**:114–8. doi:10.1016/j.appet.2009.05.016

96 Gearhardt AN, Corbin WR, Brownell KD. Development of the Yale Food Addiction Scale Version 2.0. *Psychol Addict Behav* 2016;**30**:113–21. doi:10.1037/adb0000136

For peer review only

97 Gearhardt AN, White MA, Masheb RM, *et al.* An examination of food addiction in a racially diverse sample of obese patients with binge eating disorder in primary care settings. *Compr Psychiatry* 2013;**54**:500–5. doi:10.1016/j.comppsych.2012.12.009

98 Steward T, Mestre-Bach G, Vintró-Alcaraz C, *et al.* Food addiction and impaired executive functions in women with obesity. *European Eating Disorders Review* 2018;**26**:574–84. doi:10.1002/erv.2636

99 Meule A, Heckel D, Kübler A. Factor Structure and Item Analysis of the Yale Food Addiction Scale in Obese Candidates for Bariatric Surgery. *European Eating Disorders Review* 2012;**20**:419–22. doi:10.1002/erv.2189

100 Clark SM, Saules KK. Validation of the Yale Food Addiction Scale among a weight-loss surgery population. *Eating Behaviors* 2013;**14**:216–9. doi:10.1016/j.eatbeh.2013.01.002

101 Carter JC, Van Wijk M, Rowsell M. Symptoms of 'food addiction' in binge eating disorder using the Yale Food Addiction Scale version 2.0. *Appetite* 2019;**133**:362–9. doi:10.1016/j.appet.2018.11.032

102 Robinson E, Haynes A, Hardman CA, *et al.* The bogus taste test: Validity as a measure of laboratory food intake. *Appetite* 2017;**116**:223–31. doi:10.1016/j.appet.2017.05.002

103 Odum AL, Baumann AAL, Rimington DD. Discounting of delayed hypothetical money and food: effects of amount. *Behav Processes* 2006;**73**:278–84. doi:10.1016/j.beproc.2006.06.008

104 Teslovich T, Freidl EK, Kostro K, *et al.* Probing behavioral responses to food: development of a food-specific go/no-go task. *Psychiatry Res* 2014;**219**:166–70. doi:10.1016/j.psychres.2014.04.053

105 Verbruggen F, Logan GD. Response inhibition in the stop-signal paradigm. *Trends Cogn Sci (Regul Ed)* 2008;**12**:418–24. doi:10.1016/j.tics.2008.07.005

106 Manasse SM, Goldstein SP, Wyckoff E, *et al.* Slowing down and taking a second look: Inhibitory deficits associated with binge eating are not food-specific. *Appetite* 2016;**96**:555–9. doi:10.1016/j.appet.2015.10.025

107 Gross JJ, John OP. Individual differences in two emotion regulation processes: implications for affect, relationships, and well-being. *J Pers Soc Psychol* 2003;**85**:348–62.

108 Watson D, Clark LA, Tellegen A. Development and validation of brief measures of positive and negative affect: the PANAS scales. *J Pers Soc Psychol* 1988;**54**:1063–70.

109 Lovibond SH, Lovibond PF. *Manual for the Depression Anxiety Stress Scales (2nd. ed.)*. Sydney Psychology Foundation 1995.

110 Jones EB, Sharpe L. Cognitive bias modification: A review of meta-analyses. *J Affect Disord* 2017;**223**:175–83. doi:10.1016/j.jad.2017.07.034

111 Song S, Zilverstand A, Gui W, *et al.* Effects of single-session versus multi-session non-invasive brain stimulation on craving and consumption in individuals with drug addiction, eating disorders or obesity: A meta-analysis. *Brain Stimul* Published Online First: 27 December 2018. doi:10.1016/j.brs.2018.12.975

For peer review only - http://bmjopen.bmj.com/site/about/guidelines.xhtml

112 Neumann R, Strack F. Approach and avoidance: the influence of proprioceptive and exteroceptive cues on encoding of affective information. *J Pers Soc Psychol* 2000;**79**:39–48.

113 Vrijsen JN, Fischer VS, Müller BW, *et al.* Cognitive bias modification as an add-on treatment in clinical depression: Results from a placebo-controlled, single-blinded randomized control trial. *J Affect Disord* 2018;**238**:342–50. doi:10.1016/j.jad.2018.06.025

114 Becker ES, Ferentzi H, Ferrari G, *et al.* Always Approach the Bright Side of Life: A General Positivity Training Reduces Stress Reactions in Vulnerable Individuals. *Cognit Ther Res* 2016;**40**:57–71. doi:10.1007/s10608-015-9716-2

115 Brockmeyer T, Schmidt U, Friederich H-C. The ABBA study - approach bias modification in bulimia nervosa and binge eating disorder: study protocol for a randomised controlled trial. *Trials* 2016;**17**:466. doi:10.1186/s13063-016-1596-6

116 Poreisz C, Boros K, Antal A, *et al.* Safety aspects of transcranial direct current stimulation concerning healthy subjects and patients. *Brain Research Bulletin* 2007;**72**:208–14. doi:10.1016/j.brainresbull.2007.01.004

117 Hutson PH, Balodis IM, Potenza MN. Binge-eating disorder: Clinical and therapeutic advances. *Pharmacol Ther* 2018;**182**:15–27. doi:10.1016/j.pharmthera.2017.08.002

118 Vroling MS, Wiersma FE, Lammers MW, *et al.* Predicting Dropout from Intensive Outpatient Cognitive Behavioural Therapy for Binge Eating Disorder Using Pre-treatment Characteristics: A Naturalistic Study. *Eur Eat Disord Rev* 2016;**24**:494–502. doi:10.1002/erv.2474

119 Marinilli Pinto A, Guarda AS, Heinberg LJ, *et al.* Development of the eating disorder recovery self-efficacy questionnaire. *Int J Eat Disord* 2006;**39**:376–84. doi:10.1002/eat.20256

120 Patton JH, Stanford MS, Barratt ES. Factor structure of the Barratt impulsiveness scale. *J Clin Psychol* 1995;**51**:768–74.

121 Bohn K, Fairburn CG. *The Clinical Impairment Assessment Questionnaire (CIA 3.0). In Cognitive Behavior Therapy and Eating Disorders.* New York: Guildford Press 2008.

122 Hoerger M, Quirk SW, Weed NC. Development and validation of the Delaying Gratification Inventory. *Psychol Assess* 2011;**23**:725–38. doi:10.1037/a0023286

For peer review only

For peer review only - http://bmjopen.bmj.com/site/about/guidelines.xhtml

Figure 1 Study procedure. The 3 assessment time points are baseline, post-assessment and follow-up. IG1 is intervention group 1; IG2 is intervention group 2, CG is the wait list control group.

Table 1 Study schedule of measurement and testing time points

	**Screening of potential participants**	**Baseline assessment (all participants)**	**Training: 6 sessions of ABM + real tDCS**	**Training: 6 sessions of ABM + sham tDCS**	**Post-assessment (all participants**	**Follow-up (all participants)**
**Approximate time since baseline**	-------	-------	**0-3 weeks**	**0-3 weeks**	**3 weeks**	**7 weeks**
Informed consent		X				
EDDS, SCID-I	X					
TDCS safety screening	X					
Demographic information		X				
EDE-Q[83]		X			X	X
Inhibitory control tasks; Go/No-Go task[104], SST[106]		X			X	X
Delay Discounting Task with Money and Food[103]		X			X	X
Food related tasks; Food Choice Task[88], FCT, Bogus Taste Test[102]		X			X	
Approach bias assessment tasks; F-AAT[85], SRC[87]		X			X	X
Questionnaires & scales (incl. at home); EDRSQ[119], FCQ-T-r[94], PFS[95], YFAS 2.0[96], ERQ[107], PANAS[108], BIS-11[120], CIA[121], DGI[122], DASS-21[109]		X			X	X
Pre-[tDCS + ABM] measures: -Multiple VAS, blood pressure, pulse			X	X		

For peer review only - http://bmjopen.bmj.com/site/about/guidelines.xhtml

Real or sham tDCS to dLPFC	X	X	
Approach Bias Modification training	X	X	
Post-[tDCS + ABM] measures:<br>-Multiple VAS, blood pressure, pulse	X	X	
Tolerance, discomfort and side effects	X	X	
Acceptability questionnaire			X
Blinding assessment questionnaire			X

BIS-11; Barrett Impulsiveness Scale, CIA; Clinical Impairment Assessment, DASS-21; Depression, Anxiety and Stress Scale, DGI; Delayed Gratification Inventory, EDDS; Eating Disorder Diagnostic Screen, EDE-Q; Eating Disorder Examination, EDRSQ; Eating Disorder Recovery Self Efficacy Questionnaire, ERQ; Emotion Regulation Questionnaire, FCQ-T-r; Food Cravings Questionnaire Trait Version – reduced, PANAS; Positive and Negative Affect Schedule, PFS; The 21-item Power of Food Scale, SCID-I; Structured Clinical Interview for Diagnostic and Statistical Manual (DSM) Axis I Disorders, SRC; Stimulus Response Compatibility Task, SST; Stop Signal Task, YFAS 2.0; The Yale Food Addiction Scale Version 2.0, .

For peer review only

For peer review only - http://bmjopen.bmj.com/site/about/guidelines.xhtml

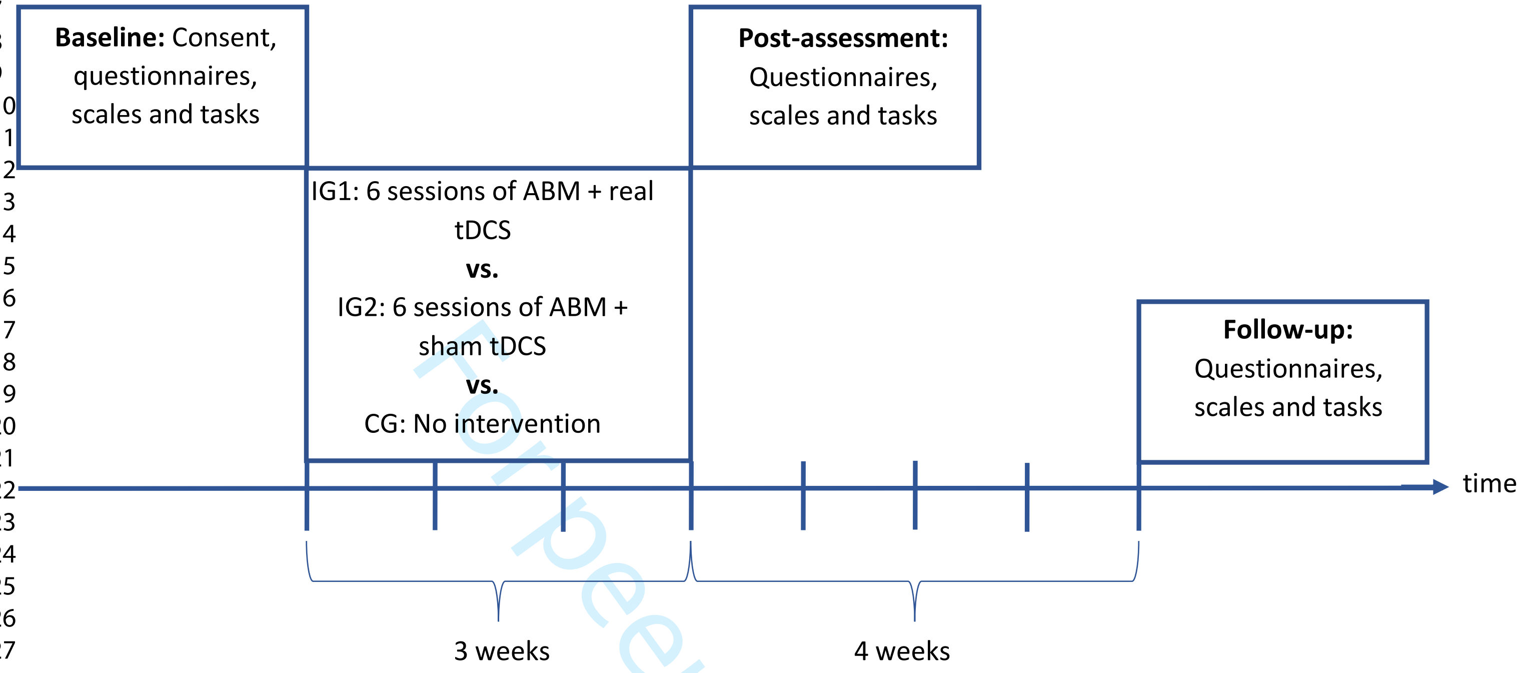

For peer review only

Supplementary File

Appendix A: ICARUS study task, questionnaire, measure and scale information

(1) *Approach bias assessment tasks*

(i) Food Approach-Avoidance Task (F-AAT) (Rinck & Becker, 2007): The F-AAT is a computerised task that measures approach and avoidance behaviour by means of joystick movements in response to food and neutral stimuli presented on a computer screen. This task will be used to assess approach bias towards visual cues of high-calorie food (expected target cognitive mechanism of intervention). Images of palatable edible foods (i.e. chocolate, pizza) and non-edible objects (i.e. sponges, stapler) are used as in previously (Brockmeyer et al., 2019). The assessment version of the AAT is identical to the treatment version except that the required response is unrelated to the picture content (i.e. food and neutral stimuli are presented equally often in round and rectangular, i.e. push and pull, format). Format movement assignments are counterbalanced among participants (i.e., half push round pictures and half push rectangular pictures). When the joystick is pulled, the picture grows bigger, and diminishes in size when the joystick is pushed. Zooming-in and zooming-out via joystick movements enacts motions of approaching and avoiding respectively and thus combines the proprioceptive (arm movement) and exteroceptive (zooming feature) cues of approach and avoidance behaviour (Neumann & Strack, 2000; Brockmeyer et al., 2019). The assessment version of the AAT consists of 80 trials (40 food item pictures and 40 non-food item pictures). To evaluate approach bias towards food, a compatibility score is calculated by subtracting the median reaction times (RTs) of compatible trials (i.e., RT pull food + RT push nonfood) from median RTs of incompatible trials (i.e., RT push food + RT pull nonfood) (Brockmeyer et al., 2019; Becker et al., 2016; Vrijsen et al., 2018). A positive value indicates a food-specific approach bias (i.e. the participant is faster at pulling than pushing food pictures, relative to the approach bias towards non-food), whereas a negative value indicates an avoidance bias. This task will be performed using Inquisit 5 (Millisecond Software).

(ii) The Stimulus Response Compatibility Task (SRC) (De Houwer, Crombez, Baeyens, & Hermans, 2001): In this task, participants respond to images on a computer screen by pressing keys on the keyboard. Pictures are presented in the centre of the screen with a manikin (12 mm high) positioned 33 mm above or below the picture. Participants are required to categorise the presented pictures by making an approach response (pressing the *up* or *down* key to move the manikin toward the picture) or an avoidance response (pressing the *up* or *down* key to move the manikin away from the picture). After making a correct response, an animation is shown of the manikin walking toward the picture (approach) or away from the picture (avoidance) for 1,000 ms. After making an incorrect response, a red cross appears on the screen for 500 ms, after which the next trial starts. Fourteen food and fourteen non-food pictures will be used from the food-pics database (Blechert, Meule, Busch & Ohla, 2014). The experiment comprises 8 practice trials and 56 experimental trials. Bias scores will be calculated by subtracting the mean of approach food/avoid non-food trials from the mean of avoid food/approach non-food trials. A positive score indicates a food-related approach bias, with higher scores indicative of stronger biases. This task will be performed using Inquisit 5 (Millisecond Software).

For peer review only - http://bmjopen.bmj.com/site/about/guidelines.xhtml

(2) Food Choice Task

The Food Choice Task (Hare, Camerer & Rangel., 2009) adapted for eating disorders (Steinglass et al., 2015) is a computer-based paradigm that measures responses to images of foods to assess food attitudes and characteristics of eating behaviour. Food stimuli is used to investigate how individuals make decisions about what to eat, and measures decision-making around food by directly probing personal preferences. There are no learning requirements and individualised assessments of food along two dimensions (healthiness and tastiness) allow the tasks to be used in diverse populations with differing valuations of food. The task consists of three phases. In each phase participants are presented with 43 images of food items. The food items represent an array of dietary options (Steinglass et al., 2015). Twenty-five food items are low fat (<30% calories from fat) and 18 are high fat (>30% calories from fat), as determined by Foerde et al. (2018). In the Health phase, participants rate the healthiness of each food item on a 5-point scale (*1 = "Unhealthy", 5 = "Healthy"*). In the Taste phase, participants rate the tastiness of each food item in a similar fashion, (*1 = "Bad", 5 = "Good"*). In the Choice phase, in each trial participants choose between the presented food item and a "Neutral" reference food item (rated as 3 in both Health and Taste phases). If no item is rated 3 on both scales, an item rated 3 on Health and greater than 3 on the Taste scale is selected as a reference food. Good test-retest reliability of the FCT suggests that it is suitable for measuring food-based decision-making in studies with multiple assessment points (Foerde et al., 2018).

(3) *State food craving - Food craving after cue exposure task*

The Food Challenge Task (FCT) (Kekic et al., 2017; Meule et al., 2018) will be used to examine cue-induced food craving. In this task, participants rate their state food craving using the Food Cravings Questionnaire State Version (Cepeda-Benito et al., 2000; Meule et al., 2012) before and after being presented with a video on a computer screen of foods shown to be highly appetitising and to elevate hunger levels (Kekic et al., 2017). The questionnaire consists of 15 items that measure the strength of food cravings (i.e. *I would feel more alert if I could satisfy my craving*). Participants are asked to indicate how much they agree with each statement 'at this very moment' using a five-point scale (from 1 'strongly disagree' to 5 'strongly agree'). There are five craving subscales; intense desire to eat, anticipation of relief from negative states, physiological craving, preoccupation with food or lack of control over eating and anticipation of positive reinforcement. Scores can be calculated for specific subscales or a total score can be calculated (ranging from 15 to 75). This questionnaires will be completed using Inquisit 5 (Millisecond Software), and the video shown using QuickTime player.

(4) *Trait food craving*

The Yale Food Addiction Scale Version 2.0 (YFAS 2.0; Gearhardt, Corbin, & Brownell, 2016) is the most commonly used instrument to assess food-related addictive behaviours (Steward et al., 2018). This self-report questionnaire consists of 35 items scored on an 8-point Likert scale (from 0 = never to 7 = every day) and is adapted to assess addictive eating behaviours based on DSM-5 substance-related and addictive disorders criteria (APA, 2013). It refers specifically to consumption of foods high in fat, sugar, salt or refined carbohydrates. It

For peer review only - http://bmjopen.bmj.com/site/about/guidelines.xhtml

includes items that assess specific criteria, such as diminished control over consumption, a persistent desire or repeated unsuccessful attempts to quit, withdrawal, and clinically significant impairment (i.e*. 'I kept eating in the same way even though my eating caused emotional problems')*. The YFAS includes two scoring options: 1) a "symptom count" ranging from 0 to 7 that reflects the number of addiction-like criteria endorsed and 2) a categorical scoring option that classifies respondents as having either no, mild, moderate or severe 'food addiction'. The YFAS has received psychometric support in binge eating populations (Gearhardt, White et al., 2013; Carter, Van Wijk & Rowsell, 2019), and obese bariatric surgery patients (Clark & Saules, 2013; Meule, Heckel, & Kübler, 2012). The YFAS 2.0 was developed to maintain consistency with the current diagnostic understanding of addiction and to improve the psychometric properties of the original YFAS. Exceeding the food addiction threshold was more strongly associated with obesity for the YFAS 2.0 than the original YFAS. The YFAS 2.0 has demonstrated good internal consistency (Carter, Van Wijk & Rowsell, 2019), as well as convergent, discriminant and incremental validity (Gearhardt, Corbin & Brownell, 2016).

(5) *Food intake in a Bogus Taste Test*

Actual food consumption will be measured by means of a Bogus Taste Test (Robinson et al., 2017); a food consumption test presented under the guise of a taste test. Participants will be instructed to rate 3 bowls of highly palatable high-calorie food items (chocolate, sweets, crisps) in terms of their visual attractiveness, smell, and taste on a paper form. The researcher will inform the participant that she/he will leave the room for 10 minutes and during this time they can complete their ratings and are free to eat as much of the offered items as they like. A small bin with lid will be provided and participants will be instructed to consume as much food as they need or want, and to discard the remainder of the food items in the bin before 10 minutes are over. After the participant has left, the discarded food items will be recovered from the bin, sorted, and placed in their original bowls. Consumption will be determined by weighing the bowls both before and after the "taste test" and the difference in weight from pre-to post-assessment will be converted into calories and used as a measure of food intake.

(6) *Delay discounting*

Delay Discounting Task with Money and Food (Odum, Baumann, & Rimington, 2006): Participants indicate their preferences in a series of choices for two hypothetical outcome types: immediate versus delayed food and immediate versus delayed money. Participants make choices involving either relatively small maximum amounts of food (10 dollars worth) and money (10 dollars) or for relatively large maximum amounts of food (100 dollars worth) and money (100 dollars). Performance on this task can be used to study self-regulation, delayed gratification and valuation of reward. This task will be performed using Inquisit 5 (Millisecond Software).

(7) *Inhibitory control*

(i) Go/No-Go task: The cued go/no go task is a useful measure of impulse control in clinical populations. This task is a classic test of executive function, requiring effortful response inhibition. The food specific version of the cued go no-go task (Teslovich et al., 2014) measures impulsivity and response inhibition with respect to appetising food and nonfood items (i.e. toys), via assessing the ability to inhibit instigated, prepotent responses. The task

For peer review only - http://bmjopen.bmj.com/site/about/guidelines.xhtml

manipulates response prepotency by presenting a preliminary go or no-go cue before the actual go or no-go target is displayed. The cues provide information concerning the probability that a go or no-go target will be presented. The cue-target relationship is manipulated so that the cues have a high probability of correctly signaling a go or no-go target (valid cues), and a low probability of incorrectly signaling a target (invalid cues). Valid cues tend to facilitate response inhibition and speed response execution, whereas invalid cue cues tend to impair response inhibition and slow response execution. The set of stimuli consists of 30 colour images of common high- (8) and low-calorie (7) foods and common toys (15). The outcome variables include: (1) overall reaction time (RT) in milliseconds during correct "go" trials, (2) rate of omission errors (missed "go" trials), and (3) false alarm rate (rate at which participants erroneously press to a no-go stimulus). This task will be performed using Inquisit 5 (Millisecond Software).

(i) Stop Signal Task (SST): This is a task measuring inhibitory control. Participants are required to engage in a computer task but withhold their response in the presence of a stop signal. This SST includes food-specific and neutral non-food stimuli in the same task (adaptation of Manasse et al., 2016). This allows for isolation of any unique food specific inhibitory control deficits from general difficulties inhibiting responses. The outcome measure is the stop signal reaction time (SSRT). The SSRT is calculated for each set of stimuli (i.e., SSRT stimulus type) for each subject by subtracting the average stop signal delay from the average reaction time on "go" trials (Verbruggen & Logan, 2008). The recording accuracy of reaction time and stop signal delay measurement is in milliseconds. A smaller SSRT is indicative of greater inhibitory control and a larger SSRT reflects weaker/impaired inhibitory control. This task will be performed using Inquisit 5 (Millisecond Software).

(8) *Mood and emotion regulation*
The Emotion Regulation Questionnaire (ERQ) (Gross & John, 2003): A 10-item scale designed to measure respondents' tendency to regulate their emotions in two ways: (1) cognitive reappraisal and (2) expressive suppression. Respondents answer each item on a 7-point Likert-type scale ranging from 1 (strongly disagree) to 7 (strongly agree). This will be administered at pre-assessment, post-assessment and follow-up.

Positive and Negative Affect Schedule (PANAS) (Watson, Clarke & Tellegen, 1988): The PANAS consists of two 10-item self-report scales which measure positive and negative affect. On a Likert scale ranging from 0 (very slightly or not at all) to 4 (extremely), participants rate the extent to which they have experienced each of the 20 descriptors within a particular time frame ("right now" in the current study). Two scores are generated: positive (PANAS-positive) and negative (PANAS- negative) affect. This will be administered at pre-assessment, post-assessment and follow-up.

Depression, Anxiety and Stress Scale (21-item version; DASS-21) (Lovibond & Lovibond, 1995): This is a 21 item self-report questionnaire which aims to evaluate mood, anxiety and stress levels over the previous week. The DASS-21 will be administered at pre-assessment, post-assessment and follow-up.

(9) *Within session measures (immediately after each [ABM+real/sham tDCS] treatment session)*

For peer review only

(i) Paper-based Visual Analogue Scales (VAS) assessing current hunger, feeling of fullness, urge to eat, urge to binge eat, feeling low, level of tension, level of stress, level of anxiety. These scales consist of a 10cm line. Participants are requested to indicate on this line a degree or level of a specific emotion or behavioural urge. There are indications of what range (e.g. from 'not at all' to 'extremely').

(ii) Tolerance, discomfort and side effects: An evaluation of discomfort with the training session (tDCS and ABM aspects separately) will be completed with another paper-based 10cm VAS (rated from none to extreme discomfort). Participants will be asked to report any side effects in an open ended question.

Appendix B: Information sheet for all participants

PARTICIPANT INFORMATION SHEET

ICARUS: An Investigation of Approach Bias Modification Training (ABM) and Transcranial Direct Current Stimulation (tDCS) in Binge Eating Disorder

KING'S College LONDON

IRAS Project ID: 244170
Research Ethics Committee reference number: 18/NW/0648

We would like to invite you to participate in this postdoctoral research project which is being conducted by a PhD student for research and educational purposes. You should only participate if you want to; choosing not to take part will not disadvantage you in any way. Before you decide whether you want to take part, it is important for you to understand why the research is being done and what your participation will involve. Please take time to read the following information carefully and discuss it with others if you wish. Part 1 tells you the purpose of this study and what will happen to you if you take part. Part 2 gives you more detailed information about the conduct of the study. Please ask us if there is anything that is not clear or if you would like more information.

PART ONE

What is the purpose of the study?
Psychological therapy as a main treatment for Binge Eating Disorder (BED) may not be effective for many people and may not be readily accessible in some areas. Medical treatments for BED can have side effects and often do not remain effective in the long-term. Therefore, there is an ongoing need for the development of new treatments.

Computerised approach bias modification training (ABM) is a specific form of cognitive bias modification (CBM) that has been used to successfully treat mental disorders such as anxiety, depression, and addictive disorders. This technique involves several sessions of computerised training, a procedure which has shown to be effective in reducing the severity of some eating disorder symptoms in people with BED and Bulimia Nervosa (BN). ABM works as such; automatic approach and avoidance tendencies towards food-related cues are modified by repeated training of arm movements in front of a computer

For peer review only - http://bmjopen.bmj.com/site/about/guidelines.xhtml

screen. ABM has shown to reduce approach tendencies and attention towards food cues in a subclinical sample of eating disorders involving binge eating, but its efficacy on these features in people with full-syndrome eating disorders remains unclear. Further research is needed to examine if ABM is effective in reducing the frequency of binge eating episodes in people with BED.

Transcranial direct current stimulation (tDCS) is a non-invasive technique that is capable of stimulating specific brain areas. Research shows that the frontal areas of the brain play a role in the development and maintenance of eating disorders, including BED. Stimulating these brain areas to alter their functioning is therefore believed to have the potential to reduce eating disorder symptoms. This involves the delivery of a low electrical current via small electrodes placed on the scalp. This procedure is widely used in research and is being applied in clinical settings. Recent research using tDCS on people with BED has suggested that it may be helpful in reducing immediate food intake and cravings, and may decrease the frequency of a desire to binge eat at home after the treatment.

Combining ABM and tDCS

Previous studies suggest that these two techniques potentially help people better regulate their behaviours through similar mechanisms in the brain. Delivering both treatments together at the same time may have a stronger effect on reducing eating disorder symptoms in people with BED than either of the treatments alone. This will be the first time that this specific combination of interventions is conducted on people with an eating disorder.

In the present study, we aim to investigate combined ABM and tDCS as a treatment for BED by comparing the effect of 6 sessions of (ABM + real tDCS) vs. (ABM + placebo tDCS) across a 3-week period in adult men and women with BED. We will also compare these two groups against a control group. Participants will be allocated by chance to either one of two intervention groups, or to the control group. Participants in the intervention groups will receive 6 sessions of ABM delivered simultaneously with either real tDCS or a placebo version of tDCS. Participants assigned to the control group will not receive any intervention. We will measure eating disorder symptoms and other outcomes in all participants at baseline, post-treatment, and at the 4-week follow-up to assess outcomes of each study group. In particular, we are interested in changes in the frequency of binge eating and craving, and thought processes and emotions related to food and eating. We will also ask participants about their experience of this treatment. Participants assigned to the control condition will be offered 6 sessions of (ABM + real tDCS) after they have completed their involvement in the study.

Why have I been invited?

You are invited to participate if you are a male or female aged between 18 and 70 who has a current diagnosis of binge eating disorder (BED). We will be recruiting 66 participants in total.

Do I have to take part?

You do not have to take part in this experiment; it is your choice. If you decide to take part, you will be asked to sign three identical consent forms. You will be free to

For peer review only - http://bmjopen.bmj.com/site/about/guidelines.xhtml

withdraw from the study at any time without giving a reason. Whether you decide to take part or not will in no way influence your care or the timing of your treatment.

What will happen to me if I take part and what will I have to do?
If you decide you want to participate you will firstly be asked to engage in a telephone conversation with the researcher (lasting approximately 20 minutes) to confirm that you are eligible to take part. If you are, you will be invited to the Institute of Psychiatry, Psychology and Neuroscience (Kings College London, Denmark Hill Campus) for a baseline assessment session on a day that is convenient for both you and the researcher, in either the morning or the afternoon. On this day, the researcher will discuss the study with you in person, answer your questions, and if you are happy to take part, we will ask you to sign three copies of a consent form: one for you to keep, one for us to keep, and one that will be sent to your general practitioner (GP).

This baseline assessment visit is longer that the treatment visits and is comprised of an in-person visit and online questionnaires to complete at home. During the visit, you will give informed study consent, complete two questionnaires, neuropsychological tasks (brain puzzles), and a food task for which you will be asked to rate different foods. This visit also involves an assessment version of the approach bias modification (ABM) training programme, which is a computer based-task involving pushing and pulling a joystick in response to shapes appearing on the computer screen. Weight and height will be measured at each assessment, and participants may choose not to see the figures recorded. Assessment visits will last 65-75 minutes. Within 36 hours after the in-person visit, you will be emailed a link to a series of questionnaires (assessing mood and eating disorder-related thoughts and habits) which will take 30-45 minutes to complete. There is also the option to complete these assessment questionnaires in hardcopy paper format.

You will then be then randomised and informed of your randomly assigned study condition within a week. If you are assigned to one of the two intervention conditions, you will be asked to attend 6 sessions where you will perform the ABM task while receiving either real or placebo tDCS, in addition to completing the post-treatment and follow-up assessments. If you are assigned to the control group, you will not receive any intervention, and will be asked to attend the post-treatment and follow-up assessments.

All 6 intervention sessions will be identical and last approximately 40–50 minutes. There is no need for any special preparation before the visits. Before and after each intervention session, you will complete some scales related to aspects of your mood and level of hunger. Your blood pressure and pulse will also be measured before and after each intervention session to monitor your wellbeing.
During the (ABM + real/placebo tDCS) session you will sit on a comfortable chair facing a computer screen, with a joystick on a table in front of you. You will wear a plastic headband to keep the two tDCS electrodes in place (as shown in the diagram below). The electrodes will be placed in small sponges soaked in a salt water solution, so they might feel a bit wet against your head. The researcher will turn the machine on which will deliver the currents. Depending on your assigned study condition, you will receive real or placebo brain stimulation. The placebo session will be the same as the real session, but the tDCS machine won't deliver any electrical current. Most people can't tell the difference between real and placebo tDCS sessions.

For peer review only - http://bmjopen.bmj.com/site/about/guidelines.xhtml

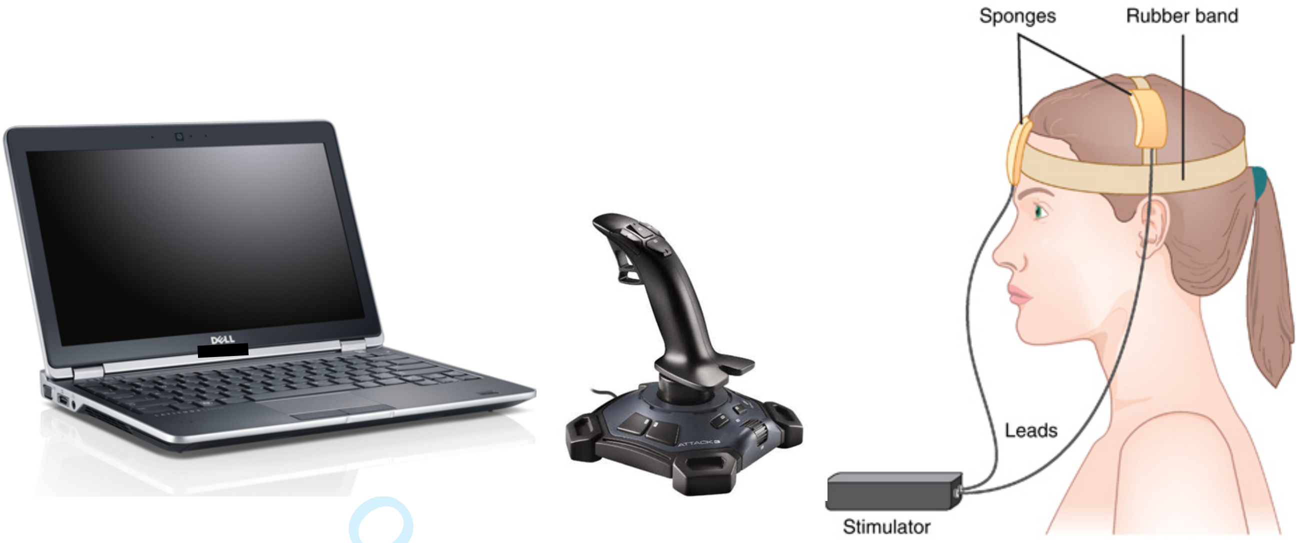

For peer review only

The tDCS will begin a few minutes prior to the start of the computer training programme, to allow participants to become used to the sensation before starting the ABM task on the computer. The training version of the ABM programme is a computer based-task involving pushing and pulling a joystick in response to shapes appearing on the computer screen. The tDCS will also continue for a few minutes after the ABM task has ended. You will then be asked to rate any discomfort experienced during the session due the tDCS.

You will be asked to return to the Institute for 5 more identical intervention sessions within 3 weeks, leaving a gap of at least 24 hours between each session. The post-assessment will be conducted immediately after the 6th intervention session. This in-person visit comprising of the final intervention session plus the post-assessment will therefore last 90-120 minutes, with the online questionnaires to be completed within 36 hours after this visit (45 minutes). The follow-up assessment 4 weeks later will be similar to the baseline assessment. This final visit will therefore take 75 minutes, with the online questionnaires taking 45 minutes to complete at home afterwards.

20 participants (10 participants from each intervention condition) will be invited to provide feedback on their experiences of study participation, initial expectations of the intervention, perceived strengths and weaknesses, and suggestions for improvements in an interview with the researcher. Declining this invitation to interview does not affect study payment.

6 months after the baseline assessment, participants may be contacted by phone by a member of the King's College London Eating Disorders Unit for a brief phone call to evaluate the presence of eating disorder symptoms. This 6 month check-in will allow any long-term therapeutic effects of the study treatment intervention to be evaluated. Participation in this phone call will not affect participant payment, which will have been administered earlier after the 1-month follow up assessment.

Expenses and payments

For peer review only - http://bmjopen.bmj.com/site/about/guidelines.xhtml

Upon completion of the study, all participants will be paid a maximum of £60 for completing each of the three assessment sessions (comprised of an in-person visit and at-home questionnaires); the baseline assessment, post-treatment assessment and follow-up assessment (£20 each). This payment should be declared for tax and/or benefit purposes. If you are assigned to an intervention condition involving 6 sessions of brain stimulation and computer training, you may also be compensated up to £10 per day for your travel expenses on these intervention session days.

What is expected from you as a participant?
We would expect you to complete all assessment sessions (pre-, post-treatment, follow-up), and if you are randomised to an intervention group, to attend all 6 (ABM + real/placebo tDCS) sessions as scheduled. We ask you to inform us immediately if for any reason you suddenly find yourself unable to attend a scheduled session.

Please let us know of any health problem that has developed, or any new diagnosis made since you enrolled for the study. Further, we would ask you to let us know of any new medication or change in medication whilst you are taking part in the study.

What are the possible disadvantages and risks of taking part? What are the side effects?
Combined brain stimulation and cognitive training sessions are time-consuming and may cause fatigue from concentrating on the task.

TDCS has been shown to be safe when used correctly in a clinical setting. However, you may find the procedure slightly uncomfortable. This is because a number of sensations can occur beneath the electrodes during stimulation including tingling, pain, itching, and burning. Not everyone feels these sensations or finds them uncomfortable, but if you do, remember you are free to stop the study at any point without giving an explanation. In some rarer cases, tDCS has been known to cause a headache, but this can be treated with mild painkillers (e.g. paracetamol). No side effects of ABM are known. We will assess any discomfort you may experience during intervention sessions throughout your involvement in the trial.

What are the possible benefits of taking part?
Unfortunately, there are no direct benefits to taking part in this study, but the information we get may help us to improve the treatment of BED in the future.

What happens when the research study stops?
When the research study stops, no further ABM + tDCS sessions will be available to those who have received 6 sessions. Participants in the control group will have the option of receiving 6 sessions of [ABM + real tDCS] once they have completed the waiting period and follow-up assessment.
Participants assigned to one of the intervention groups can request to be informed if they had received ABM combined with real or placebo tDCS, once they have completed the follow-up assessment.

What if there is a problem?
Any complaint about the way you have been dealt with during the study or any possible harm you might suffer will be addressed. Detailed information on this is given in Part 2.

For peer review only - http://bmjopen.bmj.com/site/about/guidelines.xhtml

Will my taking part in the study be kept confidential?
Yes. We will follow ethical and legal practice and all information about you will be handled in confidence. The details are included in Part 2.

If the information in Part 1 has interested you and you are considering participation, please read the additional information in Part 2 before making a decision.

PART TWO

What if relevant and new information becomes available?
Sometimes we get new information about the treatment being studied. This is not expected to occur given the short time frame of participation (6 sessions across 3 weeks); however, if any new and relevant information becomes available during this time we will inform you immediately. You can then decide whether you wish to continue in the study.

What will happen if I don't want to carry on with the study?
Your participation is voluntary and you do not have to take part in the study. You can change your mind at any point and terminate your participation without giving a reason to the researcher. You are free to withdraw from this study at any time without consequence.

Will participation in this study affect my routine healthcare, or the waiting period for treatment for my eating disorder if I am currently on a waiting list?
No. Participation in this study will have no impact on your treatment as usual, or waiting time if you are currently awaiting treatment for your eating disorder. We fully encourage you to begin treatment as provided by a health care professional as soon as it becomes available to you. We simply ask that you inform us of any changes to your treatment or medication while you are partaking in the study.

What if there is a problem?
If you have a concern about any aspect of the study, please ask the researcher (gemma.gordon@kcl.ac.uk, 0207 848 0183) who will do their best to answer your questions.

What if I wish to make a complaint?
If you remain unhappy and wish to formally complain, complaints to the IoPPN should be addressed to Dr Gill Dale. Director of Research Quality; Head, Joint R&D Office of South London and Maudsley NHS Foundation Trust and Institute of Psychiatry, Psychology & Neuroscience (IoPPN), P005, Institute of Psychiatry, Psychology & Neuroscience (IoPPN), King's College London, De Crespigny Park, London SE5 8AF. NHS complaints will follow NHS complaints procedures.

Other sources of support for your eating disorder
To access support and treatment, please see your Eating Disorders clinician or your GP who will be able to advise you and refer you to the right service for you. You can also

For peer review only - http://bmjopen.bmj.com/site/about/guidelines.xhtml

obtain further information and support from www.beateatingdisorders.org.uk, the national Eating Disorders charity.

Should you wish to speak to someone outside of the university, please talk to your Eating Disorders clinician, GP, the Beat helpline, and/or one of the study researchers who is happy to liaise on your behalf if you so wish. The eating disorders charity Beat provides helplines for adults and young people which offer support and information to sufferers, carers and professionals. Further information can be found on their website www.beateatingdisorders.org.uk, or by ringing their helpline 0808 801 0677**.**

Will my taking part in the study be kept confidential?
Your personal information and the data we collect from you will remain confidential at all times. It will also remain anonymous to everyone apart from the primary researchers. Manual files will be locked securely in a filing cabinet, which will be kept in a locked office in the KCL Section of Eating Disorders, Department of Psychological Medicine, IoPPN, and all electronic files will be password protected. All information which is collected during the course of the research will be kept strictly confidential according to the General Data Protection Regulation (GDPR), brought into effect on 25th May, 2018. This new legislation creates some new rights for individuals to better reflect data protection challenges in the modern digital age, as well as strengthening some of the rights that currently exist under the Data Protection Act 1998.

How will my personal data be used and what are my rights?
King's College London is the sponsor for this study based in the United Kingdom. We will be using information from you in order to undertake this study and will act as the data controller for this study. This means that we are responsible for looking after your information and using it properly. King's College London will keep identifiable information about you for four years after the study has finished.
Your rights to access, change or move your information are limited, as we need to manage your information in specific ways in order for the research to be reliable and accurate. If you withdraw from the study, we will keep the information about you that we have already obtained. To safeguard your rights, we will use the minimum personally-identifiable information possible. You can find out more about how we use your information by contacting the Study Coordinator Gemma Gordon.

KCL will use your name and contact details to contact you about the research study, and make sure that relevant information about the study is recorded for your care, and to oversee the quality of the study. Individuals from KCL and regulatory organisations may look at your medical and research records to check the accuracy of the research study. SLaM will pass these details to KCL along with the information collected from you. The only people in KCL who will have access to information that identifies you will be people who need to contact you regarding your participation or audit the data collection process. The people who analyse the information will not be able to identify you and will not be able to find out your name or contact details. KCL will keep identifiable information about you from this study for four years after the study has finished.

Involvement of the General Practitioner (GP)

For peer review only - http://bmjopen.bmj.com/site/about/guidelines.xhtml

As a matter of courtesy and in the interest of your wellbeing, we may let your GP know about your participation in the study, and may request your permission to send them a letter when you enroll. If you agree to this, you will be asked to provide us with your GP's contact details so that we can send them a letter with details of the research.

Insurance/indemnity
Standard KCL insurance and NHS indemnity arrangements apply.

Involvement of the insurance company
If you have private medical insurance, you should inform your insurance company that you are taking part in this study.

Will any genetic tests be done?
No.

What will happen to the results of the research study?
You will be offered the opportunity to be informed about your individual results once the data for all participants has been collected. If you want written feedback of the study's findings you can contact the researcher (gemma.gordon@kcl.ac.uk) for a lay summary. The results will be included in an examined postgraduate report, presented as part of a postgraduate presentation, and sent to a medical journal for publication. Your participation in the study will not be disclosed.

Who is organising and funding the research?
This study is being funded by King's College London.

Who has reviewed the study?
All research in the NHS is looked at by an independent group of people, called a Research Ethics Committee, to protect your interests. This study has been reviewed and given favourable opinion by North West -Liverpool East Research Ethics Committee.

Further information and contact details
If you have any questions or require more information about this study, please contact the researcher using the following contact details:
Gemma Gordon (gemma.gordon@kcl.ac.uk) (0207 848 5608)
Section of Eating Disorders, Department of Psychological Medicine
KCL Institute of Psychiatry, Psychology and Neuroscience,
16 De Crespigny Park
London, SE5 8AF

Appendix C: Consent form for all participants

CONSENT FORM

IRAS Project ID: 244170
Research Ethics Committee reference number: 18/NW/0648

Please complete this form after you have read the information

For peer review only - http://bmjopen.bmj.com/site/about/guidelines.xhtml

sheet and listened to an explanation about the research.

Title of Study: ICARUS - An Investigation of Approach Bias Modification Training (ABM) and Transcranial Direct Current Stimulation (tDCS) in Binge Eating Disorder
Name of Researcher: Gemma Gordon

NHS South London and Maudsley NHS Foundation Trust

Thank you for considering taking part in this research. The person organising the research must explain the project to you before you agree to take part. If you have any questions arising from the information sheet or explanation already given to you, please ask the researcher before you decide whether to join in. You will be given a copy of this consent form to keep and refer to at any time.

Please initial box

1. I confirm that I have read the information sheet dated 18.09.2018 (version 3) for the above study. I have had the opportunity to consider the information, ask questions and have had these answered satisfactorily. ☐

2. I understand that my participation is voluntary and that I am free to withdraw at any time without giving a reason, without my medical care or legal rights being affected. ☐

3. I consent to the processing of my personal information for the purposes explained to me. I understand that such information will be handled in accordance with the terms of the General Data Protection Regulation (GDPR) 2018. I understand that confidentiality and anonymity will be maintained and it will not be possible to identify me in any publications. ☐

4. I know that if I would like to, I can contact the research team and request a written summary of the study findings. ☐

5. I understand that I must not take part if I have any of the conditions listed in the exclusion criteria. ☐

6. I understand that during study participation, I must inform the researcher of any changes in my medication or of any new medical diagnoses made. ☐

7. I agree to my General Practitioner (GP) being informed of my participation in this study. ☐

8. **I consent/do not consent** to be contacted in the future by King's College London researchers who would like to invite me to participate in follow up studies to this project, or in future studies of a similar nature. ☐

9. I agree to take part in the above study. ☐

For peer review only - http://bmjopen.bmj.com/site/about/guidelines.xhtml

---

<u>Participant's Statement:</u>

I ____

agree that the research project named above has been explained to me to my satisfaction and I agree to take part in the study. I have read both the notes written above and the Information Sheet about the project, and understand what the research study involves.

Signed Date

<u>Investigator's Statement:</u>

I ____

Confirm that I have carefully explained the nature, demands and any foreseeable risks (where applicable) of the proposed research to the participant.

Signed Date

<u>For administration purposes, please indicate your preference below</u>

Q: In what format would you like to complete the at-home assessment questionnaires?

Tick '√' in one box to indicate your choice

1. I would like to complete the assessment questionnaires online ☐

2. I would like to receive a hardcopy paper version of the questionnaires in a stamped addressed envelope, to complete and post back to the researcher within 36 hours of the in-person assessment visit. ☐

Enquiries:
Gemma Gordon (gemma.gordon@kcl.ac.uk)
Department of Psychological Medicine
Section of Eating Disorders
KCL Institute of Psychology, Psychiatry and Neuroscience,
16 De Crespigny Park
London, SE5 8AF
Phone: 0207 848 5608

For peer review only

For peer review only

For peer review only - http://bmjopen.bmj.com/site/about/guidelines.xhtml

SPIRIT
STANDARD PROTOCOL ITEMS: RECOMMENDATIONS FOR INTERVENTIONAL TRIALS

SPIRIT 2013 Checklist: Recommended items to address in a clinical trial protocol and related documents*

Section/item	Item No	Description	Addressed on page number
**Administrative information**			
Title	1	Descriptive title identifying the study design, population, interventions, and, if applicable, trial acronym	_______1______
Trial registration	2a	Trial identifier and registry name. If not yet registered, name of intended registry	_______1______
	2b	All items from the World Health Organization Trial Registration Data Set	______________
Protocol version	3	Date and version identifier	______1_______
Funding	4	Sources and types of financial, material, and other support	______12_______
Roles and responsibilities	5a	Names, affiliations, and roles of protocol contributors	______12______
	5b	Name and contact information for the trial sponsor	______12_______
	5c	Role of study sponsor and funders, if any, in study design; collection, management, analysis, and interpretation of data; writing of the report; and the decision to submit the report for publication, including whether they will have ultimate authority over any of these activities	______12______
	5d	Composition, roles, and responsibilities of the coordinating centre, steering committee, endpoint adjudication committee, data management team, and other individuals or groups overseeing the trial, if applicable (see Item 21a for data monitoring committee)	_____11____

For peer review only

**Introduction**			
Background and rationale	6a	Description of research question and justification for undertaking the trial, including summary of relevant studies (published and unpublished) examining benefits and harms for each intervention	_____2-4___
	6b	Explanation for choice of comparators	______4__
Objectives	7	Specific objectives or hypotheses	______4___
Trial design	8	Description of trial design including type of trial (eg, parallel group, crossover, factorial, single group), allocation ratio, and framework (eg, superiority, equivalence, noninferiority, exploratory)	______5______
**Methods: Participants, interventions, and outcomes**			
Study setting	9	Description of study settings (eg, community clinic, academic hospital) and list of countries where data will be collected. Reference to where list of study sites can be obtained	_____6_____
Eligibility criteria	10	Inclusion and exclusion criteria for participants. If applicable, eligibility criteria for study centres and individuals who will perform the interventions (eg, surgeons, psychotherapists)	______5_____
Interventions	11a	Interventions for each group with sufficient detail to allow replication, including how and when they will be administered	____9-10___
	11b	Criteria for discontinuing or modifying allocated interventions for a given trial participant (eg, drug dose change in response to harms, participant request, or improving/worsening disease)	____11___
	11c	Strategies to improve adherence to intervention protocols, and any procedures for monitoring adherence (eg, drug tablet return, laboratory tests)	____11____
	11d	Relevant concomitant care and interventions that are permitted or prohibited during the trial	____4, 11___
Outcomes	12	Primary, secondary, and other outcomes, including the specific measurement variable (eg, systolic blood pressure), analysis metric (eg, change from baseline, final value, time to event), method of aggregation (eg, median, proportion), and time point for each outcome. Explanation of the clinical relevance of chosen efficacy and harm outcomes is strongly recommended	____6-9___

For peer review only - http://bmjopen.bmj.com/site/about/guidelines.xhtml

Participant timeline	13	Time schedule of enrolment, interventions (including any run-ins and washouts), assessments, and visits for participants. A schematic diagram is highly recommended (see Figure)	___6__
Sample size	14	Estimated number of participants needed to achieve study objectives and how it was determined, including clinical and statistical assumptions supporting any sample size calculations	____5___
Recruitment	15	Strategies for achieving adequate participant enrolment to reach target sample size	___11__

Methods: Assignment of interventions (for controlled trials)

Allocation:

Sequence generation	16a	Method of generating the allocation sequence (eg, computer-generated random numbers), and list of any factors for stratification. To reduce predictability of a random sequence, details of any planned restriction (eg, blocking) should be provided in a separate document that is unavailable to those who enrol participants or assign interventions	____5-6___
Allocation concealment mechanism	16b	Mechanism of implementing the allocation sequence (eg, central telephone; sequentially numbered, opaque, sealed envelopes), describing any steps to conceal the sequence until interventions are assigned	____6___
Implementation	16c	Who will generate the allocation sequence, who will enrol participants, and who will assign participants to interventions	____5____
Blinding (masking)	17a	Who will be blinded after assignment to interventions (eg, trial participants, care providers, outcome assessors, data analysts), and how	___5____
	17b	If blinded, circumstances under which unblinding is permissible, and procedure for revealing a participant's allocated intervention during the trial	___6____

Methods: Data collection, management, and analysis

Data collection methods	18a	Plans for assessment and collection of outcome, baseline, and other trial data, including any related processes to promote data quality (eg, duplicate measurements, training of assessors) and a description of study instruments (eg, questionnaires, laboratory tests) along with their reliability and validity, if known. Reference to where data collection forms can be found, if not in the protocol	____6-8____

	18b	Plans to promote participant retention and complete follow-up, including list of any outcome data to be collected for participants who discontinue or deviate from intervention protocols	_____10-11____
Data management	19	Plans for data entry, coding, security, and storage, including any related processes to promote data quality (eg, double data entry; range checks for data values). Reference to where details of data management procedures can be found, if not in the protocol	_____10-11_____
Statistical methods	20a	Statistical methods for analysing primary and secondary outcomes. Reference to where other details of the statistical analysis plan can be found, if not in the protocol	_____10_____
	20b	Methods for any additional analyses (eg, subgroup and adjusted analyses)	_____10______
	20c	Definition of analysis population relating to protocol non-adherence (eg, as randomised analysis), and any statistical methods to handle missing data (eg, multiple imputation)	______10______
**Methods: Monitoring**			
Data monitoring	21a	Composition of data monitoring committee (DMC); summary of its role and reporting structure; statement of whether it is independent from the sponsor and competing interests; and reference to where further details about its charter can be found, if not in the protocol. Alternatively, an explanation of why a DMC is not needed	_____10_____
	21b	Description of any interim analyses and stopping guidelines, including who will have access to these interim results and make the final decision to terminate the trial	_____11______
Harms	22	Plans for collecting, assessing, reporting, and managing solicited and spontaneously reported adverse events and other unintended effects of trial interventions or trial conduct	_____11_____
Auditing	23	Frequency and procedures for auditing trial conduct, if any, and whether the process will be independent from investigators and the sponsor	_______n/a____
**Ethics and dissemination**			
Research ethics approval	24	Plans for seeking research ethics committee/institutional review board (REC/IRB) approval	______1, 13____

For peer review only - http://bmjopen.bmj.com/site/about/guidelines.xhtml

Protocol amendments	25	Plans for communicating important protocol modifications (eg, changes to eligibility criteria, outcomes, analyses) to relevant parties (eg, investigators, REC/IRBs, trial participants, trial registries, journals, regulators)	_____1, 12_____
Consent or assent	26a	Who will obtain informed consent or assent from potential trial participants or authorised surrogates, and how (see Item 32)	_____12_____
	26b	Additional consent provisions for collection and use of participant data and biological specimens in ancillary studies, if applicable	_____n/a_____
Confidentiality	27	How personal information about potential and enrolled participants will be collected, shared, and maintained in order to protect confidentiality before, during, and after the trial	____10-11____
Declaration of interests	28	Financial and other competing interests for principal investigators for the overall trial and each study site	_____12______
Access to data	29	Statement of who will have access to the final trial dataset, and disclosure of contractual agreements that limit such access for investigators	_____ 10_____
Ancillary and post-trial care	30	Provisions, if any, for ancillary and post-trial care, and for compensation to those who suffer harm from trial participation	______11_____
Dissemination policy	31a	Plans for investigators and sponsor to communicate trial results to participants, healthcare professionals, the public, and other relevant groups (eg, via publication, reporting in results databases, or other data sharing arrangements), including any publication restrictions	______1, 11_____
	31b	Authorship eligibility guidelines and any intended use of professional writers	______12______
	31c	Plans, if any, for granting public access to the full protocol, participant-level dataset, and statistical code	______n/a______
**Appendices**			
Informed consent materials	32	Model consent form and other related documentation given to participants and authorised surrogates	______24______
Biological specimens	33	Plans for collection, laboratory evaluation, and storage of biological specimens for genetic or molecular analysis in the current trial and for future use in ancillary studies, if applicable	______n/a______

For peer review only - http://bmjopen.bmj.com/site/about/guidelines.xhtml

*It is strongly recommended that this checklist be read in conjunction with the SPIRIT 2013 Explanation & Elaboration for important clarification on the items. Amendments to the protocol should be tracked and dated. The SPIRIT checklist is copyrighted by the SPIRIT Group under the Creative Commons "Attribution-NonCommercial-NoDerivs 3.0 Unported" license.

For peer review only

CONSORT 2010 checklist of information to include when reporting a randomised trial*

Section/Topic	Item No	Checklist item	Reported on page No
**Title and abstract**			
	1a	Identification as a randomised trial in the title	1
	1b	Structured summary of trial design, methods, results, and conclusions (for specific guidance see CONSORT for abstracts)	1(no results)
**Introduction**			
Background and objectives	2a	Scientific background and explanation of rationale	2-4
	2b	Specific objectives or hypotheses	4-5
**Methods**			
Trial design	3a	Description of trial design (such as parallel, factorial) including allocation ratio	5
	3b	Important changes to methods after trial commencement (such as eligibility criteria), with reasons	n/a
Participants	4a	Eligibility criteria for participants	5
	4b	Settings and locations where the data were collected	6
Interventions	5	The interventions for each group with sufficient details to allow replication, including how and when they were actually administered	9-10
Outcomes	6a	Completely defined pre-specified primary and secondary outcome measures, including how and when they were assessed	6-9
	6b	Any changes to trial outcomes after the trial commenced, with reasons	n/a
Sample size	7a	How sample size was determined	5
	7b	When applicable, explanation of any interim analyses and stopping guidelines	11
Randomisation:			
Sequence generation	8a	Method used to generate the random allocation sequence	5-6
	8b	Type of randomisation; details of any restriction (such as blocking and block size)	5-6
Allocation concealment mechanism	9	Mechanism used to implement the random allocation sequence (such as sequentially numbered containers), describing any steps taken to conceal the sequence until interventions were assigned	6
Implementation	10	Who generated the random allocation sequence, who enrolled participants, and who assigned participants to interventions	5
Blinding	11a	If done, who was blinded after assignment to interventions (for example, participants, care providers, those	5

For peer review only - http://bmjopen.bmj.com/site/about/guidelines.xhtml

		assessing outcomes) and how	
	11b	If relevant, description of the similarity of interventions	4
Statistical methods	12a	Statistical methods used to compare groups for primary and secondary outcomes	10
	12b	Methods for additional analyses, such as subgroup analyses and adjusted analyses	10
**Results**			
Participant flow (a diagram is strongly recommended)	13a	For each group, the numbers of participants who were randomly assigned, received intended treatment, and were analysed for the primary outcome	n/a
	13b	For each group, losses and exclusions after randomisation, together with reasons	n/a
Recruitment	14a	Dates defining the periods of recruitment and follow-up	n/a
	14b	Why the trial ended or was stopped	n/a
Baseline data	15	A table showing baseline demographic and clinical characteristics for each group	n/a
Numbers analysed	16	For each group, number of participants (denominator) included in each analysis and whether the analysis was by original assigned groups	n/a
Outcomes and estimation	17a	For each primary and secondary outcome, results for each group, and the estimated effect size and its precision (such as 95% confidence interval)	n/a
	17b	For binary outcomes, presentation of both absolute and relative effect sizes is recommended	n/a
Ancillary analyses	18	Results of any other analyses performed, including subgroup analyses and adjusted analyses, distinguishing pre-specified from exploratory	n/a
Harms	19	All important harms or unintended effects in each group (for specific guidance see CONSORT for harms)	n/a
**Discussion**			
Limitations	20	Trial limitations, addressing sources of potential bias, imprecision, and, if relevant, multiplicity of analyses	n/a
Generalisability	21	Generalisability (external validity, applicability) of the trial findings	n/a
Interpretation	22	Interpretation consistent with results, balancing benefits and harms, and considering other relevant evidence	n/a
**Other information**			
Registration	23	Registration number and name of trial registry	1
Protocol	24	Where the full trial protocol can be accessed, if available	n/a
Funding	25	Sources of funding and other support (such as supply of drugs), role of funders	12

*We strongly recommend reading this statement in conjunction with the CONSORT 2010 Explanation and Elaboration for important clarifications on all the items. If relevant, we also recommend reading CONSORT extensions for cluster randomised trials, non-inferiority and equivalence trials, non-pharmacological treatments, herbal interventions, and pragmatic trials. Additional extensions are forthcoming: for those and for up to date references relevant to this checklist, see www.consort-statement.org.

For peer review only - http://bmjopen.bmj.com/site/about/guidelines.xhtml
For peer review only
